# Bis‐[3]Ferrocenophanes with Central >E−E’< Bonds (E, E’=P, SiH): Preparation, Properties, and Thermal Activation

**DOI:** 10.1002/open.201900182

**Published:** 2019-06-26

**Authors:** Stefan Isenberg, Stefan Weller, Denis Kargin, Srećko Valić, Brigitte Schwederski, Zsolt Kelemen, Clemens Bruhn, Kristijan Krekić, Martin Maurer, Christoph M. Feil, Martin Nieger, Dietrich Gudat, László Nyulászi, Rudolf Pietschnig

**Affiliations:** ^1^ Institute for Chemistry and CINSaT University of Kassel Heinrich Plett-Straße 40 34132 Kassel Germany; ^2^ Institute for Inorganic Chemistry University of Stuttgart Pfaffenwaldring 55 70550 Stuttgart Germany; ^3^ Ruđer Bošković Institute Bijenička cesta 54 10000 Zagreb Croatia; ^4^ Department of Inorganic and Analytical Chemistry and MTA-BME Computation Driven Chemistry Research Group Budapest University of Technology and Economics Szent Gellért tér 4 1111 Budapest Hungary; ^5^ Department of Chemistry University of Helsinki P.O Box 55 Helsinki 00014 Finland

**Keywords:** phosphorus, ferrocene, homolytic bond cleavage, thermolysis, radicals

## Abstract

A series of bis‐[3]ferrocenophanes of the general type Fe(C_5_H_4_E’)_2_E−E(E'C_5_H_4_)_2_Fe (E=P, SiH and E’=P*t*Bu, N*neo*Pentyl, NSi(CH_3_)_3_) with an isolobal molecular framework have been prepared and characterized by heteronuclear NMR spectroscopy and X‐ray crystallography. The thermal dissociation behavior with respect to homolytic fission of the central bond generating phosphorus centered radicals was investigated using EPR spectroscopy and quantum chemical calculations.

## Graphical abstract

**Give P a chance**: Phosphorus‐rich bisferrocenophanes have been prepared and characterized by heteronuclear NMR spectroscopy and X‐ray crystallography. These species undergo thermal activation with homolytic cleavage of the central P−P bond.

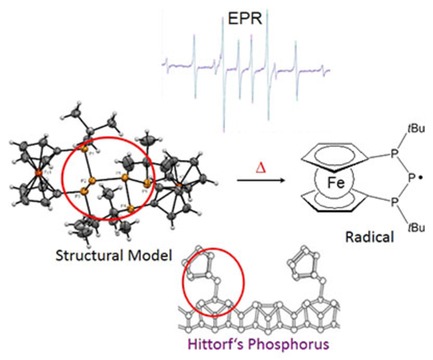

## 1 Introduction

Recently, phosphorus modifications such as black phosphorus and especially its exfoliated version, phosphorene, attracted substantial interest because of their unique electronic and thermoelectric material properties.^1^ While in most crystalline phosphorus modifications the relative orientation of the phosphorus lone‐pairs is constrained by the three‐dimensional lattice, molecular organopolyphosphorus frameworks can give rise to rather complex mixtures of diastereomers. Nevertheless, organopolyphosphorus frameworks may be regarded as molecular models for sections of phosphorus modifications such as the (>P)_2_P−P(P<)_2_ motif found in the rims and trenches of black phosphorus, or as connector units between the orthogonal or parallel tubes in Hittorf's and Ruck's variants of violet phosphorus (Chart 1).^2^ However, the mobility at the central bond of the (>P)_2_P−P(P<)_2_ fragments is limited in the elemental allotropes by the rigidity of the solid matrix, while molecular compounds containing this particular unit may open a way to investigate the properties of this unique structural unit as well. In this respect, unsupported (>P)_2_P−P(P<)_2_ fragments, where the central P−P bond is not reinforced by additional bonding between the two halves of the fragment, would be attractive models to study the reactivity of this particular bond.

In previous work, we have shown that formally linking two terminal phosphorus atoms by a ferrocene fragment can provide a suitable template for the assembly of such cyclic structures.^3^ As a consequence of their conformational constraints and rigidity, ferrocenophanes with phosphorus‐rich fragments in the *ansa*‐bridge can often be isolated as single diastereomers.3a–3c, 4

Beyond its beneficial structural features, the ferrocene unit usually provides reversible redox activity, where oxidation at iron may entail electron transfer with the phosphorus atoms.^5^ Such oxidized systems could be related to cationic polyphosphorus compounds as described by Weigand and Burford *et al*.^6^


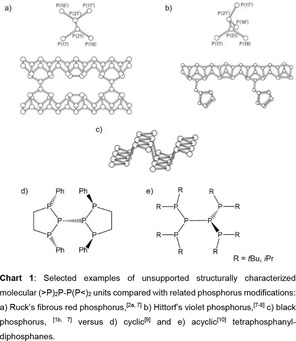


Capping both halves of the (>P)_2_P−P(P<)_2_ framework by ferrocene units would lead to bis‐[3]ferrocenophanes where the two *ansa*‐bridges are connected via a single bond. Compounds of this type are as yet unprecedented in the literature.

Here, we explore synthesis, structure and homolytic bond cleavage of an electro‐neutral version of the (>P)_2_P−P(P<)_2_ motif embedded in a bis‐ferrocenophane framework (compound **4**, Chart 2). Moreover, we extended our investigation to a series of structural analogs in which the outer P‐atoms are formally replaced by N‐atoms (**8**, **9**), or the central P‐atoms by isolobal SiH units (**6**, **7**), respectively (Chart 2). In view of electronic coupling between the redox active ferrocenyl unit and adjacent phosphane moieties,^11^ the radical species generated from such fragmentation processes may be envisaged to exhibit interesting electronic structures.


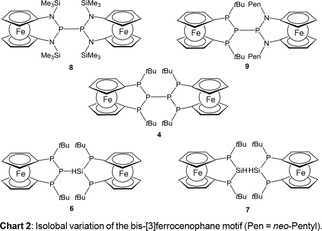


## 2 Results and Discussion

### 2.1 Synthesis of a P_6_‐bis‐[3]Ferrocenophane

**Scheme 1:**
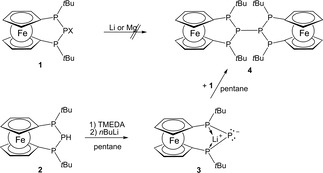
Formation of bis‐[3]ferrocenophane **4** via phosphanide **3** (X=Cl,Br; TMEDA=tetramethylethylenediamine).

We anticipated that the bis‐[3]ferrocenophane **4** (Scheme 1) would be readily accessible by reductive coupling of two molecules of P‐halo‐[3]ferrocenophane **1**.3c However, reaction of **1** with metals such as magnesium or lithium under various conditions did not furnish the envisaged product, but yielded minor amounts of ferrocenophane **2** instead, which had previously been obtained from the same starting material and LiAlH_4_.3c

**Figure 1:**
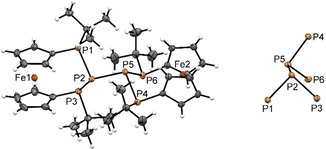
Molecular structure of **4** (left) and conformation of its P_3_‐P_3_ motif. Ellipsoids are drawn at 30 % probability level.

Trying to avoid a radical mechanism, which possibly occurred in the reduction of **1** with metals, we attempted to create the central PP bond by employing a heteropolar approach. To this end, we reacted lithium phosphanide **3** 12 with one equiv. of **1** (Scheme 1). As intended, this reaction produced a yellow‐orange solution of the hexaphospha‐bis‐[3]ferrocenophane **4** (Scheme 1), which was purified by recrystallization from toluene. Single‐crystal X‐ray diffraction analysis revealed that compound **4** crystallizes in a monoclinic space group (P 2_1_/n). The crystal contains isolated molecules (Figure 1) showing the envisaged bis‐ferrocenophane structure with orthogonal orientation of the ferrocene units (torsion angle between the Cp(cent)−Fe−Cp(cent) axes of both Fc units about 94°) featuring tilt angles of 3.48(3)° and 4.0(1)° respectively. On the whole, the structural features of the *ansa*‐rings in each half of **4** match those of the *trans*‐isomer of the corresponding mono‐[3]ferrocenophanes we had reported earlier.3c It has to be noted that the distances between the central phosphorus atoms in the *ansa‐*bridges and the closest iron centers (P2⋅⋅⋅Fe1 3.7313(7) Å, P5⋅⋅⋅Fe2 3.7542(7) Å) are somewhat shorter than the sum of the van‐der‐Waals radii (4.34 Å)^13^, but longer than in ferrocenophane **1** (Fe⋅⋅⋅PX; X=Cl: 3.552(3) Å, Br: 3.544(3) Å).3c

As anticipated, the structural features of the P_6_‐skeleton of **4** bear close similarity to the P_2_P–PP_2_‐fragments linking the tubular subunits in Hittorf's (violet) phosphorus.^7^, ^8^ Both species exhibit a central P−P bond that adopts a *gauche* conformation (dihedral angles 83.9°8a in Hittorf's P, 92.8(1)° in **4**) and is shorter than the adjacent bonds (2.178 vs. 2.199 to 2.206 Å in Hittorf's P8a, 2.229(1) Å vs. 2.262(1) to 2.274(1) Å in **4**). The same bond length pattern was also predicted computationally for the ωB97XD/6‐31+G* optimized structure of **4** (see Supporting Information) and elusive parent P_6_H_8_,^14^ and observed in other known molecules with P_2_P−PP_2_ structural motifs.^7^

**Figure 2:**
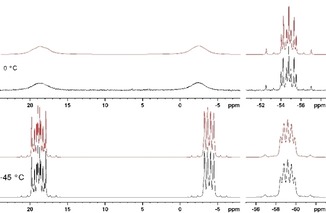
Measured (black traces) and simulated (red traces, see SI for details) ^31^P{^1^H} NMR spectra of **4** recorded at −45 °C (bottom) and 0 °C (top).

Solution ^31^P NMR spectra of **4** display line broadening effects and higher order patterns. The resonance of the central phosphorus atoms appears in the ambient temperature spectrum as a symmetric multiplet centered at −44.1 ppm (in toluene‐d_8_), while the terminal phosphorus atoms give rise to a broad, structureless signal which extends from 0 to 30 ppm and exhibits maxima around 8 and 25 ppm. Assuming that the observed line broadening arises from a dynamic process, we performed a variable temperature NMR study on a CDCl_3_ solution of **4**, as the solubility in this solvent is higher than in toluene.^15^ As expected, the broad resonances transform eventually into two well separated multiplets at 18.8 and −4.0 ppm upon cooling to −45 °C, and the observed spectral pattern could be successfully simulated as an AA′MM′XX′ spin system (Figure 2). NMR experiments above room temperature revealed coalescence of the broad signals due to dynamic averaging of the A,A′ and M,M′ signals

The ^1^H NMR spectrum of **4** displays at −45 °C signals attributable to two distinguishable *t*Bu‐groups and two Cp‐rings, which undergo pairwise coalescence upon warming and have at room temperature merged into narrow dynamically averaged resonances. Dynamic lineshape simulations allowed us to relate the observed spectral changes to a single dynamic process. Determination of the activation parameters from an Eyring plot based on the fitted rate constants yielded values of Δ*H* ^#^=54.9(11) kJ mol^−1^ and Δ*S* ^#^=17.7(4) J (K mol)^−1^ (see the Supporting Information for details). We attribute the observed dynamics to a librational motion of the two Fe(CpP*t*Bu)_2_P fragments relative to each other, which involves mutual interconversion of the two terminal phosphanyl fragments attached to each central phosphorus atom. Similar processes have been observed for *t*Bu_2_P‐P*t*Bu_2_ and acyclic analogues of **4** reported by Kovacs10b and Fritz,^16^ or in a symmetrically substituted bis‐diazaphosphole, respectively.^17^ It should be noted that Δ*H* ^#^ of the librational process comes close to the calculated ωB97XD/6‐31G* inversion barrier at the central phosphorus of 71.1 kJ/mol). This value is significantly smaller than the usual inversion barriers for the phosphorus atoms in phosphanes (146 kJ/mol for PH_3_),^18^ or the terminal phosphorus atoms in **4** (133.5 kJ/mol), respectively. A lowering of the inversion barrier with respect to these reference values is, however, quite common for branched oligophosphanes.^19^

A comparison to the aforementioned related hexaphosphanes reveals that the ^31^P NMR chemical shifts for Weigand's compound (−41.8 ppm (2 P), 13.2 ppm (4 P))^9^ match those observed for **4** in the high temperature limit, while Wiberg's silylated system resonates at higher field.^20^ The acyclic congeners reported by Kovacs10b and Fritz^16^ give rise to ^31^P NMR spectra which display similar signals as **4** at low temperature and show also a similar temperature dependence.

### 2.2 Synthesis of P_5_(SiH)‐bis‐[3]Ferrocenophane

**Scheme 2:**
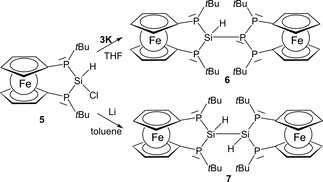
Synthesis of bis‐[3]ferrocenophanes **6** and **7**.

A variation of the structural motif in hexaphospha‐bis‐[3]ferrocenophane **4** can be achieved by formal substitution of the central phosphorus atoms by isolobal units such as a silylidyne (SiH) fragment. While the attempt to synthesize a pentaphosphasila‐bis‐[3]ferrocenophane **6** (Scheme 2) by coupling of lithium phosphanide **3** with the previously published hydridochlorosilane **5** 3b failed, salt metathesis of **5** with the recently reported potassium phosphanide **3 K** 12 readily afforded **6** as yellow solid (Scheme 2).

**Figure 3:**
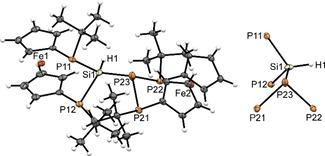
Molecular structure (left) of **6** and conformation of its P_2_Si(H)‐P_3_ motif (right). Ellipsoids are drawn at 30 % probability level.

A single‐crystal X‐ray diffraction study confirmed the molecular structure of **6**, which is isostructural to that of **4** (Figure 3). The sum of PSiP angles around Si1 (319.7(3)°) is larger than the sum of PPP angles around P2 in **4**. Furthermore, the central Si1‐P23 bond (2.302(3) Å) is no longer the shortest Si−P bond of the scaffold, exceeding contacts Si1‐P11 (2.264(3) Å) and Si1‐P12 (2.269(3) Å). Compared to other known P‐Si‐P bridged [3]ferrocenophanes (P‐Si 2.2380(5)–2.255(1) Å),3b, 3d these bonds are slightly elongated.

**Figure 4:**
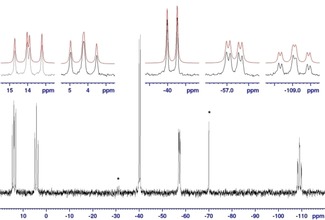
^31^P{^1^H} NMR spectrum of **6** at −40 °C with expansions of the individual multiplets. Signals labelled with an asterisk are due to impurities / decomposition products (Fe(CpPH*t*Bu)_2_). Red traces represent the result of a spectral simulation as an AFHMX spin system; simulation parameters (*J* given as absolute values): *δ*_A_= 14.0 (P‐**P** *t*Bu), *δ*_F_=4.3 (P‐**P** *t*Bu), *δ*_H_=‐40.3 (Si‐P*t*Bu), *δ*_M_=−57.3 (Si‐P*t*Bu), *δ*_X_=−109.1 (>PSi(H)<), ^2^ *J*_AF_=139, ^1^ *J*_AX_=165, ^1^ *J*_FX_=153, ^2^ *J*_HM_=113, ^2^ *J*_MX_=32.

The ^31^P NMR spectra of **6** reveal a similar temperature dependence as in case of **4**. The central phosphorus atom of the triphosphane part of the P_2_(SiH)‐P_3_ backbone appears in the room temperature spectrum as a triplet of triplets at −106.6 ppm with splittings of 163 Hz and 16 Hz due to coupling with the adjacent and remote ferrocene bonded phosphorus atoms, which themselves give rise to broad resonances between −59 to −34 ppm (*ν*_1/2_≈1300 Hz, Si‐bound P‐atoms3b) and 2 to 20 ppm (*ν*_1/2_≈1600 Hz, P‐bound terminal phosphorus atoms). The broad signals sharpen when the temperature is raised, and below room temperature eventually decoalesce into four multiplets, which display at −40 °C the signal pattern of a nearly first‐order AFHMX spin system (Figure 4). The marked differences in the geminal (^*2*^ *J*) couplings between the three silicon‐bound phosphorus atoms reflects presumably the dependence of the coupling interaction on the relative orientation of the phosphorus lone‐pairs and has previously also been observed for the *cis*‐*trans*‐isomers of **5**.3b The sensitivity of ^2^ *J*_PP_ couplings towards conformational changes is well known both in general^21^ and in the chemistry of polyphosphorus compounds,^22^ and the observation of large magnitudes for “through space” couplings has been attributed to the overlap of the phosphorus lone‐pairs in space.^23^ Similar temperature dependent changes as in the ^31^P NMR data are also visible for the Cp‐ and *t*Bu‐resonances in the ^1^H NMR spectra; e. g. a spectrum recorded at −40 °C displays four signals for the *tert*‐butyl protons which coalesce pairwise to give the expected two averaged signals at higher temperature. Simulation of dynamic lineshapes for the ^31^P NMR spectra recorded between −40 and 62 °C allowed us as in case of **4** to relate the spectral changes to a single dynamic process, and an Eyring plot of the fitted rate constants yielded values of Δ*H* ^#^=59.7(8) kJ mol^−1^ and Δ*S* ^#^=23.7(3) J (K mol)^−1^ (cf. SI for details).

As in **4**, the observed dynamics are consistent with the occurrence of a librational motion of the two halves of the molecule. The observed enthalpy of activation is in this case significantly lower than the DFT calculated (ωB97XD/6‐31G*) inversion barrier of 95.4 kJ/mol for the central phosphorus atom. The inversion barrier for the terminal phosphorus atoms of the P_3_ part is still higher at 125.1 kJ/mol, while inversion of the terminal phosphorus atoms of the silyl alkyl phosphane units requires the lowest activation barrier (75.3 kJ/mol).

### 2.3 Synthesis of P_4_(SiH)_2_‐bis‐[3]Ferrocenophane

The synthesis of Si−Si coupled tetraphospha‐bis‐[3]ferrocenophane **7** is accomplished by lithium mediated coupling of literature known hydridochlorosilane **5** 3b at elevated temperature (170 °C). Bis‐[3]ferrocenophane **7** is obtained as yellow solid in moderate yield (Scheme 2).

The ^31^P NMR spectrum of **7** in toluene solution at room temperature displays two resonances of an AB spin system at −44.9 ppm and −50.1 ppm (^2^ *J*_PP_=123 Hz), which exhibit dynamic broadening when the temperature is raised and are close to coalescence at 75 °C (cf. SI for details). The ^1^H NMR spectrum displays at room temperature two signals attributable to *t*Bu‐groups and a complex signal pattern for the cyclopentadienyl protons, which merge into a single resonance (*t*Bu) or the pattern of four signals expected for a single Cp‐unit at higher temperature, respectively. The dynamically induced changes are, as in the previous cases, attributable to a librational motion of the two P_2_SiH‐fragments. We did not determine the activation barrier for this process, but the calculated energy barrier for P inversion of 79.9 kJ/mol (ωB97XD/6‐31G*) matches the value for the analogous process in **6**.

**Figure 5:**
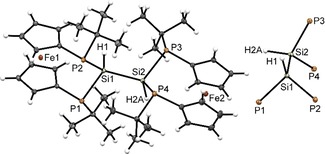
Molecular structure (left) of **7** and conformation of its P_2_Si(H)‐Si(H)P_2_ motif (right). Ellipsoids are drawn at 30 % probability level.

A single crystal XRD study revealed that **7** is likewise isostructural to **6** and **4**. The sum of P−Si−(P,Si) angles around each silicon atom (Si1 328.9(1)°, Si2 327.5(1)°) is even larger than in **6**. The Si1‐Si2 distance is 2.3828(9) Å, which is even longer than in a sterically challenged disilane (2.354(3) Å).^24^ All other bond lengths and angles in **7** are comparable to **4** and **6** and will not be discussed in detail (Figure 5).

### 2.4 Synthesis of P_2_N_4_‐ and P_4_N_2_‐bis‐[3]Ferrocenophanes

**Scheme 3:**
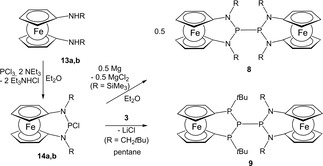
Synthesis of bis‐[3]ferrocenophanes **8** and **9** (R=SiMe_3_ (**13 a, 14 a, 8**), CH_2_ *t*Bu (**13 b, 14 b, 9**).

Nitrogen‐containing bis‐ferrocenophanes are accessible starting from 1,1′‐diaminoferrocenes. Base‐promoted condensation of **13 a** with PCl_3_ followed by reductive coupling of the spectroscopically detectable 2‐chloro‐1,3,2‐diazaphospha‐[3]ferrocenophane **14 a** 25 with magnesium yields tetraaza‐bis[3]ferrocenophane **8**. Finally, diaza‐bis‐[3]ferrocenophane **9**, which can be regarded as a link between **4** and **8**, is obtained via salt metathesis between **14 b** 25 and **3** (Scheme 3). Both **8** and **9** were isolated as yellow solids in reasonable to good yields.

**Figure 6:**
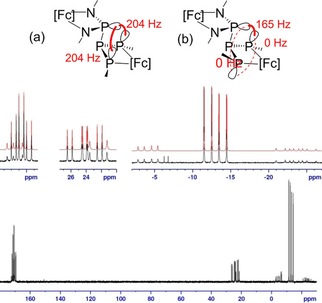
Bottom: ^31^P{^1^H} NMR spectrum of **9** with enlarged inserts showing expansions of the individual multiplets. Red traces represent the result of a spectral simulation as superposition of two diastereomers. Top: Schematic representation of the diastereomers of **9** with (a) *cis*‐ and (b) *trans*‐aligned *t*Bu‐substituents in the PPP‐ferrocenophane ring. Strong and weak through‐space interactions between remote lone‐pairs (as bold and dashed red lines, respectively) and magnitudes of ^2^ *J*_PP_ couplings are drawn in red colour ([Fc]=1,1’ ferrocenediyl).

The ^31^P NMR spectrum of **8** displays the single line of an A_2_ spin system. The ^31^P NMR spectrum of **9** displays two diastereomers featuring A_2_BX (major isomer) and ABMX (minor isomer) type spin systems (Figure 6). The ^1^H NMR spectrum appeared at first glance rather complicated, but the signals of the major component were readily identified and assigned from two‐dimensional ^1^H COSY and NOESY NMR spectra. Tracking characteristic NOESY correlation signals arising from mutual chemical exchange between both species then allowed us to assign the ^1^H NMR signals of the minor component, and to establish that both species undergo reversible dynamic interconversion on a second time scale. The number of ^1^H and ^31^P NMR signals and the coupling pattern in the ^31^P NMR spectrum exposes both components as isomers that are distinguished by different effective symmetry (*C*_2_ or *C*_s_ for the major and *C*_1_ for the minor isomer). These findings cannot be explained by assuming simply the presence of different rotamers as in **4**, **6**, and **7**, but are compatible with an assignment to two diastereomeric bis‐ferrocenophanes featuring *cis*‐(major isomer) and *trans*‐alignment (minor isomer) of the *t*Bu‐groups in the PPP‐ferrocenophane ring, respectively. To explain the observed patterns of ^2^ *J*_PP_ coupling constants, we hypothesize that the major isomer adopts a conformation that is characterized by a close proximity between the lone‐pairs on all three terminal phosphorus atoms which could provide substantial through‐space contributions23b–23d to the coupling between the diamino‐ and both *t*Bu‐substituted phosphorus atoms (Figure 5a). The *trans*‐isomer can formally be generated by inversion of one *t*BuP‐unit, which would quench any through‐space contributions to ^2^ *J*_PP_ couplings involving this phosphorus atom (Figure 5b). Similar conformational influences on *J*_PP_ coupling constants are well established in the literature.^21^, 23b–23d DFT calculations predict that the *cis*‐isomer is more stable, but the energy difference between the stereoisomers is small (7.5 kJ/mol). The ωB97XD/6‐31G* inversion barrier for isomerization of the *cis*‐ into the *trans*‐isomer is 66.9 kJ/mol, which is compatible with a reversible interconversion between both stereoisomers at ambient temperature. The inversion barriers of the phosphorus atoms in **9** are low within the P_3_ unit (66.9, 69.5 and 85.4 kJ/mol), unlike the inversion of the phosphorus atom in the NPN unit (146.0 kJ/mol).

**Figure 7:**
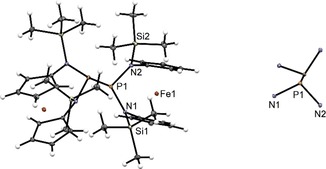
Molecular structure (left) of **8** and conformation of its N_2_P‐PN_2_ motif (right). Thermal ellipsoids for the heavy atoms are drawn at 30 % probability level.

**Figure 8:**
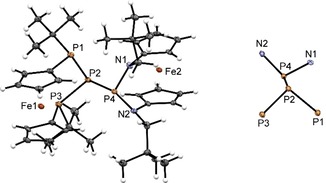
Molecular structure (left) of **9** and conformation of its N_2_P‐P_3_ motif (right). Thermal ellipsoids for the heavy atoms are drawn at 30 % probability level.

Single crystal XRD studies of **8** and **9** show a twisted arrangement with respect to the P−P bond for **8**, while **9** adopts a nearly antiperiplanar orientation (Figures 7 and 8). The P–P distance of the central bond is 2.3019(11) Å for **8** and 2.281(3) Å for **9**, both exceeding the respective bond length in Hittorf's phosphorus^7^, ^8^ but well within the range observed for bulkily substituted tetraamino‐diphosphanes (2.24–2.34 Å)^26^. The pyramidal coordination geometry around the phosphorus atoms of **8** (sum of bond angles 313(1)°) is slightly flattened compared to **4** (sum of bond angles 304–308°) and becomes even more planarized in **9** (sum of angles 322(1)°/323(1)°). The sums of bond angles at the nitrogen atoms indicate a quasi‐planar geometry for these atoms in both **8** (357(1)°/359(1)°) and **9** (352(2)°/347(2)°).

The observed conformation of crystalline **9** suggests that all phosphorus lone‐pairs in the P_3_ motif are oriented *gauche* to each other. The presence of short P–P distances (2.204(3)/2.212(3) Å) and *gauche*‐orientation of the lone‐pairs on adjacent phosphorus atoms in the PPP‐ferrocenophane ring compared to an increased P−P distance and an anti‐periplanar orientation of the lone‐pairs in the central bond are suitable to explain the large deviation between ^1^ *J*_PP_ coupling constants in the *ansa*‐ring (360–464 Hz) and the central bond (241 Hz), respectively. If **4** can serve as model compound for the connecting units in Hittorf's phosphorus, then **9** shows a similar connection mode as in fibrous red phosphorus (Ruck's Phosphorus).2a, 8a

### 2.5 Thermal Homolytic Bond Activation of bis‐[3]Ferrocenophanes

Thermally induced homolytic cleavage of the central bond in sterically encumbered diphosphanes (X_2_P−PX_2_) is an attractive and traceless way to generate phosphorus centred radicals. This methodology works particularly well for the generation of amino‐phosphanyl radicals (X_2_P^.^; X=R_2_N) which entails a propensity for bond fragmentation in sterically strained tetraaminodiphosphanes.17b, 26, 28 In this context, we explored the potential of the above mentioned bis‐[3]ferrocenophanes to undergo thermally induced homolytic cleavage of the central P−P, P−Si or Si−Si bond, respectively.

**Figure 9:**
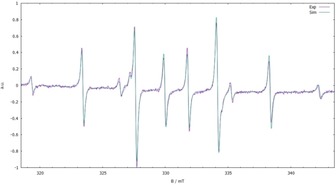
Measured EPR spectrum (violet trace) of a solution of **4** at 150 °C in mesitylene and result of a spectral simulation (green trace).

The experimental studies were carried out by heating solutions of bis‐ferrocenophanes **4**, **6–9** in suitable inert solvents (toluene, mesitylene) in the cavity of an EPR spectrometer in order to detect any radicals resulting from a homolytic bond fission process. The temperature was generally raised in 10 °C steps up to a maximum of 165 °C. In case of **4**, visible EPR signals started to emerge at approximately 90 °C and grew continuously in intensity until the maximum temperature of 165 °C was reached. The temperature dependent changes were reversed when the temperature was lowered again, and the signals disappeared again below 90 °C. The observed spectral pattern (Figure 9) can be decomposed into two signals with relative intensities of approx. 1 : 3 that do not vary perceptibly over the temperature range studied.

**Scheme 4:**
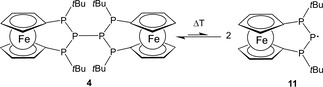
P−P bond homolysis of **4**.

Spectral simulations allowed to describe each signal as a doublet of triplets resulting from hyperfine coupling with one and two phosphorus nuclei, respectively (more intense signal: *g*=2.0012, *A*=40.1 G (1 P), 65.4 G (2 P); less intense signal: g=2.0011, *A*=70.6 G (1 P), 79.5 G (2 P)). The hyperfine splitting pattern of both signals is in accord with the presence of triphospha‐[3]ferrocenophanyl radicals **11** (Scheme 4), the detection of which indicates that the expected bond homolysis process has taken place. The observation of more than one signal can be accounted for by the presence of different stereoisomers. Anticipating that the CP_3_C‐bridge (where C denote the P‐substituted Cp‐carbon atoms) adopts a similar non‐planar conformation as in **4**, one can envisage two diastereomers featuring *cis‐* or *trans*‐orientation of the P‐*t*Bu‐substituents relative to the “ring” structure defined by the two Cp‐centroids and the P_3_ bridge, respectively. DFT calculations support the identity of radical **11**. The calculated (ωB97XD/6‐31+G*) Gibbs free energy difference between the two isomers is only 0.8 kJ/mol, and the barrier for planar inversion at one of the phosphorus atoms is only 65.7 kJ/mol). The calculated spin density (Figure S1) verifies that the radical center is localized at the central phosphorus atoms of both isomers. The calculated hyperfine coupling constants (for more information see Table S2 in SI) are in agreement with the experimentally observed values and give further support for the proposed constitutional assignments of the isomeric species. Similar conformational isomerization processes as observed here have been reported in related cases.11d

The observed temperature dependence of the EPR spectra suggests that the radicals **11** recombine at lower temperature and exist in a dynamic equilibrium with the diphosphane. Similar reversible equilibria have precedence for amino‐diphosphanes.17b, 26, 28a–28f Prolonged heating of toluene solutions of **4** under inert conditions affords detectable amounts of secondary phosphane **2**, suggesting that radicals **11** may be irreversibly quenched by hydrogen abstraction, e. g. from the solvent. Triphosphane **2** is also formed in the reaction of **4** with tributyltin hydride. Even if the tin‐containing reaction product could not be identified, formation of the phosphane can be accounted for by a radical promoted reductive hydrogenation of the central P−P bond in **4**.

**Scheme 5:**
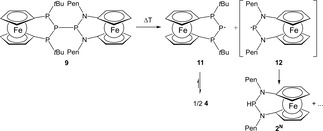
P−P bond homolysis of **9**.

HT EPR measurements on toluene solutions of **9** revealed that the signals attributable to radical **11** already appeared at ca. 60 °C. Interestingly, signals attributable to the complementary NPN radical **12** could not be detected. The EPR studies suggest that homolytic cleavage of the central P−P bond in **9** occurs even more readily than in case of **4** but the postulated aminophosphanyl radical is immediately quenched (Scheme 5).

This hypothesis is corroborated by the observation that heating of a toluene solution of **9** to 100 °C under inert conditions results in controlled decomposition, which can be traced by ^31^P NMR spectroscopy. With continuous heating, the signals of **9** fade away, while those of recombination product **4** and hydrogen abstraction product **2** together with two additional singlets at lower field start to grow in. One of the new signals at 110.4 ppm splits into a doublet with a *J*_PH_ coupling constant of 272 Hz when proton decoupling is switched off. Similar shifts and coupling constants were reported for secondary diaminophosphanes,^29^ and we assign this signal therefore to the NPN analogue **2^N^** of **2**. The formation of **2^N^** is in good agreement with the predicted exothermic outcome (ΔE=−23.4 kJ/mol at the ωB97XD/6‐31+G* level) of the reaction **2**+**12**→**2^N^** +**11**, which implies that the hydrogen abstraction capability of NPN‐based radical **12** is higher than that of PPP‐based radical **11**. The chemical shift of the second singlet at 165.0 ppm is slightly larger than those of bulkily substituted N‐heterocyclic diphosphanes10b, 21 which resonate around 150 ppm. It is tempting to attribute the singlet to a symmetrical diphosphane resulting from recombination of radical **12**. However, all attempts to access **2^N^** or the postulated symmetrical diphosphane by reduction of chlorophosphane **14 b**, using e. g. lithium aluminium hydride or metal mediated reductive coupling reactions, were as yet unsuccessful.

Similar HT EPR experiments on tetraamino‐bis‐ferrocenophane **8** and the silicon analogues **6** and **7** up to 120 resp. 250 °C do not result in formation of any detectable radicals. While the absence of the dissociation in case of **6** and **7** is in good agreement with their higher dissociation Gibbs free energy (175.7 kJ/mol for **6** and 225.1 kJ/mol for **7** at ωB97XD/6‐31+G* level of theory and related to 25 °C) compared to **4** and **9** (93.3 kJ/mol and 100.8 kJ/mol, respectively), the low calculated dissociation Gibbs free energy of 3.8 kJ/mol for **8** suggests that thermolytic cleavage should be energetically feasible. However, quenching and high reactivity of the N_2_P centered radical may preclude its practical observation just as in the case of **9**.

#### 2.5.1 Electrochemical Behavior of Bis‐[3]ferrocenophanes

Intrigued by the energetic proximity of iron and phosphorus centered electronic states in the frontier orbital region of such compounds, we explored the electrochemical properties of the bis‐[3]ferrocenophanes presented. Cyclic voltammetry measurements of **4** in THF with a platinum working electrode in the presence of TBAHFP as conducting salt revealed an irreversible oxidation process with its peak potential at 0.03(1) V (vs. Fc/Fc^+^). Several further oxidations indicated by broad current responses can be observed at potentials around 1 V (vs. Fc/Fc^+^). After several cycles, a second defined oxidation wave starts to emerge at ca. 0.2 V (Figure S4). We assign this wave to triphosphane **2**, which shows an oxidation response of the ferrocene unit at 0.22(1) V (vs. Fc/Fc^+^) in DCM (see SI for detailed information). This interpretation is consistent with the observation of detectable amounts of **2** in ^31^P NMR spectra recorded subsequent to the electrochemical experiments and implies that electrochemical oxidation of **4** entails formation of **2**, probably via intermediate formation of radical **11** which should be prone to hydrogen abstraction from the solvent (see above). The dissociation of **4** ^.**+**^ into **11** and the corresponding cation is somewhat endergonic (the Gibbs free energy is 36.0 kJ/mol at ωB97XD/6‐31+G*), which is significantly smaller than the 93.3 kJ/mol required for the dissociation of neutral **4** into two molecules of **11**. For further elucidation, we performed dual electrode experiments on **4** using a rotating ring‐disc electrode to determine the average lifetime of **4** ^.**+**^. However, even at rotation rates up to 2000 RPM no reductive response could be detected at the ring, indicating a lifetime of the anticipated radical cation shorter than 0.02 seconds (Figure S6).

Hybrid structure **9** displays similar electrochemical behavior, although more redox events are detectable. The first oxidation occurs at a potential of −0.26(1) V (vs. Fc/Fc^+^), which is in the expected range for amino‐substituted ferrocenophanes,^25^ as an irreversible process (Figure S7). At higher potential, two quasi reversible oxidation processes (0.02(1) and 0.13 (1) V vs. Fc/Fc^+^) were detected using differential pulse techniques. Further broad current responses above 0.4 V (vs. Fc/Fc^+^) indicate consecutive reactions which are not interpretable. Both current responses at potentials near the ferrocene standard are comparable to hexaphosphane **4**. As in the case of **4**, repetitive measurements entail a distinct decrease of the signals accompanied by new signals at −0.66(1) V vs. Fc/Fc^+^ (for comparison 1,1’‐diaminoferrocene at −0.60 V^30^ resp. 1,1’‐bis(dimethylamino)ferrocene at −0.63 V^31^ vs. Fc/Fc^+^ in CH_3_CN) and around 0.1‐0.2 V. The latter is superimposed by the previously mentioned broad signals, but could be attributed to triphosphane **2**. In accordance, ^31^P NMR experiments on the electrolyte solution after the measurements revealed the presence of triphosphane **2** and evidence for fluoro‐analogue **14b^F^**. It is noteworthy that measurements with chloride‐containing conducting salts like tetrabutylammonium chloride were precluded by instantaneous reaction with compound **9** forming chlorophosphane **14 b**. Both experimental findings imply a cleavage of the central P−P bond upon oxidation or by reaction with the conducting salt, which might well be heterolytic with the positive charge remaining on the N_2_P scaffold. Analogously to the case of **4**, oxidation of **9** to **9^•^** ^+^ can be considered to permit facile dissociation of the central P−P bond – the Gibbs free energy for the decomposition of **9** ^.+^ to **11** and **12^+^** was calculated as 61.1 kJ/mol (the alternative fragmentation of **9** ^.**+**^ into **11^+^** and **12** was calculated as more endergonic by 86.6 kJ/mol) – which suggests to relate the electrochemical behavior to the occurrence of chemical decay processes (see Figure S2 for spin density distribution in **4** ^.+^ and **9** ^.+^).

Similar to **4** and **9**, diphosphane **8** reveals an irreversible first oxidation process (−0.21(1) V vs. Fc/Fc^+^; Figure S8). A second process follows at 0.11(1) V (vs. Fc/Fc^+^), which appears as (quasi‐)reversible oxidation at first glance. Again, broad current responses are detectable at higher potentials which cannot be assigned. Differential pulse measurements indicate that the reductive response is independent from the second oxidation wave, which may be attributed to the occurrence of unspecified follow‐up products. In case of the silicon based bis‐[3]ferrocenophanes **6** and **7**, unspecific fragmentation of the main‐group framework occurred, which precluded further interpretation. As a general feature of these compounds, their propensity to undergo inversion and other dynamic processes entails a variable energetic sequence of the frontier orbitals, which precludes a simple correlation between the electrochemical behavior and the HOMO/LUMO orbitals of the ground state.

## 3 Conclusions

In summary, we developed a series of bis‐[3]ferrocenophanes in which the *ansa*‐units consist of isolobal trivalent fragments. Despite the number of potential P‐stereogenic centers, the ferrocenophane motif limits the number of diastereomers observed for this series of compounds. As a characteristic feature, most compounds show restricted rotation around the central bond at ambient temperature, which leads to a characteristic temperature dependence of the respective NMR spectra. Quite remarkably, DFT calculations predict that the inversion barriers for some bridgehead phosphorus atoms are only slightly higher than the activation energies for the rotational processes. For bis‐ferrocenophane **4**, thermally induced homolytic cleavage of the central P−P bond of the P_2_P‐PP_2_ fragment is observed at elevated temperature. Similarly electrochemical oxidation leads to the hydrogenated derivatives of the same radical. The barrier for thermal P−P fission is significantly reduced by formal change to a P_2_P‐PN_2_ as in **9** (and N_2_P‐PN_2_ as in **8**). Formal replacement of P with isolobal SiH units on the other hand does not lead to analogous silyl radicals up to a temperature of 250 °C. Based on the structural analogy of **4** with P_2_P‐PP_2_ fragments in phosphorus modifications outlined above, our results impose the question whether similar homolytic bond rupture may occur in such solid state materials at elevated temperatures as well, although the dissociation barriers are to be expected at much higher energies owing to the reinforcement of the columnar structures in the lattice.

## Conflict of interest

The authors declare no conflict of interest.

## Supporting information

As a service to our authors and readers, this journal provides supporting information supplied by the authors. Such materials are peer reviewed and may be re‐organized for online delivery, but are not copy‐edited or typeset. Technical support issues arising from supporting information (other than missing files) should be addressed to the authors.

Supplementary
