# Supplementary material for: Bis‐[3]Ferrocenophanes with Central >E−E’< Bonds (E, E’=P, SiH): Preparation, Properties, and Thermal Activation
Source: ChemistryOpen. 2019 Jun 26;8(10):1235–43. doi: 10.1002/open.201900182 (PMC6769432; doi:10.1002/open.201900182)
Supplement: Supplementary file 1 — Supplementary [file OPEN-8-1235-s001.pdf]

Open Access
ChemistryOPEN

Supporting Information

© Copyright Wiley-VCH Verlag GmbH & Co. KGaA, 69451 Weinheim, 2019

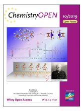

Bis-[3]Ferrocenophanes with Central > E–E' < Bonds (E, E'=P, SiH): Preparation, Properties, and Thermal Activation

Stefan Isenberg, Stefan Weller, Denis Kargin, Srećko Valić, Brigitte Schwederski, Zsolt Kelemen, Clemens Bruhn, Kristijan Krekić, Martin Maurer, Christoph M. Feil, Martin Nieger, Dietrich Gudat,* László Nyulászi,* and Rudolf Pietschnig*© 2019 The Authors. Published by Wiley-VCH Verlag GmbH & Co. KGaA. This is an open access article under the terms of the Creative Commons Attribution License, which permits use, distribution and reproduction in any medium, provided the original work is properly cited.

Content:

a) Synthesis
b) Computational details
c) Cyclic Voltammetry
d) X-ray Crystallography
e) EPR measurements
f) NMR Spectra
g) XYZ coordinates and total energies of computed molecules

Experimental Details

General procedures

All experiments were carried out under exclusion of moisture and air under an inert argon atmosphere. All solvents were dried over sodium potassium alloy and distilled prior to use. Starting materials were purified and stored under argon. Fc'(P*t*Bu)$_2$PCl **1**, Fc'(P*t*Bu)$_2$PH **2** and Fc'(P*t*Bu)$_2$SiHCl **5** were synthesized according published procedures.[3b] ^{1}H-, ^{13}C-, ^{29}Si- and ^{31}P-NMR spectra were recorded on Varian VNMRS-500 MHz or MR-400 MHz, or Bruker AV400 or AV250 spectrometers using TMS as the external reference for all nuclei and 85% H_3PO_4 (Ξ = 40.480747 MHz) as secondary reference for ^{31}P. Spectra were recorded at ambient temperature unless mentioned otherwise. For compounds **6** and **9**, ^{13}C-^{1}H correlation experiments were used for detailed chemical shift assignments. The signals of the two stereoisomers of complex **9** were assigned based on the evaluation of 2D NMR spectra (^{1}H COSY, ^{1}H NOESY, ^{1}H,^{13}C HSQC and HMBC). The relative isomer ratio was determined from the simulation of the ^{31}P NMR spectrum. The ^{13}C NMR signals of the minor isomer could not be unambiguously assigned and are omitted. Simulations of NMR spectra were carried out by iterative fitting procedures with the DAISY (static spectra) and DNMR (dynamic spectra) modules implemented in the TOPSPIN software. Results are displayed in Figure 2 and in the supporting information. Chemical shifts and coupling constants derived from spectra measured at low temperature are included in the experimental sections and rate constants from dynamic spectra in the supporting information. Coupling constants are given as absolute values except for compound **4** where $^{1}J_{PP}$ coupling constants were assumed to be negative and the signs of the remaining coupling constants were determined during the least-squares fit. Even for crystalline samples, microanalyses gave in most cases too low carbon values, suggesting metal carbide formation which could not be suppressed using standard additives such as V_2O_5. All compounds were characterized via APCI-MS spectra recorded on a ThermoQuest Finnigan LCQ Deca.

Electron paramagnetic resonance (EPR) measurements were performed on Bruker EMX or Varian E-109 spectrometers, the latter equipped with a Bruker ER 041 XG microwave bridge and a Bruker ER 4111 VT temperature unit. EW (EPRWare) Scientific Software Service program was used for data accumulation and manipulation on the E-109 spectrometer. Hyperfine splittings were determined by spectral simulation with the program EasySpin.[33]

*Synthesis of **4***

To a stirred solution of 0.6 g (1.5 mmol) **2** and 0.25 mL (1.6 mmol) TMEDA in 40 mL pentane 0.6 mL (1.5 mmol, 2.5 M) *n*BuLi were added dropwise at room temperature. After continued stirring for 30 minutes 0.64 g (1.5 mmol) of **1** dissolved in 20 mL pentane were added and the mixture was stirred for another 30 minutes. The precipitate was separated and washed with 20 mL pentane before it was extracted with 10 mL toluene. The white precipitate was separated again and all volatiles have been removed under reduced pressure. The product was obtained as yellow-orange solid. Recrystallisation from toluene at -20 °C afforded crystals suitable for X-ray diffraction analysis. Yield: 1,0 g (85%).

^{1}H-NMR (400 MHz, C_6D_6, rt): δ 1,64 (d, 36H, $^{3}J_{HP}$ = 11.3 Hz, *t*Bu CH_3), 3.96 (m, 4H, Cp), 4.07 (m, 4H, Cp), 4.41 (m, 4H, Cp), 4.48 (m, 4H, Cp) ppm. ^{13}C-NMR (100 MHz, C_6D_6, rt): δ 32.0 (m, 12C, *t*Bu CH_3), 34.4 (m, 4C, *t*Bu C_q), 71.3 (m, 4C, Cp), 72.3 (s, 4C, Cp), 72.7 (m, 4C, Cp), 75.4 (m, 4C, Cp C_{ipso}), 78.6 (m, 4C, Cp) ppm. ^{31}P{^{1}H} NMR (202 MHz, C_7D_8, -50 °C): simulated as AA'MM'XX' spin system with $\delta_{A,A'}$ = 18.8 (P*t*Bu), $\delta_{M,M'}$ = -4.0 (P*t*Bu), $\delta_{X,X'}$ = -59.51 (>P-) ppm, $^{2}J_{AM}$ = 167.2 Hz, $^{1}J_{AX}$ = -213 Hz, $^{1}J_{MX}$ = -150 Hz, $^{2}J_{MX'}$ = 59 Hz, $^{1}J_{XX'}$ = -357 Hz, $^{3}J_{MM'}$ = -5 Hz, $^{3}J_{XX'}$ = -9 Hz. MS (APCI-HR) *m/z*: 361.102247 (100%, [Fc'(P*t*Bu)$_2$ + H]$^+$), 783.127446 (3%, [M+H]$^+$, calc. 783.126631).

*Synthesis of **6***

To a mixture of 55 mg (0.14 mmol) **2** and 16 mg (0.14 mmol) KO*t*Bu 2 mL THF was added under stirring at room temperature. After 10 minutes all volatiles have been removed under reduced pressure and the residue was dissolved in 2 mL THF. To this red solution 60 mg (0.14 mmol) of **5** in 2 mL THF were added and the mixture was stirred for 20 h. The solvent has been removed *in vacuo*, the residue washed twice with 10 mL pentane and extracted with 5 mL toluene. Removing the solvent under reduced

pressure afforded the product as yellow solid. Crystals suitable for x-ray diffraction analysis could be obtained by recrystallisation from toluene. Yield: 33 mg (30%).

^{1}H-NMR (500 MHz, toluene-d_8, rt): δ 1,51 (*br* d, 18H, $^3J_{HP}$ = 10.7 Hz, SiP*t*Bu CH_3), 1.58 (d, 18H, $^3J_{HP}$ = 12.6 Hz, PP*t*Bu CH_3), 3.94 (m, 2H, SiPCp), 3.99 (m, 2H, PPCp), 4.07 (m, 2H, SiPCp), 4.08 (m, 2H, PPCp), 4.23 (m, 2H, PPCp), 4.34 (m, 2H, SiPCp), 4.38 (m, 2H, PPCp), 4.48 (m, 2H, SiPCp), 5.82 (dt, 1H, $^2J_{HP}$ = 13.8 Hz, $^2J_{HP}$ = 6.7 Hz, SiH) ppm. ^{13}C-NMR (126 MHz, toluene-d_8, rt): δ 32.0 (m, 6C, SiP*t*Bu CH_3), 32.4-32.6 (m, 2C, PP*t*Bu C_q), 32.6-32.9 (m, 6C, PP*t*Bu CH_3), 34.5 (m, 2C, SiP*t*Bu C_q), 70.9 (m, 2C, SiPCp), 71.3 (m, 2C, PPCp), 72.0 (m, 2C, SiPCp), 71.9-72.1 (m, 2C, PPCp C_q), 72.2 (m, 2C, Cp), 72.3 (m, 2C, Cp), 73.9 (m, 2C, PPCp), 77.0-77.6 (m, 2C, SiPCp C_q), 79.0 (m, 2C, SiPCp), 79.4 (m, 2C, PPCp) ppm. ^{29}Si-NMR (INEPT, 99 MHz, toluene-d_8, rt): δ -28.8 - -25.7 (m, 1Si, SiH) ppm. ^{31}P{^{1}H}-NMR (202 MHz, toluene-d_8, -40 °C): simulated as AFHMX spin system with δ_A= 14.0 (P-**P***t*Bu) ppm, δ_F = 4.3 (P-**P***t*Bu), δ_H = -40.3 (Si-P*t*Bu), δ_M= -57.3 (Si-P*t*Bu), δ_X = -109.1 (>PSi(H)<), $^2J_{AF}$ = 139 Hz, $^1J_{AX}$ = 165 Hz, $^1J_{FX}$ = 153 Hz, $^2J_{HM}$ = 113 Hz, $^2J_{MX}$ = 32 Hz. MS (APCI-HR) *m/z*: 781.137768 (100%, $[M+H]^+$, calc. 781.137621).

*Synthesis of **7***

100 mg (0.24 mmol) of **5** was mixed with 15 mg (2.17 mmol) of lithium ribbon and suspended in 2.5 mL toluene. Stirring at elevated temperatures (170 °C oil bath temperature) in a closed vessel resulted in the formation of a colorless precipitate. All volatile compounds were removed under reduced pressure and the remaining residue was washed three times with 20 mL pentane, which was discarded. The solid residue was extracted twice with 20 mL toluene and the solvent was evaporated *in vacuo* to give a yellow solid. Crystalline material suitable for X-ray diffraction analysis could be obtained by recrystallization from boiling toluene. Yield: 45 mg (49% yield).

^{1}H-NMR (500 MHz, $CDCl_3$, rt): δ 1.12-1.47 (m, 36H, *t*Bu CH_3), 4.26 (m, 4H, Cp), 4.30 (m, 4H, Cp), 4.39 (m, 4H, Cp), 4.45 (m, 2H, Cp), 4.52 (m, 2H, Cp), 5.15 (*br* d, 2H, $^1J_{HSi}$ = 181 Hz, $^2J_{HP}$ = 6.3 Hz, SiH) ppm. ^{13}C-NMR (126 MHz, $CDCl_3$, rt): δ 31.5-31.8 (m, 2C, *t*Bu C_q), 31.9-32.0 (m, 6C, *t*Bu CH_3), 32.0-32.3 (m, 2C, *t*Bu C_q), 32.6-33.3 (m, 6C, *t*Bu CH_3), 70.5 (m, 4C, Cp), 72.0 (m, 4C, Cp), 72.2 (m, 2C, Cp C_{ipso}), 73.1 (m, 2C, Cp), 73.9 (m, 2C, Cp C_{ipso}), 74.0 (m, 2C, Cp), 79.5 (m, 4C, Cp) ppm. ^{29}Si-NMR (99 MHz, $CDCl_3$, rt): δ -49.5 - -45.7 (m, 2Si, SiH) ppm. ^{31}P{^{1}H}-NMR (202 MHz, $CDCl_3$, rt): δ -50.1 (d, 2P, $^2J_{PP}$ = 123.4 Hz, P*t*Bu), -44.9 (d, 2P, $^2J_{PP}$ = 123.4 Hz, P*t*Bu) ppm. MS (APCI-HR) *m/z*: 779.1488 (100%, $[M+H]^+$, calc. 779.1486). ATR-IR: ν(SiH) = 2080 cm^{-1}. EA: Anal. calcd. for $C_{36}H_{54}Fe_2P_4Si_2$ (778.6): C 55.54 H 6.99; found: C 55.25 H 6.91.

*Synthesis of **8***

PCl_3 (0.08 ml, 0.93 mmol) was added dropwise to a stirred and cooled (-78 °C) solution of 1,1'-bis(trimethylsilyl)aminoferrocene (0.34 g, 0.94 mmol) and triethyl amine (0.35 ml, 2.5 mmol) in Et_2O (20 ml). The solution was stirred for one additional hour at -78 °C after the addition was complete, and then allowed to warm up to room temperature and stirred overnight. Precipitated solids were removed by filtration and magnesium shavings (0.50 g, 20 mmol) followed by two crystals of iodine were added. The mixture was stirred for 12h, filtrated, and the filtrate evaporated to dryness under reduced pressure. The remaining residue was treated with pentane (10 ml) and the resulting suspension filtrated one more time. Storing the resulting clear yellow solution at -28°C afforded crystals of "**8**" that were suitable for X-ray diffraction analysis (yield 10 mg, 13%, m.p. (dec.) 154 °C).

^{1}H-NMR (C_6D_6) δ: 4.70 (m, 4 H, C_5H_4), 4.15 (m, 4 H, C_5H_4), 3.99 (m, 4 H, C_5H_4), 3.85 (m, 4 H, C_5H_4), 0.31 (m, 36 H, ΣJ_{HP} = 1.4 Hz, $Si(CH_3)_3$). ^{13}C{^{1}H}-NMR (C_6D_6) δ: 69.2 (s, C_5H_4), 67.6 (s, C_5H_4), 66.4 (s, C_5H_4), 63.8 (s, C_5H_4), 1.5 (t, 4.3 Hz, $Si(CH_3)_3$). ^{31}P{^{1}H}-NMR (C_6D_6) δ: 133.2 (s). EA: Anal. calcd. for $C_{32}H_{52}Fe_2N_4P_2Si_4$ (778.8): C 49.35 H 6.73 N 7.19; found: C 48.92 H 6.60 N, 7.10.

*Synthesis of **9***

The synthesis followed the identical route like compound **4**. Instead of **1** one equivalent of the analogous N_2PCl compound **10** (0.21 g, 0.5 mmol) was used. The product was obtained as orange solid.

Recrystallisation from toluene afforded crystals suitable for x-ray diffraction analysis. Yield: 303 mg (78%).

Isomer 1 (approx. 77%): ^{1}H NMR (500 MHz, C_6D_6): δ 1.18 (s, 18 H, Np-CH_3), 1.45 (m, 18 H, *t*Bu-CH_3), 3.48 (m, 2 H, CH_2), 3.62 (dd, $^{2}J_{HH}$ = 14.5 Hz, $^{3}J_{HP}$ = 14.5 Hz, 2 H, CH_2), 3.87 (s, 2 H, N-Cp), 4.11 (s, 4 H, P-Cp), 4.19 (s, 2 H, N-Cp), 4.22 (s, 2 H, N-Cp), 4.30 (s, 2 H, P-Cp), 4.64 (s, 2 H, PCp), 4.96 (s, 2 H, N-Cp) ppm. ^{13}C{^{1}H} NMR (125.7 MHz, C_6D_6): δ 29.8 (s, Np-CH_3), 31.7 (m, *t*Bu-CH_3), 32.4 (m, *t*Bu-C), 35.7 (d, $^{3}J_{CP}$ = 10 Hz, Np-C_q), 63.2 (d, $^{3}J_{CP}$ = 15 Hz, N-Cp), 64.3 (s, N-Cp), 68.3 (s, N-Cp), 68.6 (s, N-Cp), 69.6 (d, $^{2}J_{CP}$ = 42 Hz, CH_2), 70.2 (s, P-Cp), 70.8 (m, $\bar{J}$ = 5 Hz, P-Cp), 75.3 (s, P-Cp), 75.5 (dm, $^{3}J_{CP}$ = 11 Hz, $\bar{J}$ = 21 Hz, P-Cp), 86.6 (m, P-Cp_{ipso}), 106.7 (d, $^{2}J_{CP}$ = 11 Hz, N-Cp_{ipso}) ppm. ^{31}P{^{1}H} NMR (202 MHz, C_6D_6): δ -13.0 (m, 2 P, $^{1}J_{PP}$ = 398 Hz, $^{2}J_{PP}$ = 204 Hz, P*t*Bu), 24.1 (m, 1 P, $^{1}J_{PP}$ = 398 Hz, $^{1}J_{PP}$ = 130 Hz, P_2*P*), 170.7 (dt, 1 P, $^{1}J_{PP}$ = 130 Hz, $^{2}J_{PP}$ = 204 Hz, N_2P) ppm. – Isomer 2 (approx. 23%): ^{1}H NMR (500 MHz, C_6D_6): δ 1.07 (br, 18 H, Np-CH_3), 1.51 (br, 18 H, *t*Bu-CH_3), 3.37 (br, 1 H, CH_2), 3.52 (br, 2 H, CH_2), 3.78 (br, 1 H, CH_2), 3.87 (s, 2 H, N-Cp), 4.12 (br, 3 H, N-Cp), 4.19 (br, 2 H, P-Cp), 4.21 (br, 1 H, N-Cp), 4.28 (br, 2 H, P-Cp), 4.47 (s, 1 H, P-Cp), 4.74 (s, 1 H, P-Cp), 4.75 (br, 1 H, N-Cp), 4.81 (br, 1 H, P-Cp), 4.99 (br, 1 H, N-Cp), 5.29 (br, 1 H, P-Cp) ppm. ^{31}P{^{1}H} NMR (202 MHz, C_6D_6): δ -23.6 (dddd, 1 P, $^{1}J_{PP}$ = 464 Hz, 360 Hz, 241 Hz, P_2*P*), -4.2 (dd, 1 P, $^{1}J_{PP}$ = 360 Hz, $^{2}J_{PP}$ = 165 Hz, P*t*Bu), 22.4 (d, 1 P, $^{1}J_{PP}$ = 464 Hz, P*t*Bu), 171.6 (dd, 1 P, $^{1}J_{PP}$ = 241 Hz, $^{2}J_{PP}$ = 165 Hz, N_2P) ppm. MS (APCI-HR) *m/z*: 385.150469 (100%, [M – Fc'$(PtBu)_2$P]$^{+}$, calc. 385.14960), 393.076182 (84%, [Fc'$(PtBu)_2PH_2$]$^{+}$, calc. 393.07533).

Computational details

Quantum chemical calculations were performed using the Gaussian 09 program package.[34] Full geometry optimization was performed for all the molecules at the ωB97XD/6-31G* (investigation of inversion barriers) or ωB97XD/6-31+G* level of theory. The nature of stationary points was verified by a subsequent analysis of the second derivatives, which were found all positive in case of minima, and exhibited a single negative value in case of transition states. During the scan calculations, the stability of the wavefunction of the systems was carefully investigated. The MOLDEN 4.0 program[35] was used for the visualization of the results. The electron density in bond critical points was calculated with the Multiwfn program.[36]

	+0.0 Å	+0.2 Å	+0.3 Å	+0.5 Å
P_2Me_4	0.0	4.2	8.3	18.6
P_6H_8	0.0	3.9	7.8	17.3
**4**	0.0	3.7	6.7	14.9
**6**	0.0	-	-	17.7
**7**	0.0	-	-	17.3
**8**	0.0	-	-	17.2
**9**	0.0	-	-	13.9

Table S1 The energy level (in kcal/mol at ωB97XD/6-31G*) of the investigated systems after increasing the central bondlength by 0.2 Å, 0.3 Å and 0.5 Å

spinsystem		Exp.	B3LYP/cc-pVTZ// ωB97XD/6-31+G*	B3LYP/Def2TZVP// ωB97XD/6-31+G*
cis	center P	40.1	46.3	45.2
	outer P	65.4	53.9	60.8
trans	center P	70.6	55.2	61.9
	outer P (avarage)	79.5	82.9	80.7

Table S2. Calculated hyperfine coupling constants of the *cis* and *trans* isomer of **11** (in Gauss)

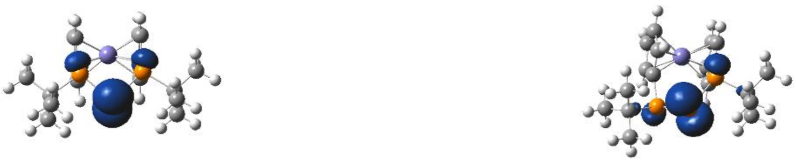

Figure S1. Spin density distribution of the *cis* (left) and *trans* isomer (right) of **11**.

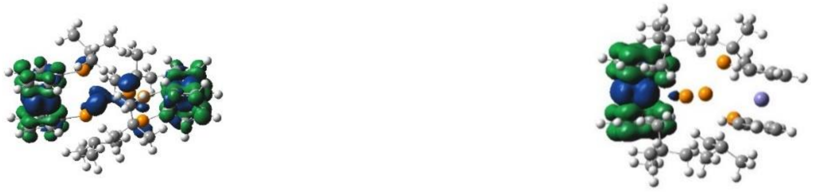

Figure S2. Spin density distribution of **4**$^{+}$ (left) and **9**$^{+}$ (right). While in case of **4**$^{+}$ The spin density mainly localized at the ferrocene units, the central P-P bond and the lonepairs of the outer P atoms has small contribution in case of **9**$^{+}$ the spin density mainly localized at one of the ferrocene unit.

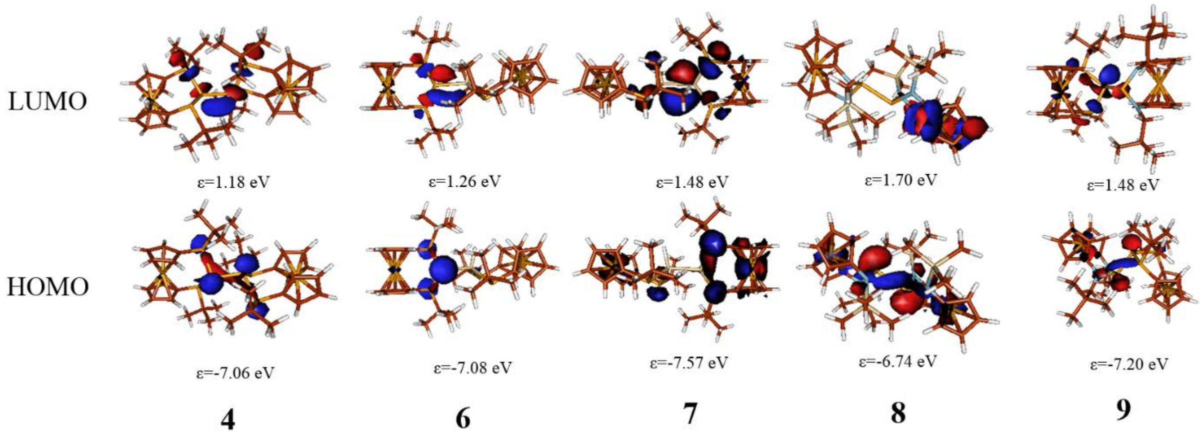

Figure S3. Kohn-Sham frontier orbitals (and their energies in eV) of the investigated bis-[3]ferrocenophanes

Electrochemical measurements

Cyclic voltammetric and differential pulse measurements were carried out in a MBraun acrylic glovebox GB2202-C-VAC under inert argon atmosphere. All samples were measured in anhydrous dichloromethane or tetrahydrofurane, which had been stored over molecular sieve (3 Å) under argon atmosphere. Tetrabutylammonium hexafluorophosphate ($[NBu_4]$ $[PF_6]$) or chloride ($[NBu_4]Cl$) served as conducting salt at a concentration of 0.1 mol/L. The sample concentration during the measurements was set to 0.1 mmol/L. The setup consisted of a three-electrode cell with a platinum disk as working electrode, a silver spiral as counter electrode and a silver pseudo reference electrode. While the potential was driven on the WaveDriver 20 Bipotentiostat from Pine Research Instrumentation, electrochemical data were recorded via AfterMath (Ver. 1.2.5966; Pine Instruments). The half wave

potentials of the redox processes were referenced using either ferrocene or decamethyl ferrocene and evaluated with OriginPro (Ver. 8.6.0; OriginLab Corporation).

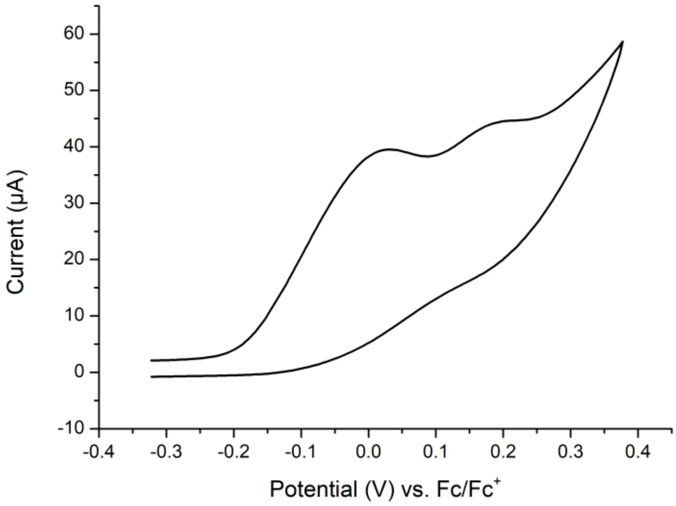

Figure S4: Cyclic voltammetry of **4** (referenced vs. Fc/Fc^{+}) after several cycles. Voltage sweep 250 mV/sec. The second current response (ca. 0.2 V) originates from triphosphane **2**, which probably forms via the intermediate radical **11**.

Cyclic voltammetry measurements of triphosphane **2** in DCM with platinum working electrode in the presence of TBAHFP as conducting salt show two distinct oxidation events with peak potentials at 0.22(1) V and ca. 1 V (vs. Fc/Fc^{+}; see black line Figure S5). Both current responses reveal irreversible redox behavior. Based on spindensity calculations (Figure S5), the first oxidation event can be assigned to oxidation of the ferrocene backbone. Turning of the potential sweep after the first oxidation chances the fate of oxidized $\mathbf{2}^{+}$ and revealing a reversible oxidation behavior by avoiding the oxidation of the phosphane scaffold (see red line Figure S5).

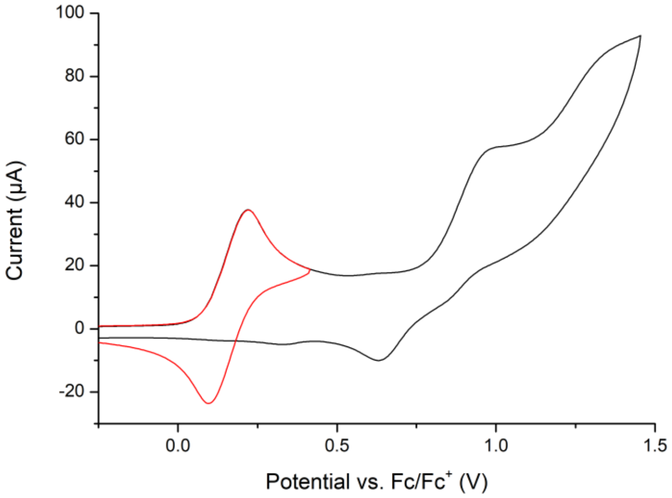

Figure S5: Cyclic voltammetry of **2** (referenced vs. Fc/Fc^{+}). Voltage sweep 250 mV/sec.

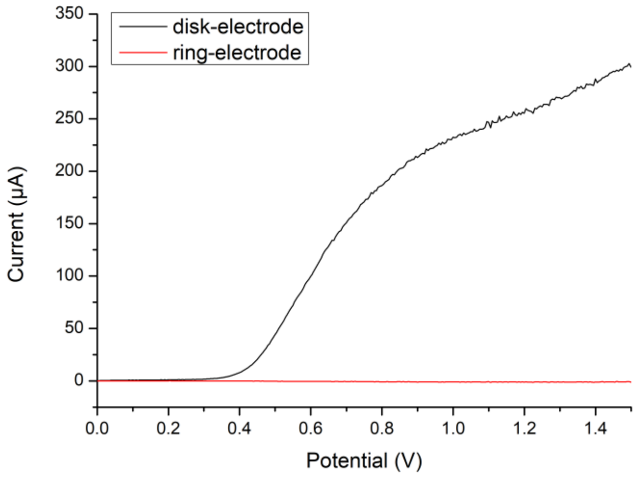

Figure S6: Dual electrode voltammetry of **4** at 2000 rpm. Voltage sweep 100 mV/sec.

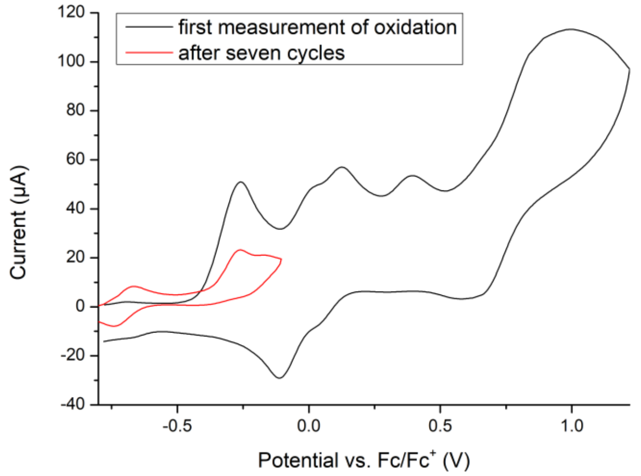

Figure S7: Cyclic voltammetry of **9** (referenced vs. Fc/Fc^{+}). Voltage sweep 250 mV/sec.

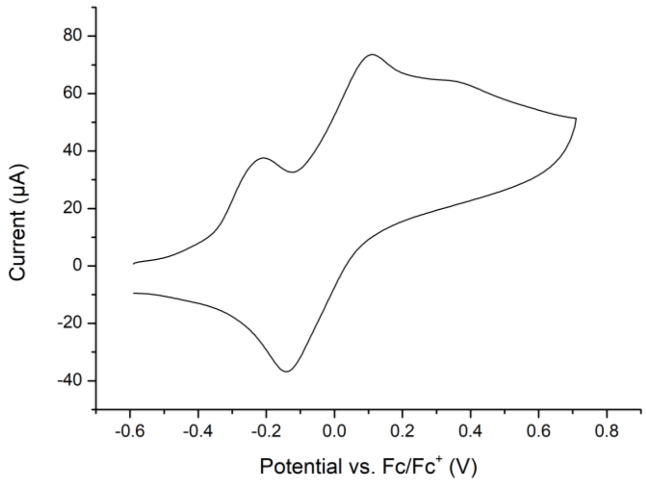

Figure S8: Cyclic voltammetry of **8** (referenced vs. Fc/Fc^{+}). Voltage sweep 250 mV/sec.

X-ray Crystallography

X-ray diffraction measurements were performed on a Stoe IPDS 2 diffractometer with an image plate detector at 100(2) K using monochromated (graded multilayer mirror) Mo Kα radiation (Mo Genix, compound **6**), on a Stoe StadiVari diffractometer with a Dectris Pilatus 200 K detector at 298(2) K (compound **4**) or 100(2) K (compounds **7** and **9**) using monochromated (plane graphite) Mo Kα radiation (Mo Genix for compounds **4**, **7** and **9**), or on a Nonius Kappa CCD diffractometer at 104(2) K using Mo Kα radiation (compound **8**). The data sets were recorded with ω-scans and corrected for Lorentz, polarization and absorption effects. The structures were solved using direct methods and refined without restraints by full-matrix least-squares techniques against F^2 (SHELXT and SHELXL-2014/7).[37] Details of the structure determinations and refinement for **4**, **6**, **7**, **8** and **9** are summarized in Table S3. CCDC-1883376-1883379 and 1872731 contain the supplementary crystallographic data for this paper. These data can be obtained free of charge from The Cambridge Crystallographic Data Centre via www.ccdc.cam.ac.uk/data_request/cif. Further programs used for analysis and visualization of structural information include WinGX and Mercury.[38]

Table S3: Summary of structure determinations and refinement for **4**, **6**, **7**, **8** and **9**.

	**4**	**6**	**7**	**8**	**9**
CCDC code	1883379	1883378	1883376	1872731	1883377
Empirical formula	$C_{36}H_{52}Fe_2P_6$	$C_{36}H_{53}Fe_2P_5Si$	$C_{36}H_{54}Fe_2P_4Si_2$	$C_{32}H_{52}Fe_2N_4P_2Si_4$	$C_{38}H_{56}Fe_2N_2P_4$
Formula weight	782.30	780.42	778.55	778.77	776.42
Crystal description	yellow plate	yellow block	yellow block	yellow plate	yellow plate
Crystal size [mm]	0.21 × 0.20 × 0.07	0.16 × 0.12 × 0.10	0.72 × 0.34 × 0.29	0.25 × 0.20 × 0.07	0.11 × 0.04 × 0.03
Crystal system, space group	monoclinic, $P2_1/n$	monoclinic, $P2_1/n$	monoclinic, $P2_1/n$	monoclinic, $C\,2/c$	orthorhombic, $P\,n\,a\,2_1$
Temperature [K]	298(2)	100(2)	100(2)	103(2)	100(2)

Unit cell dimensions:					
a [Å]	12.6525(5)	12.5620(5)	12.6059(6)	17.6548(13)	21.1103(12)
b [Å]	21.5629(10)	21.3264(9)	21.3427(9)	10.2983(7)	16.5319(9)
c [Å]	13.9177(5)	13.9675(6)	14.1442(6)	22.185(2)	10.7568(4)
α [°]	90.00	90.00	90.00	90.00	90.00
β [°]	91.561(3)	91.419(3)	91.330(4)	112.793(5)	90.00
γ [°]	90.00	90.00	90.00	90.00	90.00
Volume [Å^3]	3795.7(3)	3740.8(3)	3804.4(3)	3718.6(5)	3754.1(3)
Z	4	4	4	4	4
Calculated density	1.369	1.386	1.387	1.391	1.374
F(000)	1640	1640	1704	1640	1640
Linear absorption coefficient μ [mm^{-1}]	1.042	1.047	1.020	1.024	0.973
Transmission T_{min}/T_{max}	0.8132/0.9325	0.8705/0.9135	0.6320/0.7694	0.6892/0.7454	0.9451/0.9751
θ Range for data collection	2.146 - 25.998	1.743 - 25.716	1.728 - 31.002	1.991 - 30.529	1.929 - 25.999
Index ranges	$-15 \leq h \leq 15$<br>$-26 \leq k \leq 26$<br>$-15 \leq l \leq 17$	$-15 \leq h \leq 15$<br>$-25 \leq k \leq 25$<br>$-17 \leq l \leq 17$	$-13 \leq h \leq 18$<br>$-22 \leq k \leq 30$<br>$-20 \leq l \leq 15$	$-24 \leq h \leq 25$<br>$-14 \leq k \leq 8$<br>$-31 \leq l \leq 31$	$-26 \leq h \leq 26$<br>$-20 \leq k \leq 20$<br>$-13 \leq l \leq 13$
Refl. collected/unique [R_{int}]	19608/7410 [0.0263]	22995/7043 [0.0357]	32532/7079 [0.0520]	21175/3940 [0.0671]	27154/5389 [0.0728]
Final R_1 (wR_2) [$I > 2\sigma(I)$]	0.0325 (0.0738)	0.0677 (0.2132)	0.0447 (0.0960)	0.0400 (0.0749)	0.0538 (0.1096)
Final R_1 (wR_2) [all data]	0.0494 (0.0803)	0.0760 (0.2174)	0.0776 (0.1034)	0.0758 (0.0856)	0.0829 (0.1232)
Goodness-of-fit on F^2	0.994	1.217	0.891	1.007	1.021
Largest difference peak/hole [e Å^{-3}]	0.706/ -0.230	1.327/ -0.544	1.752/ -0.329	0.519/ -0.391	0.486/ -0.408

Thermal activation EPR measurements

The thermal activation of **4** was studied on saturated solutions in toluene or mesitylene under autogenous solvent atmosphere using standard pressure EPR tubes (Wilmad, 4 mm, Suprasil) equipped with a Teflon low pressure valve. The largest temperature range was accessible using a Varian E-109 spectrometer and mesitylene solutions. The sample was heated by a hot nitrogen stream in the EPR cavity. It was kept at the temperature of measurement 5 minutes before the start of the measurement. The temperature was raised by 10 K up to 433 K and closer to the boiling point of the solvent in 5 K steps up to 443 K. Similar experiments have been performed for bisferrocenophanes **6-9**. For **7** heating beyond the boiling point of mesitylene up to 523 K was performed using 1,3,5-tri-*tert*-butyl benzene as a solvent.

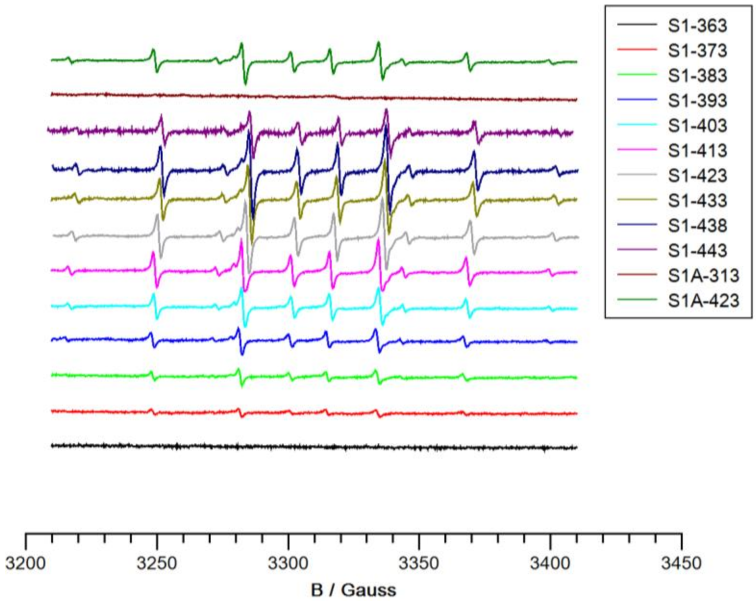

Figure S9: EPR spectra of **4** in mesitylene solution acquired sequentially with temperature in Kelvin quoted in the color code.

On the following pages the **NMR-spectra** of compounds **4**, **6**, **7** and **9** are depicted:

NMR-spectra **4**:

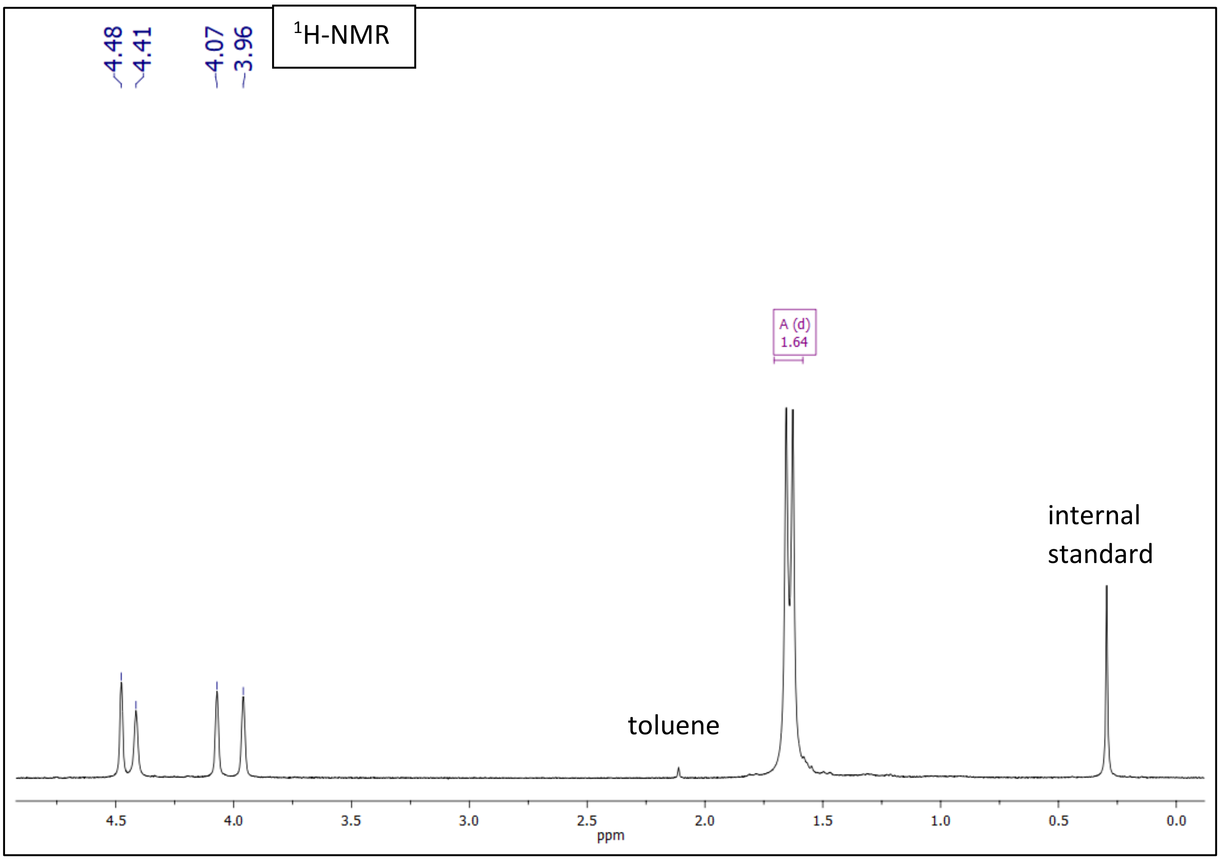

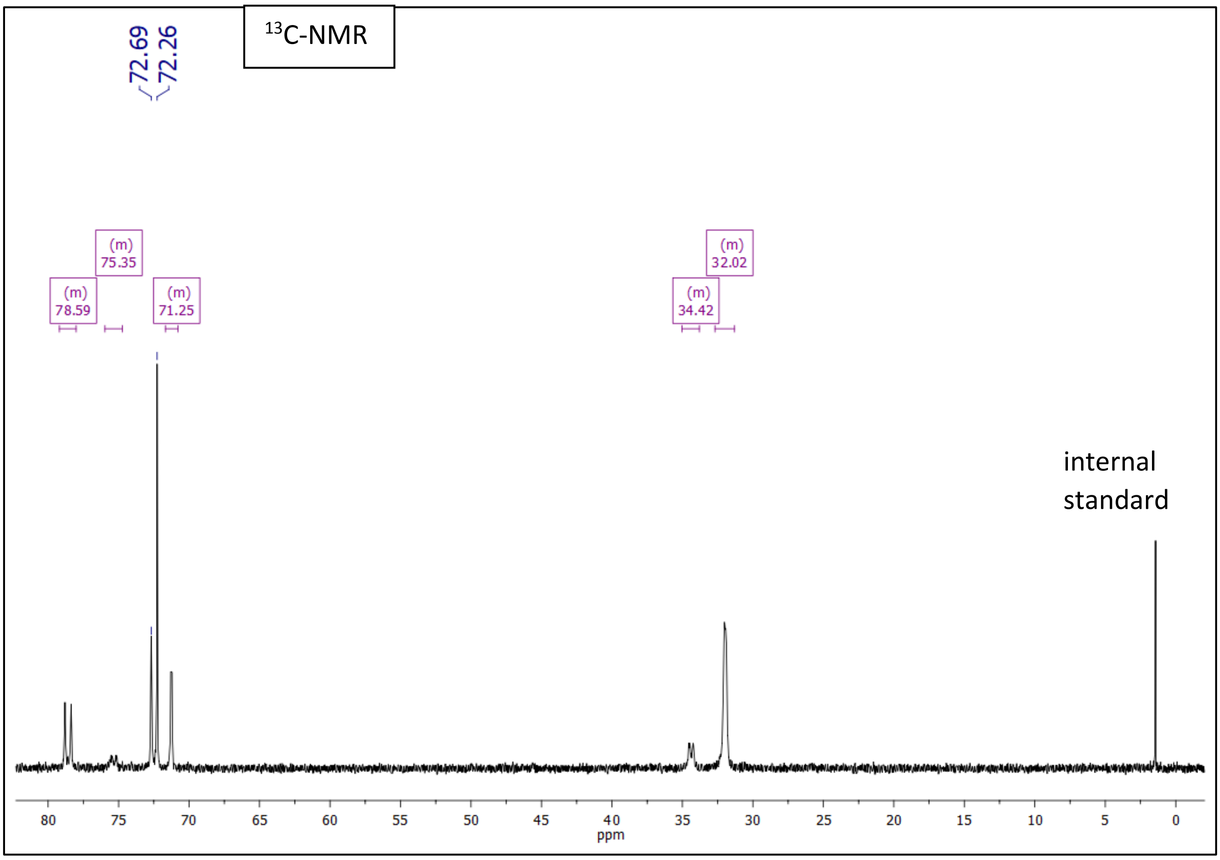

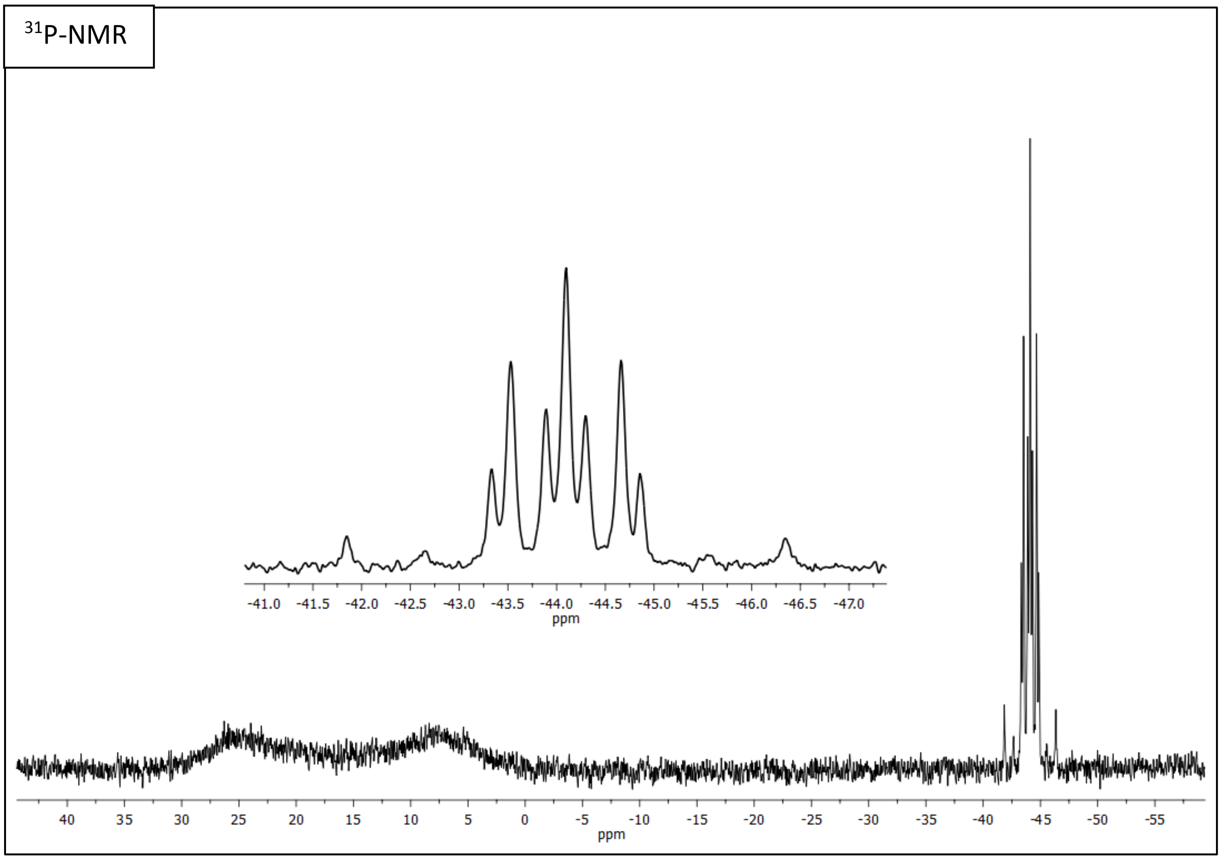
31P-NMR
-41.0 -41.5 -42.0 -42.5 -43.0 -43.5 -44.0 -44.5 -45.0 -45.5 -46.0 -46.5 -47.0
ppm
40 35 30 25 20 15 10 5 0 -5 -10 -15 -20 -25 -30 -35 -40 -45 -50 -55
ppm

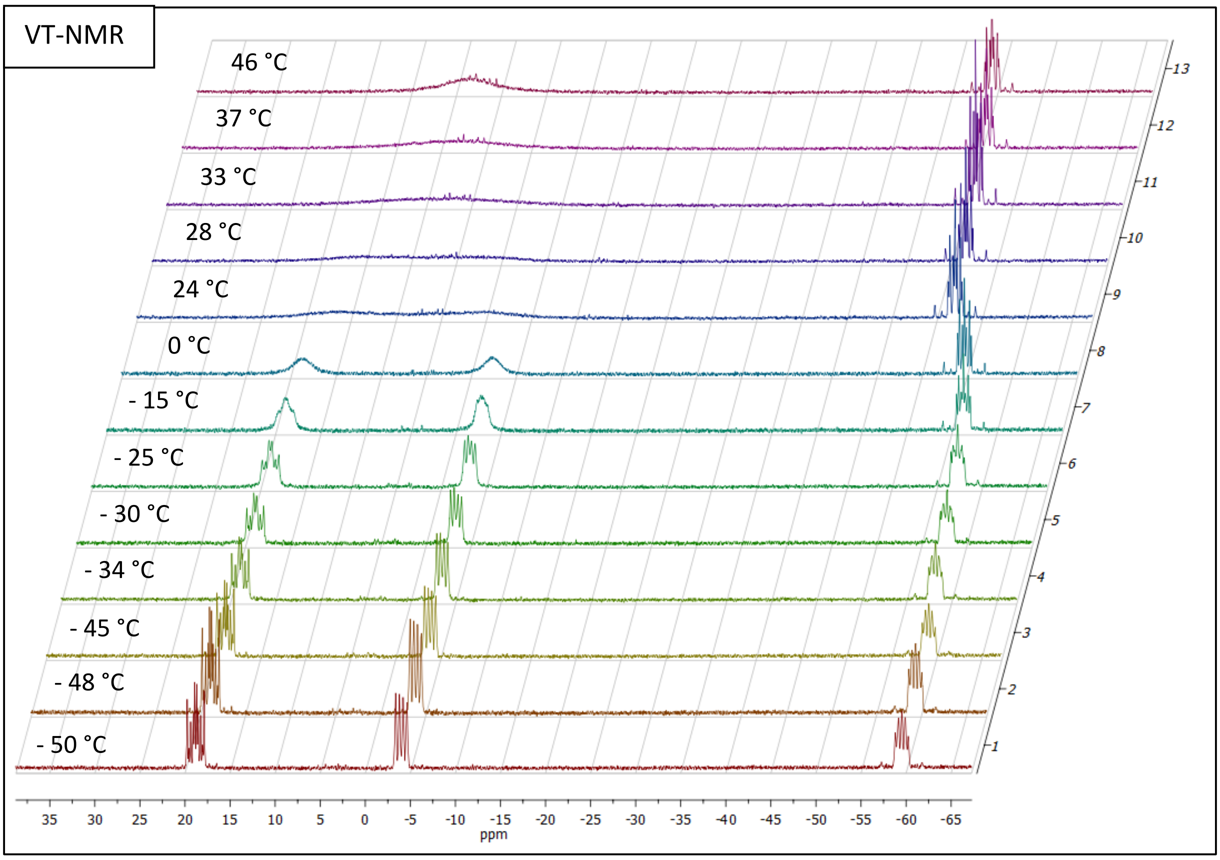
VT-NMR
46 °C
37 °C
33 °C
28 °C
24 °C
0 °C
- 15 °C
- 25 °C
- 30 °C
- 34 °C
- 45 °C
- 48 °C
- 50 °C
35 30 25 20 15 10 5 0 -5 -10 -15 -20 -25 -30 -35 -40 -45 -50 -55 -60 -65
ppm

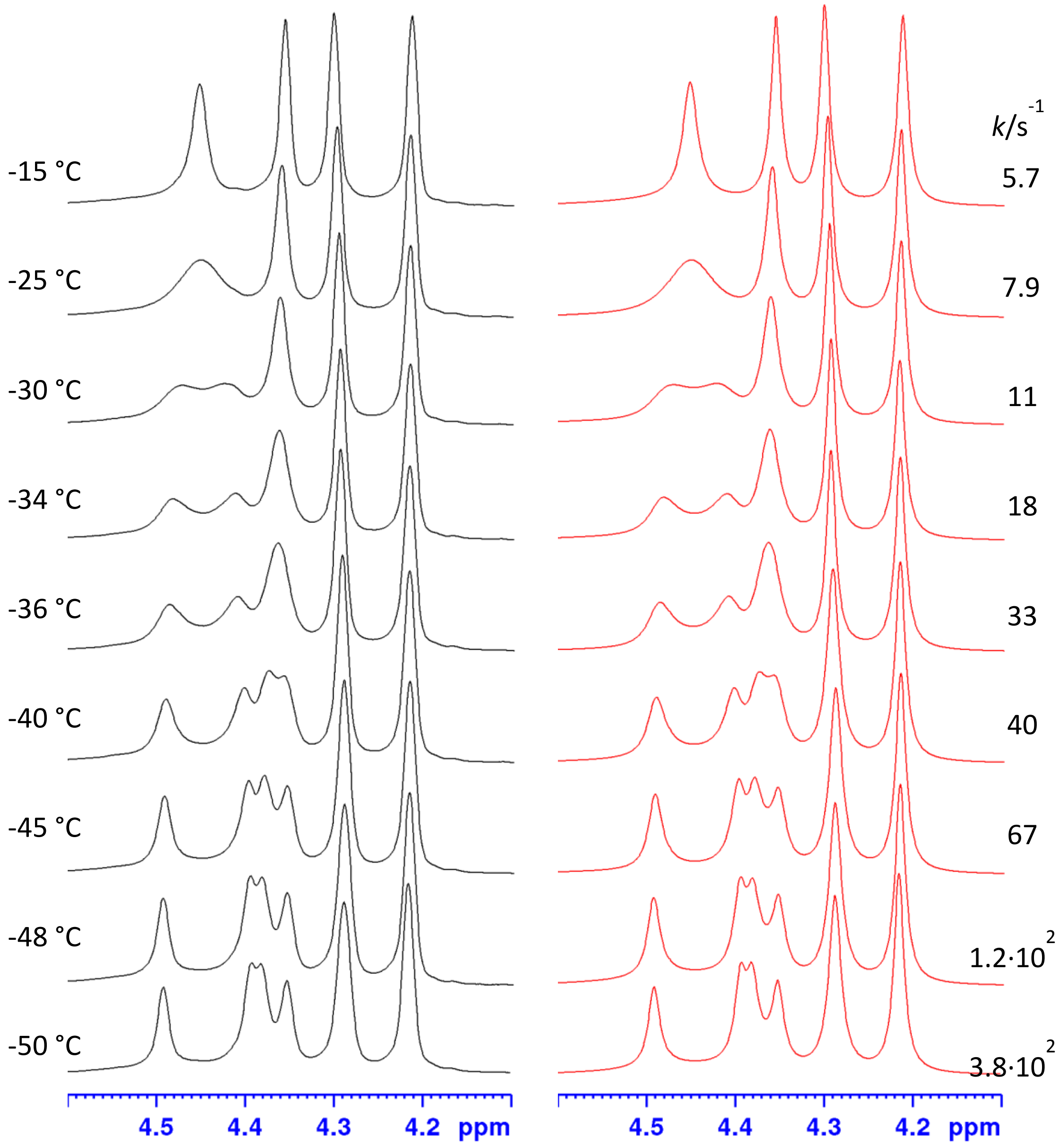

Figure S10: Expansions of measured (left, black traces) and simulated (right, red traces) ^{1}H NMR spectra of **4** showing the signals attributable to the Cp-units. Spectra were recorded in the temperature range between -50 and -15 °C. Simulation of dynamic NMR spectra was carried out by approaching the individual resonances by simple Lorentzian lines and neglecting unresolved spin couplings. The rate constants obtained from each fit is given on the right of the simulated spectrum.

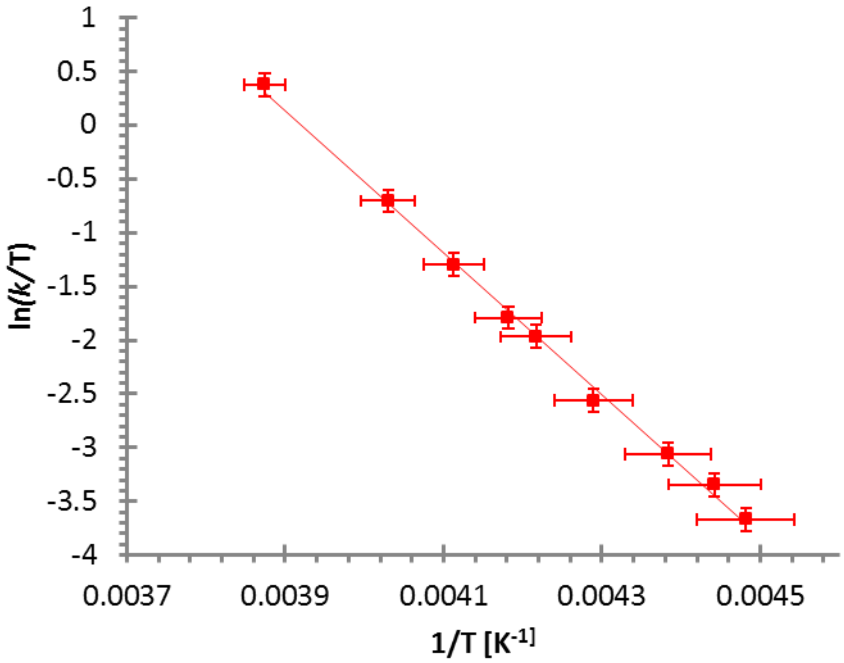

Figure S11: Eyring plot of ln(k/T) vs. 1/T for **4** based on fitted rate constants obtained from the ^{1}H NMR spectra shown in Figure S10. Linear regression yielded values of $\Delta H^{\#}$ = 54.9(11) kJ mol^{-1} and $\Delta S^{\#}$ = 17.7(4) J (K mol)$^{-1}$ (figures in parentheses denote estimated standard deviations; correlation coefficient for the linear regression R^2 = 0.9987).

NMR-spectra **6**:

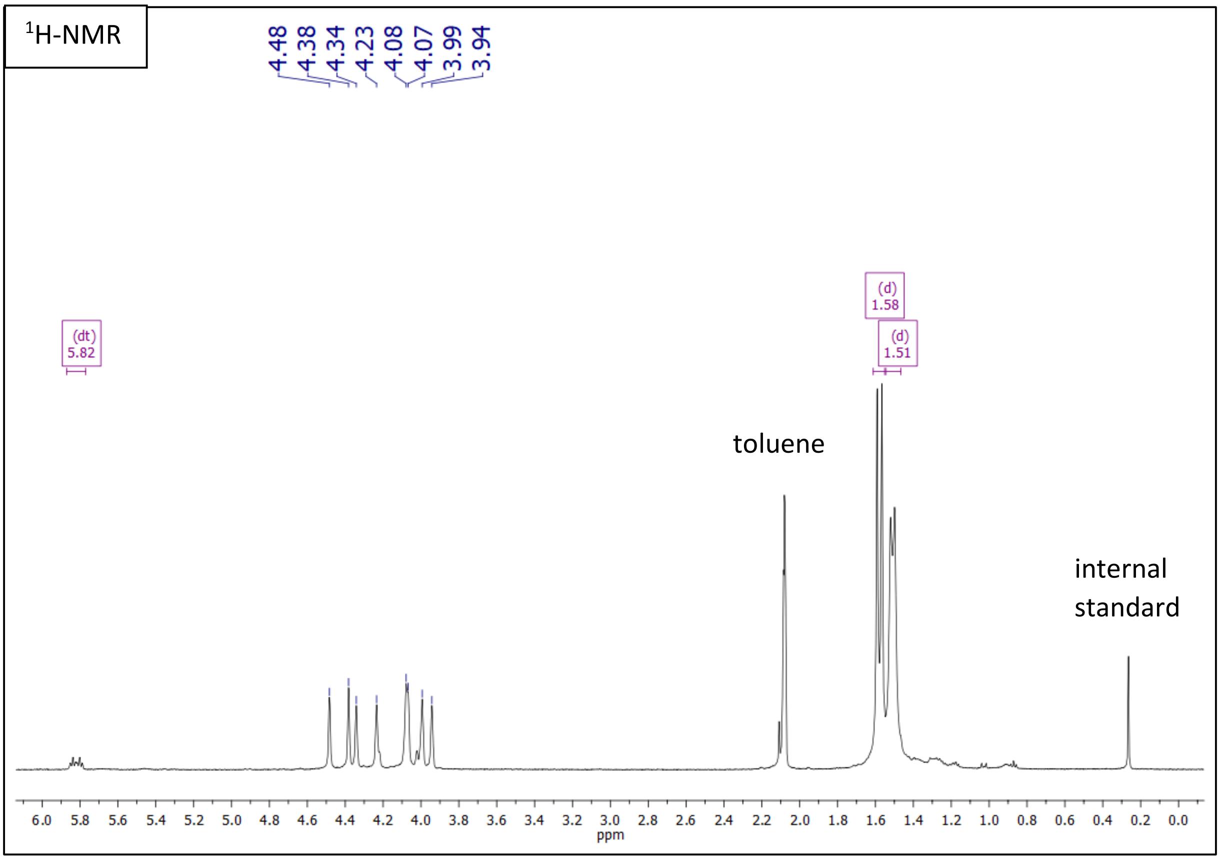

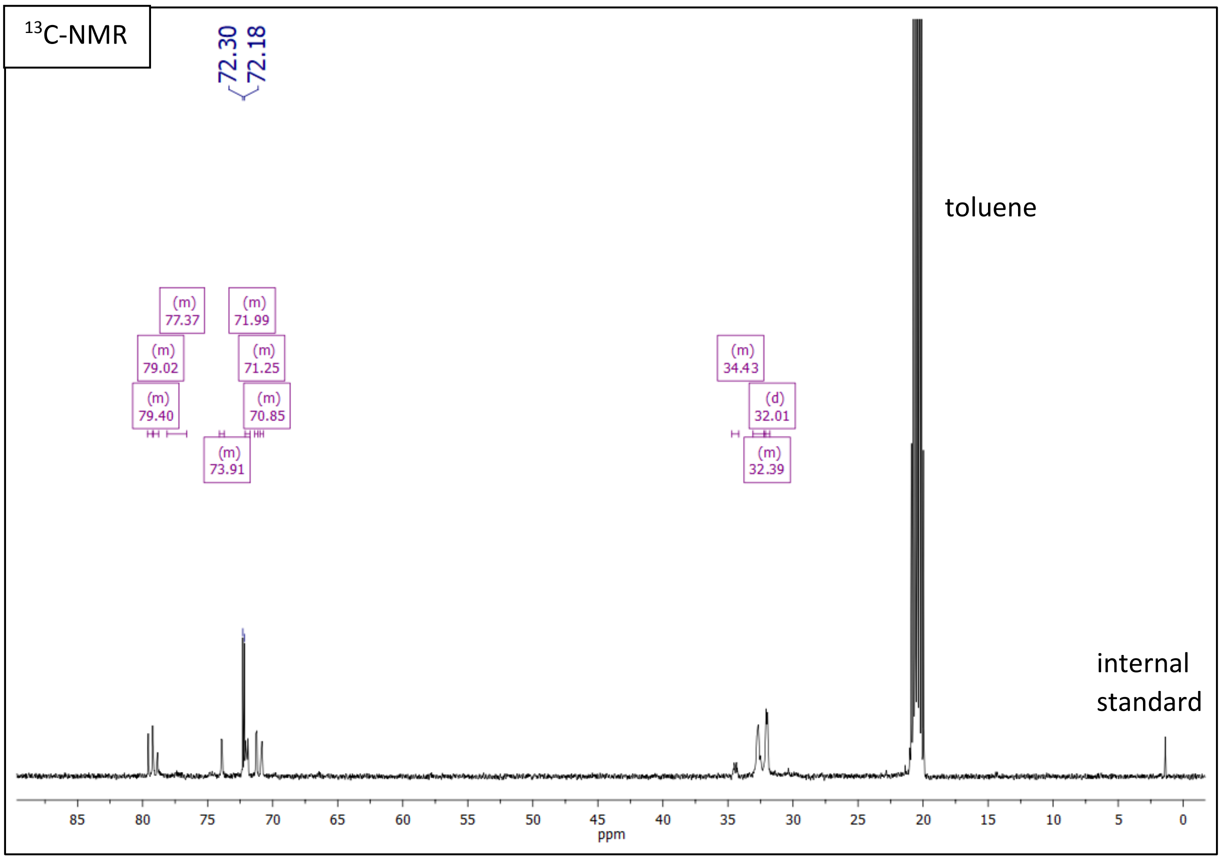

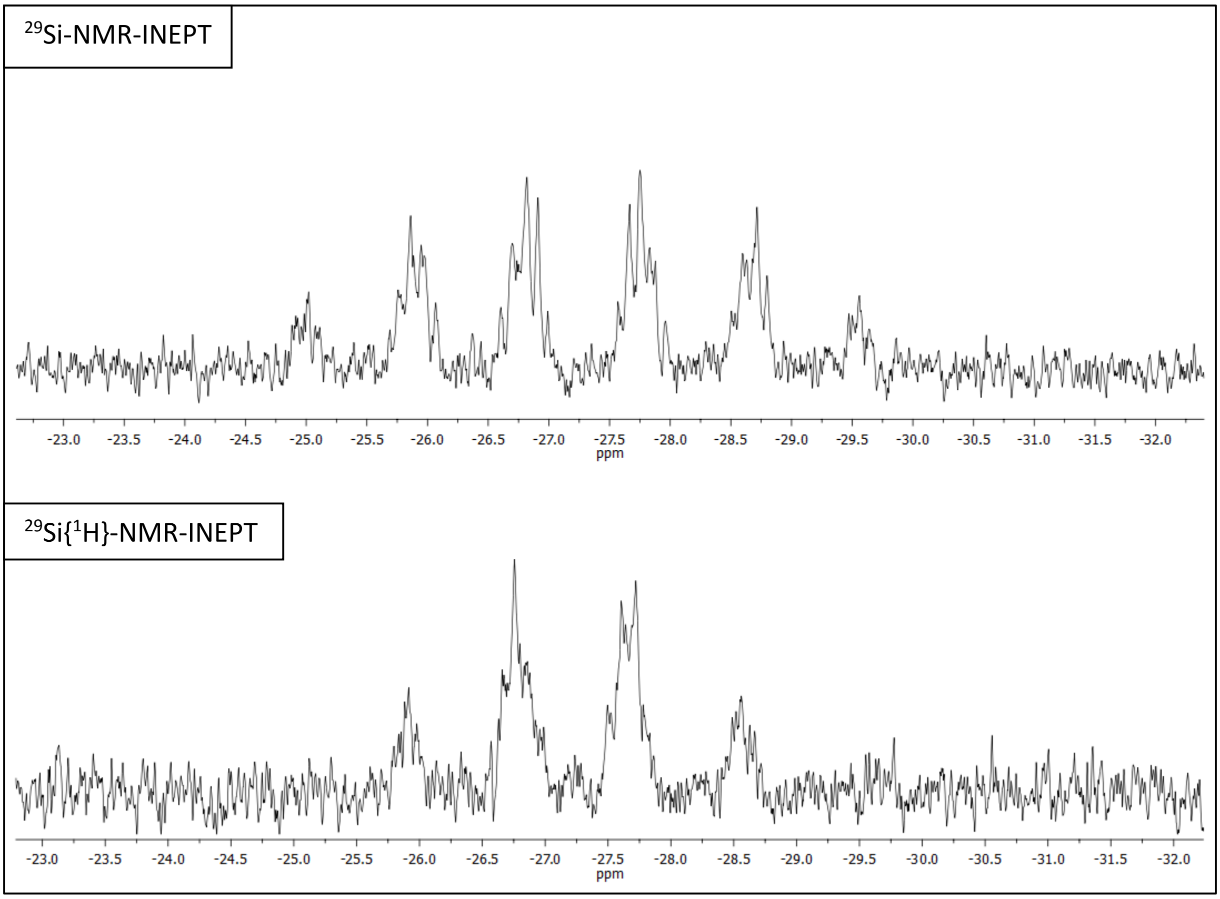
29Si-NMR-INEPT
-23.0 -23.5 -24.0 -24.5 -25.0 -25.5 -26.0 -26.5 -27.0 -27.5 -28.0 -28.5 -29.0 -29.5 -30.0 -30.5 -31.0 -31.5 -32.0
ppm
29Si{1H}-NMR-INEPT
-23.0 -23.5 -24.0 -24.5 -25.0 -25.5 -26.0 -26.5 -27.0 -27.5 -28.0 -28.5 -29.0 -29.5 -30.0 -30.5 -31.0 -31.5 -32.0
ppm

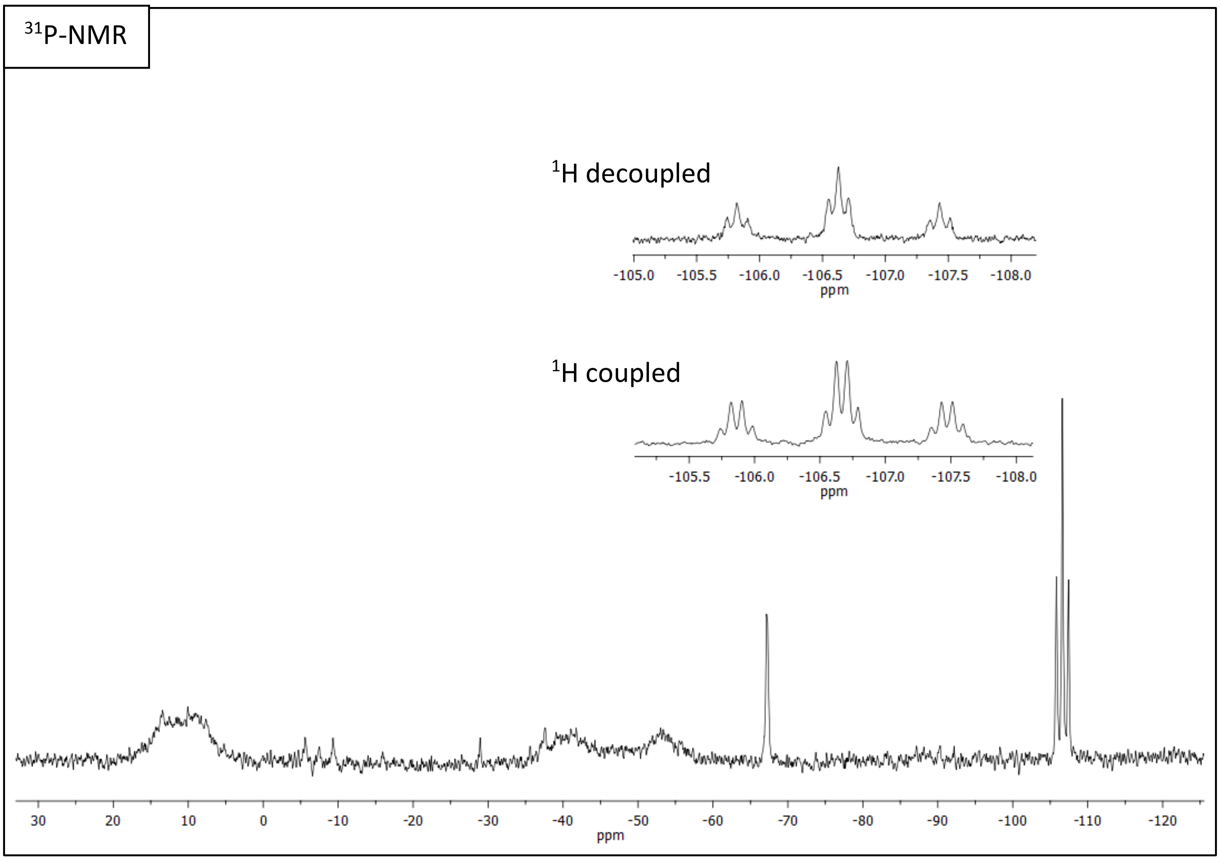
31P-NMR
1H decoupled
-105.0 -105.5 -106.0 -106.5 -107.0 -107.5 -108.0
ppm
1H coupled
-105.5 -106.0 -106.5 -107.0 -107.5 -108.0
ppm
30 20 10 0 -10 -20 -30 -40 -50 -60 -70 -80 -90 -100 -110 -120
ppm

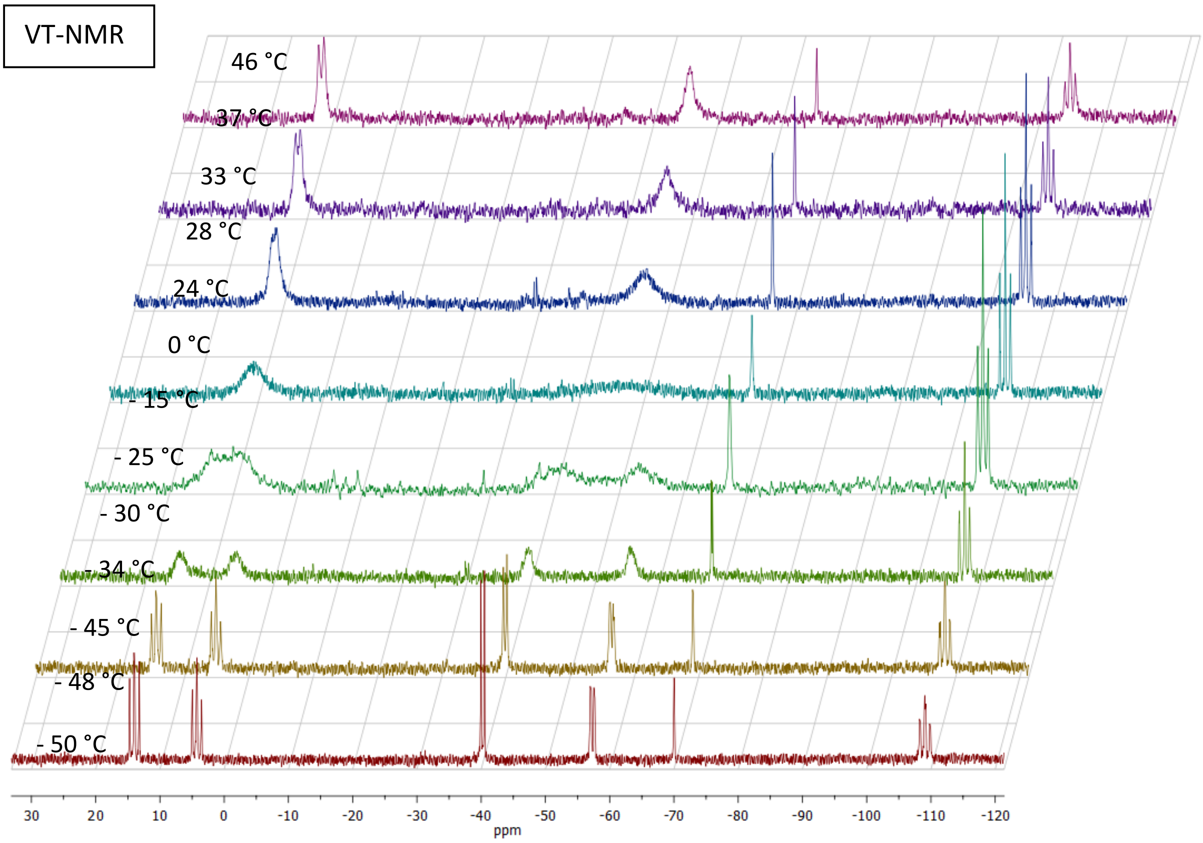
VT-NMR
46 °C
37 °C
33 °C
28 °C
24 °C
0 °C
- 15 °C
- 25 °C
- 30 °C
- 34 °C
- 45 °C
- 48 °C
- 50 °C
30 20 10 0 -10 -20 -30 -40 -50 -60 -70 -80 -90 -100 -110 -120
ppm

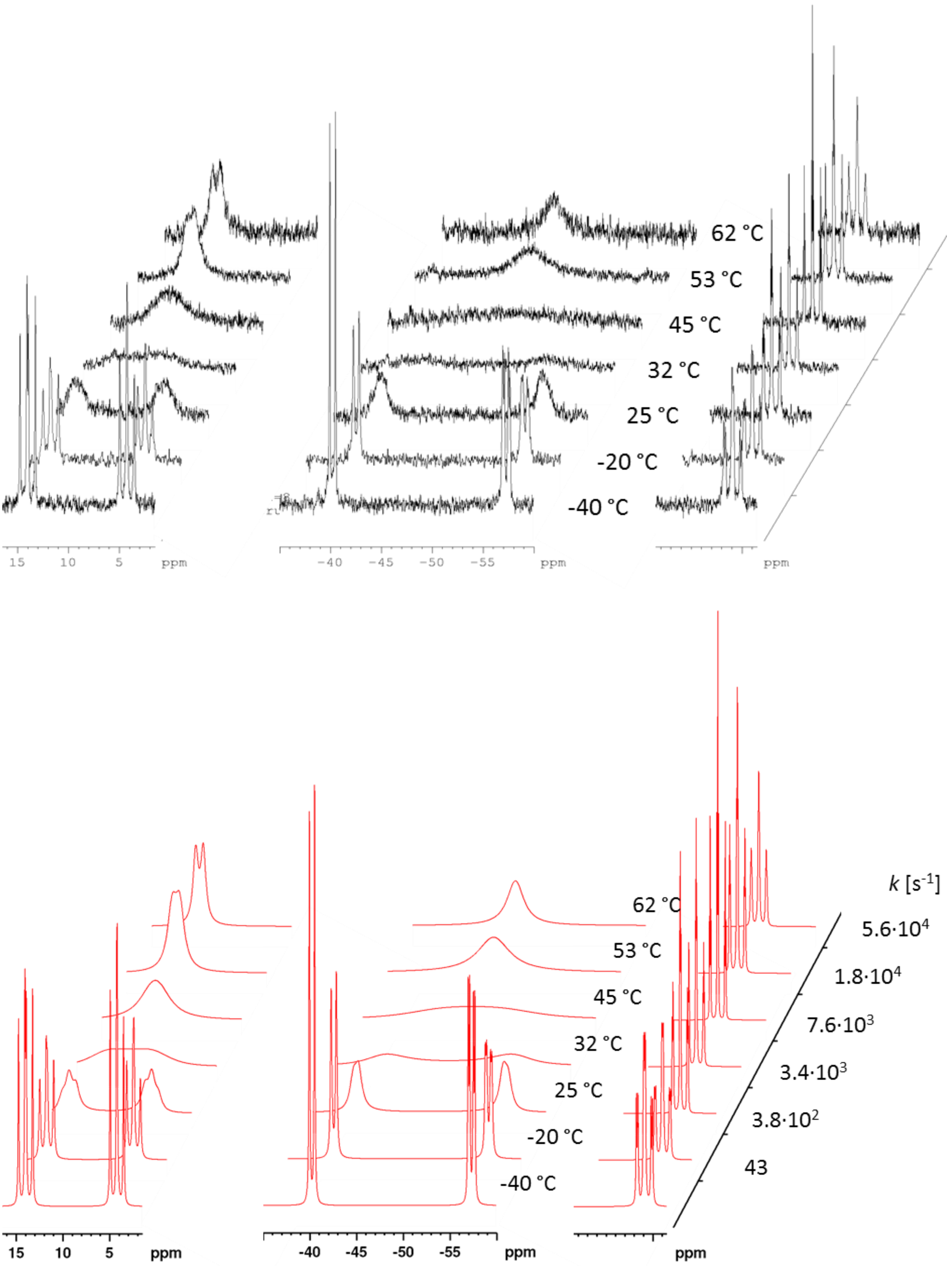

Figure S12: Stacked representation of measured (top, black traces) and simulated (bottom, red traces) $^{31}P\{^{1}H\}$ NMR spectra of **6** at temperatures between -40 and 62 °C. The rate constants obtained from the fits are given at the right of the simulated spectra.

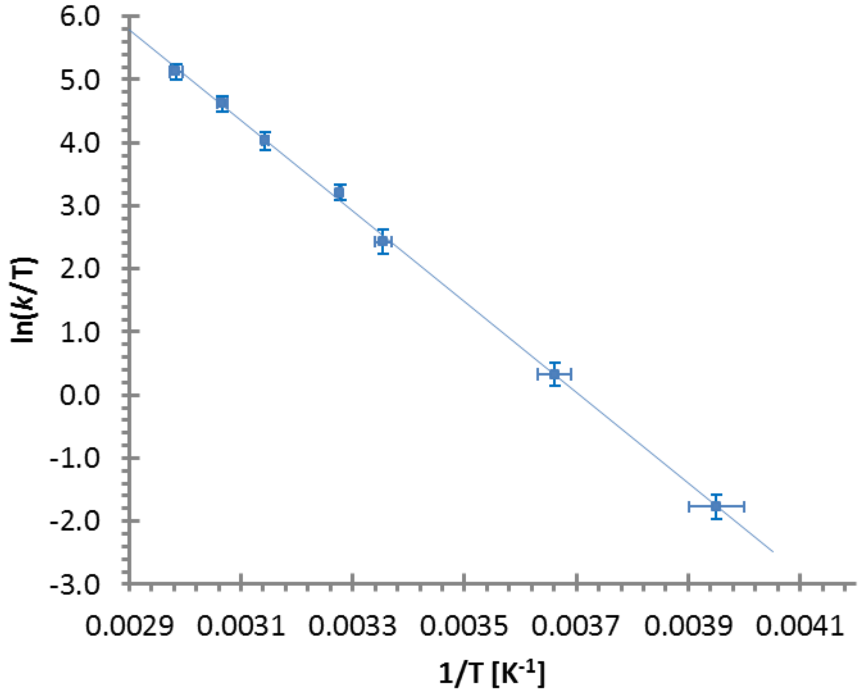

Figure S13: Eyring plot of ln(k/T) vs. 1/T for **6** based on fitted rate constants obtained from the $^{31}P\{^{1}H\}$ NMR spectra shown in Figure S13. Linear regression yielded values of $\Delta H^{\#}$ = 59.7(8) kJ mol^{-1} and $\Delta S^{\#}$ = 23.7(3) J (K mol)$^{-1}$ (figures in parentheses denote estimated standard deviations; correlation coefficient for the linear regression R^2 = 0.9987).

NMR-spectra **7**:

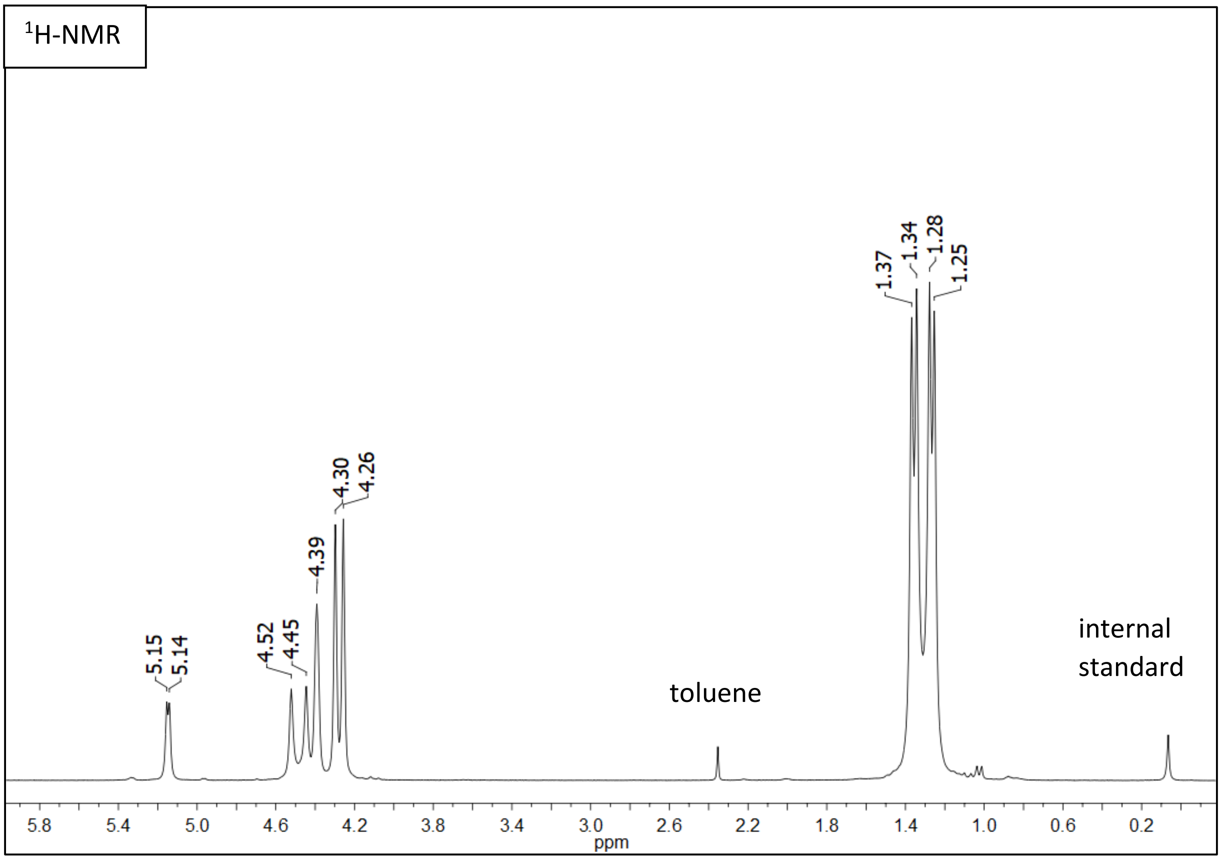

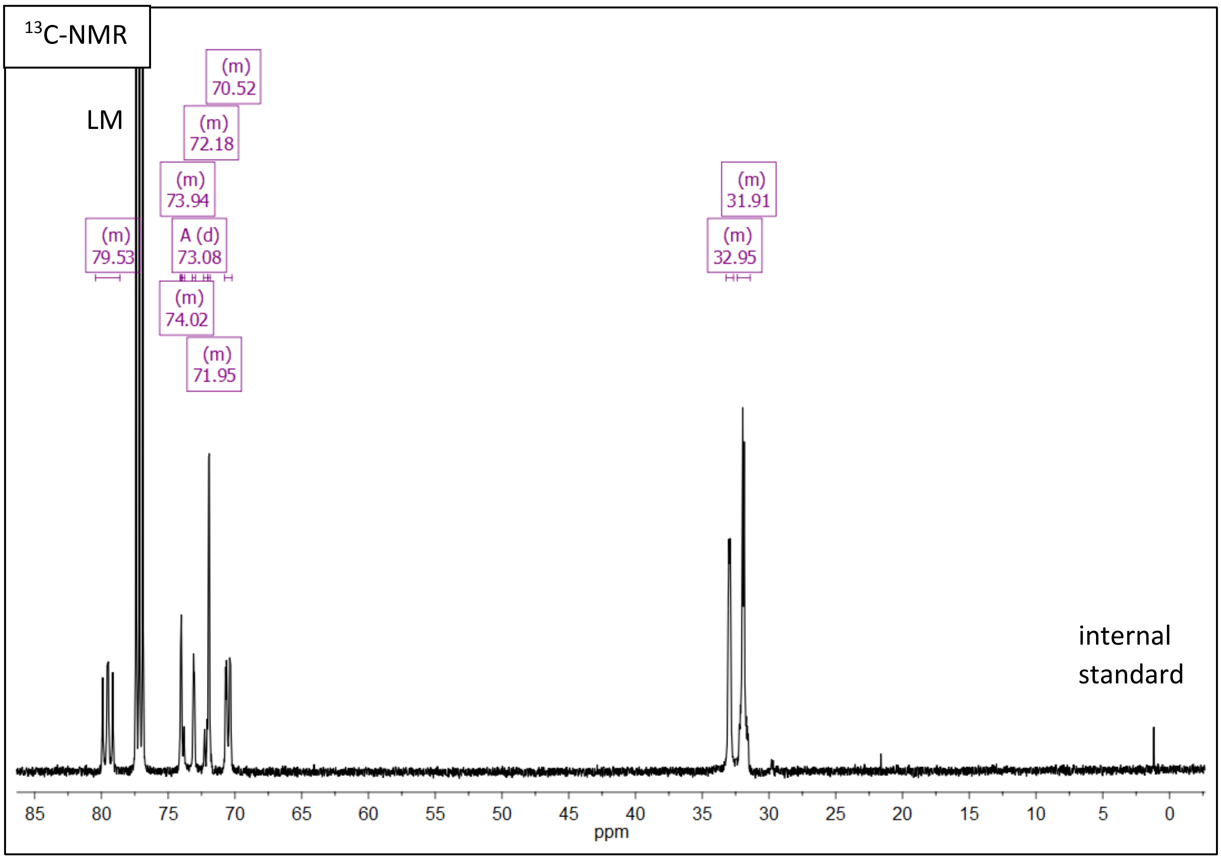

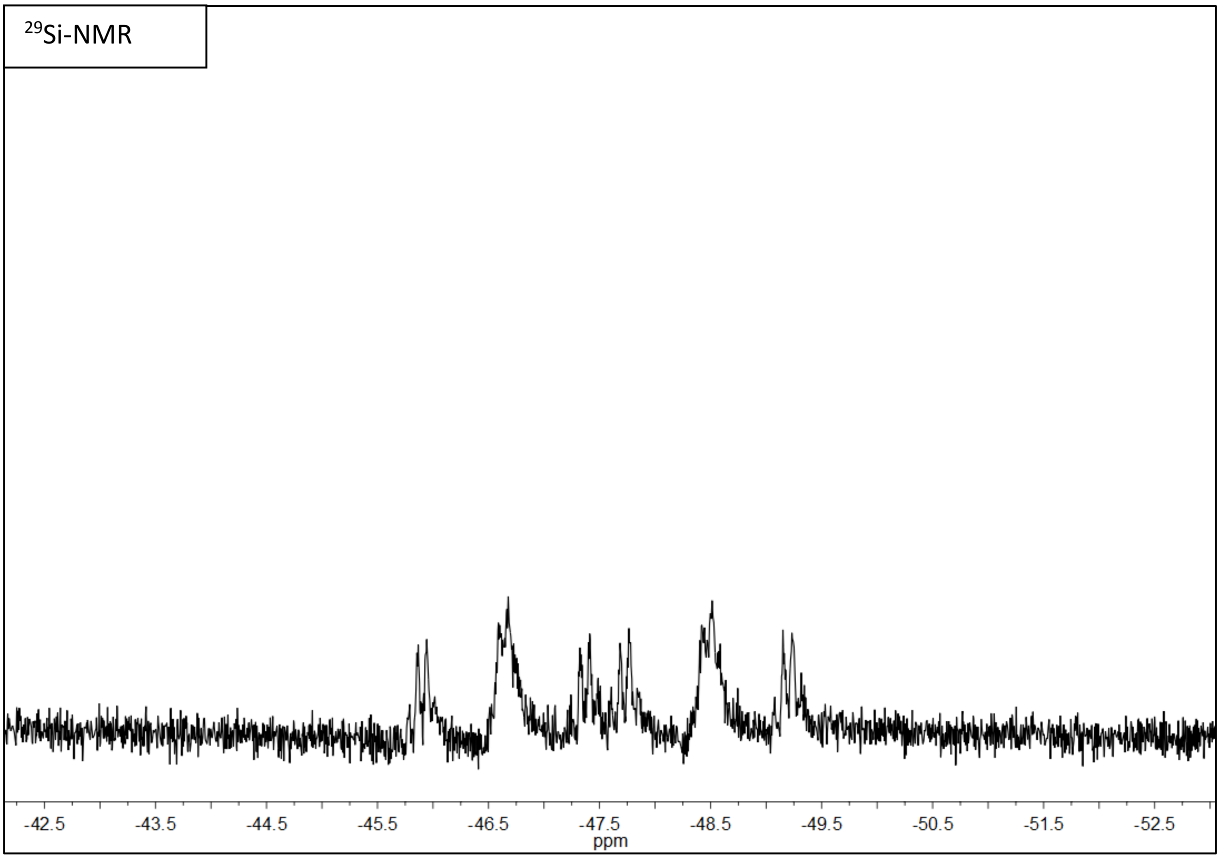

^{29}Si-NMR
-42.5
-43.5
-44.5
-45.5
-46.5
-47.5
-48.5
-49.5
-50.5
-51.5
-52.5
ppm

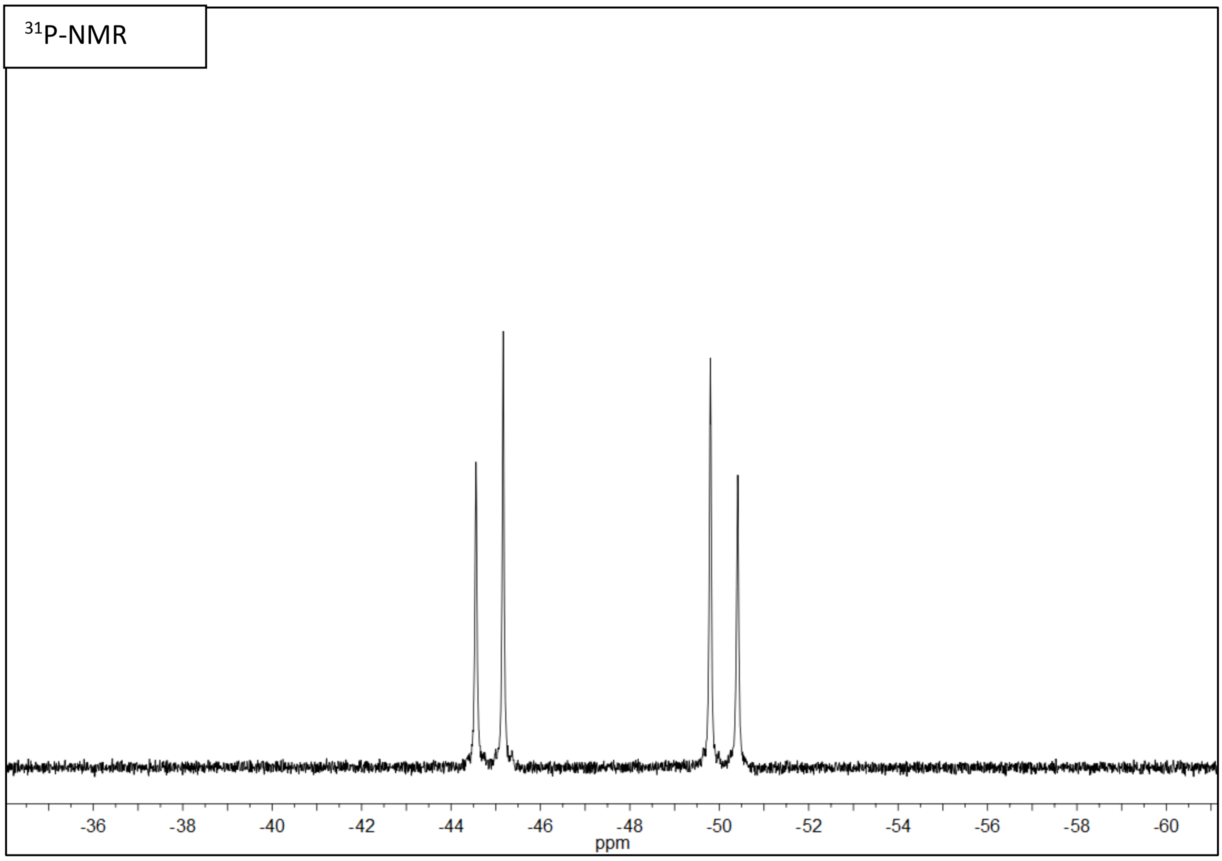

^{31}P-NMR
-36
-38
-40
-42
-44
-46
-48
-50
-52
-54
-56
-58
-60
ppm

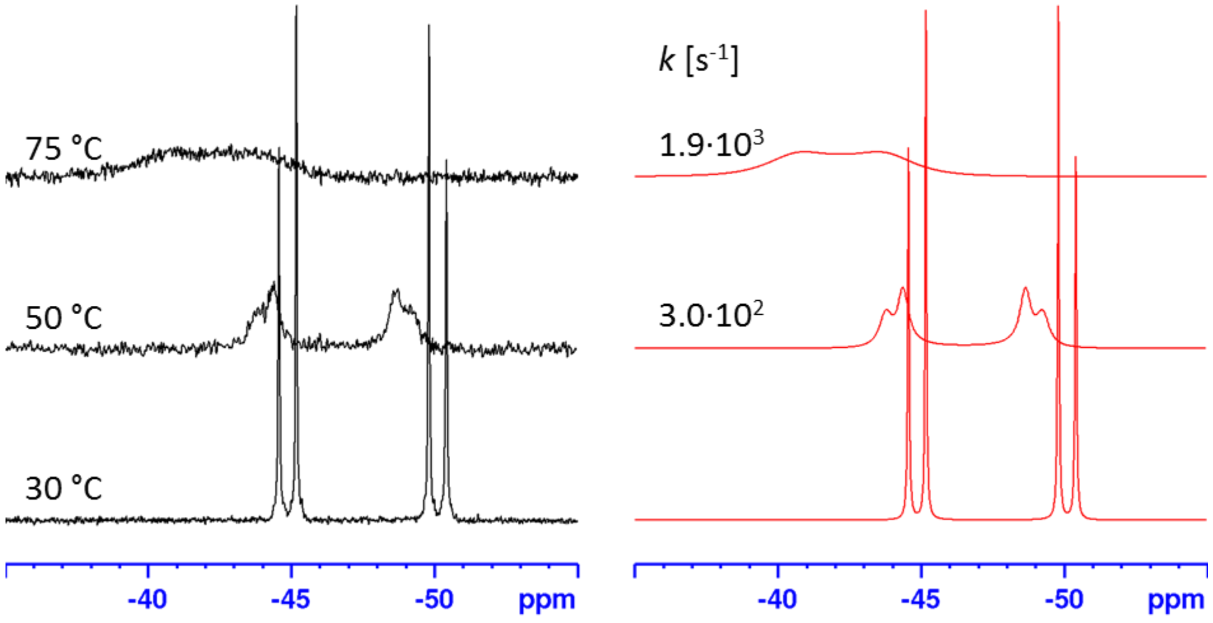

Figure S14: Measured (left, black traces) and simulated (right, red traces) $^{31}P\{^{1}H\}$ NMR spectra of **7** in toluene at temperatures between 30 and 75 °C. The rate constants obtained from spectral fits are given with the simulated spectra.

NMR-spectra **8**:

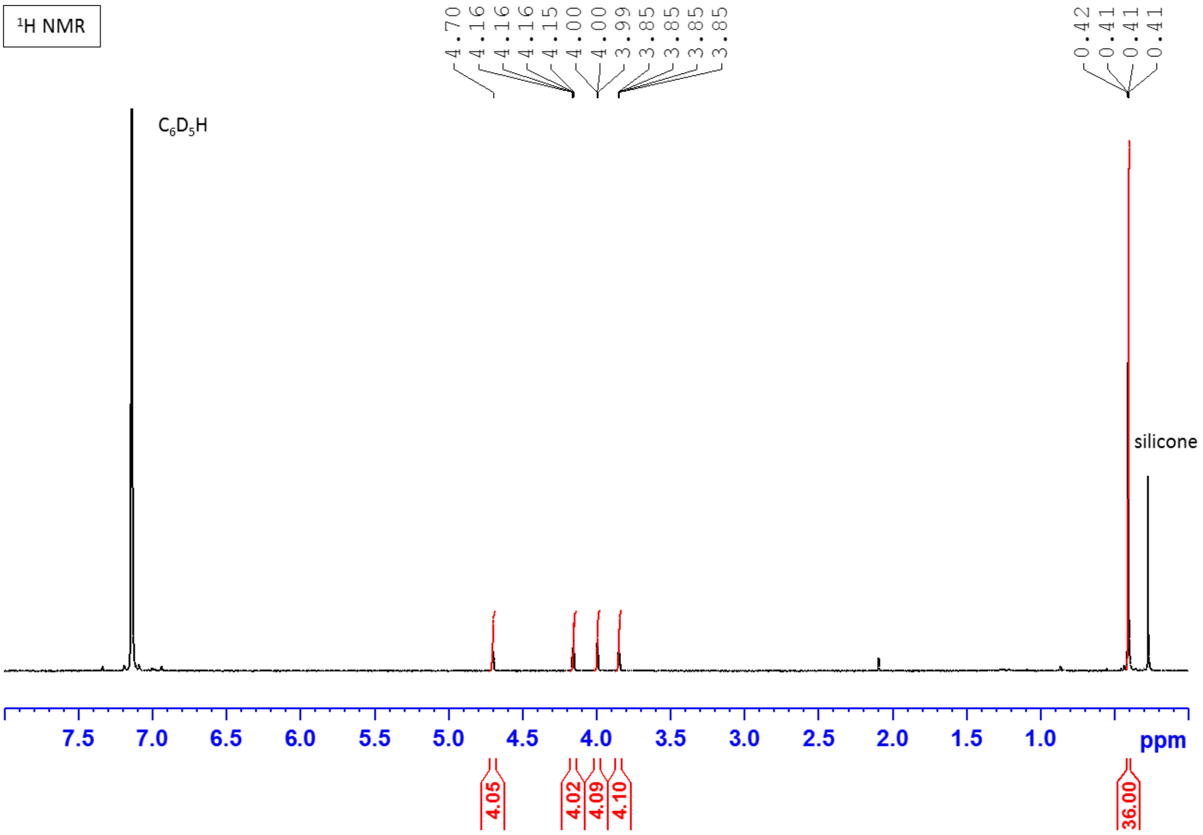

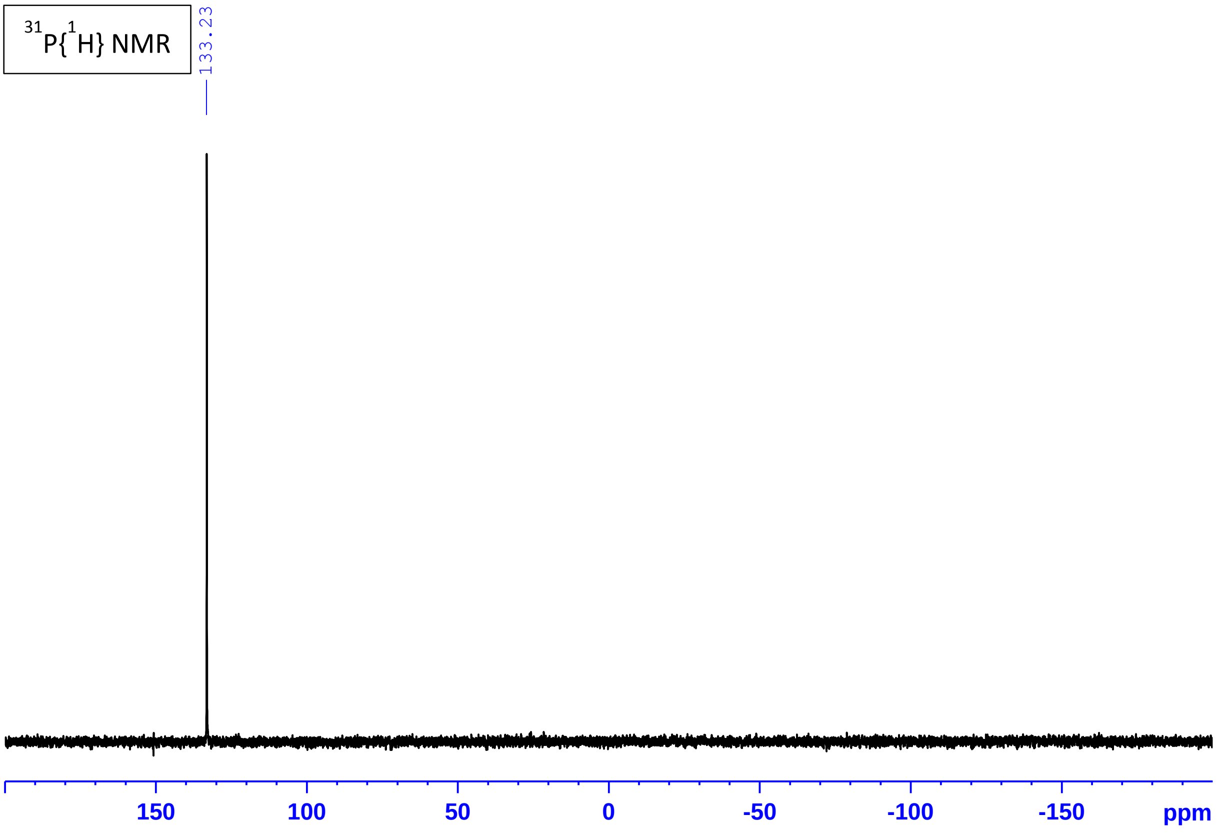

NMR-spectra **9**:

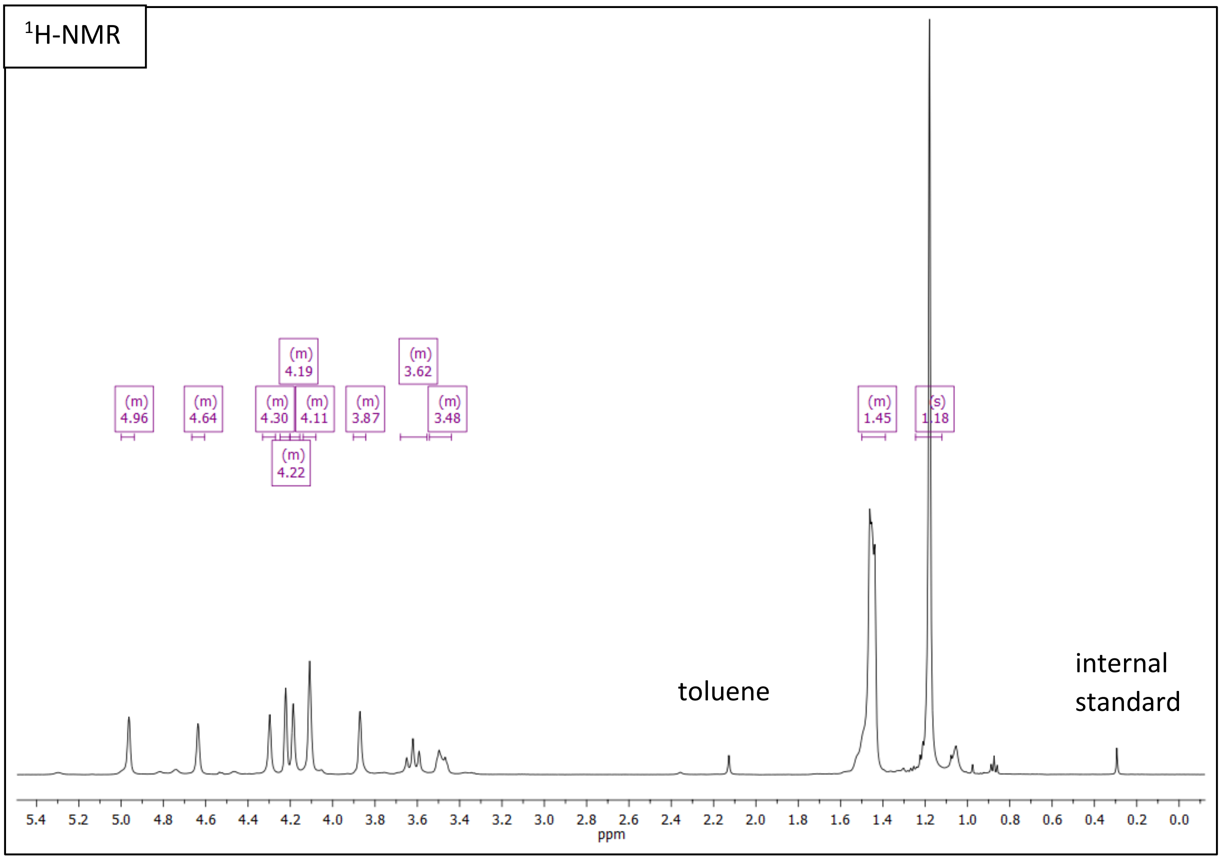

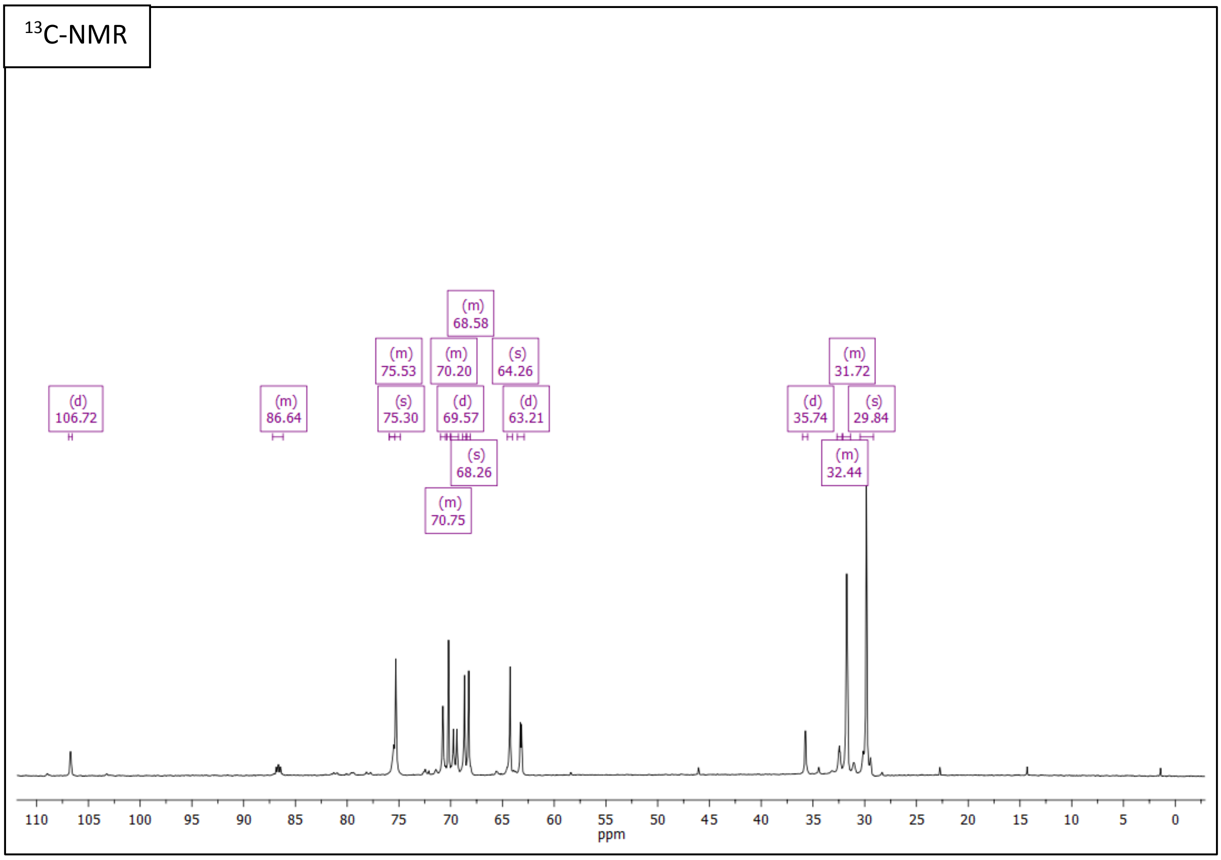

^{13}C-NMR
(d) 106.72
(m) 86.64
(m) 75.53
(s) 75.30
(m) 70.20
(m) 68.58
(d) 69.57
(s) 68.26
(m) 70.75
(s) 64.26
(d) 63.21
(d) 35.74
(m) 31.72
(s) 29.84
(m) 32.44
ppm

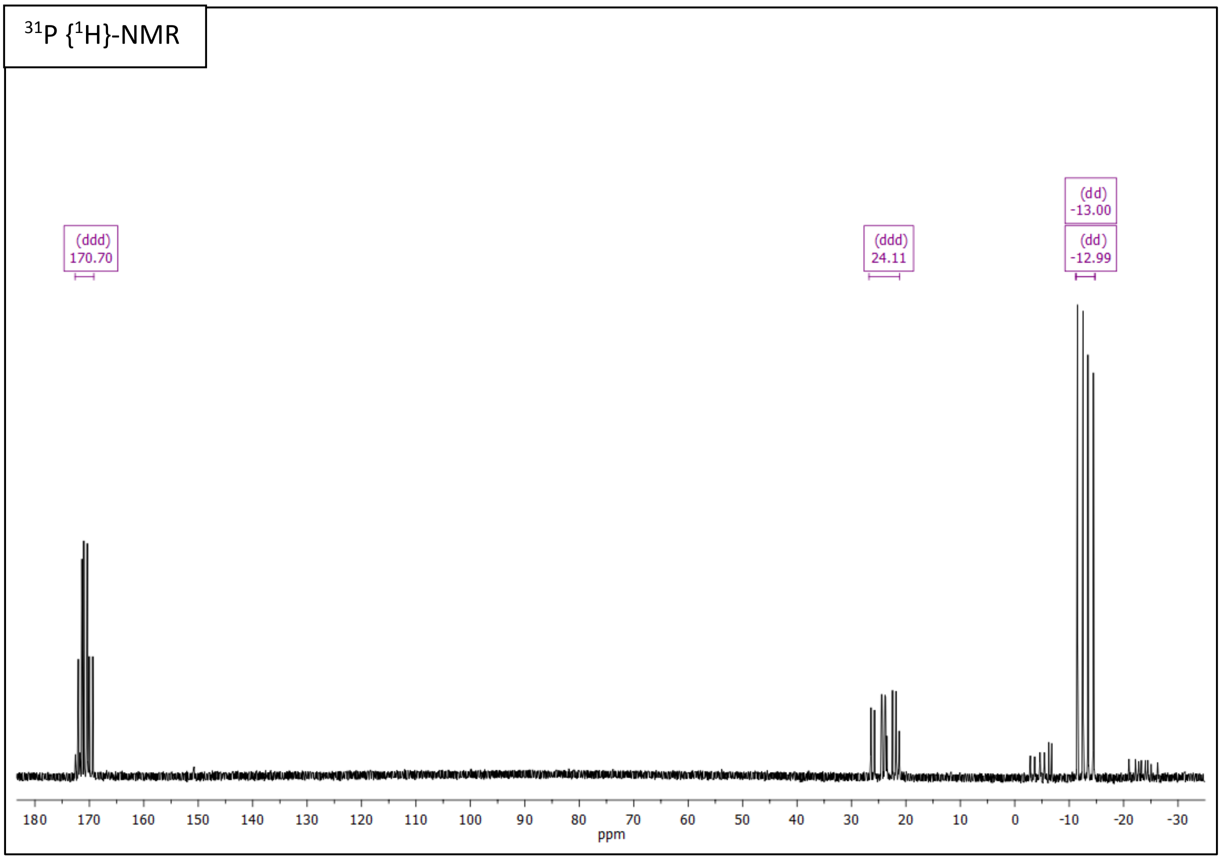

^{31}P {^{1}H}-NMR
(ddd) 170.70
(ddd) 24.11
(dd) -13.00
(dd) -12.99
ppm

XYZ coordinates and their total energies (in a.u.) of the investigated systems

4

G(ωB97XD/6-31+G*)= -5977.403820
E(ωB97XD/6-31+G*)= -5978.143491

C	6.020691	1.015401	-1.040876
C	5.501331	1.921186	-0.069681
C	4.086084	1.881494	-0.137764
C	3.702496	0.951340	-1.159261
C	4.915912	0.422223	-1.712813
Fe	4.799302	0.026007	0.281908
C	4.796642	-1.993723	0.541214
C	3.592300	-1.405873	1.055681
C	3.973276	-0.471374	2.066991
C	5.394855	-0.474266	2.168304
C	5.900170	-1.415716	1.229771
P	1.940195	-1.911822	0.438564
C	1.235205	-2.721697	2.021349
C	0.865522	-1.776516	3.166501
P	2.060320	0.476637	-1.812056
C	1.517206	2.162754	-2.536643
C	0.349635	1.911927	-3.498523
P	1.088583	0.180246	0.221062
P	-1.088677	-0.216079	0.231225
P	-1.900283	1.892682	0.423860
C	-1.174496	2.707529	1.994067
C	-2.244741	3.712292	2.455660
C	-3.558827	1.429731	1.042758
C	-4.760025	2.030982	0.541389
C	-5.868753	1.494179	1.247990
C	-5.368603	0.556138	2.195677
C	-3.951713	0.511413	2.077974
Fe	-4.800222	0.005884	0.310431
C	-6.043128	-0.976694	-0.980534
C	-4.941486	-0.408552	-1.677035
C	-3.731046	-0.944730	-1.127468
C	-4.106541	-1.854082	-0.089044
C	-5.528130	-1.875451	-0.000197
P	-2.080233	-0.511409	-1.792414
C	-1.586531	-2.209369	-2.527968
C	-2.789547	-2.706623	-3.348152
C	-1.201556	-3.270634	-1.499088
C	-0.418796	-1.987032	-3.490544
C	0.082458	3.488436	1.591860
C	-0.831107	1.764175	3.146030
C	-0.007155	-3.532609	1.626836
C	2.323858	-3.702586	2.493357
C	1.118893	3.221219	-1.515248
C	2.711545	2.673861	-3.364986
H	4.844557	-2.724712	-0.254899
H	6.943675	-1.635272	1.042343
H	5.995228	0.142511	2.824374
H	3.307673	0.189751	2.606054
H	3.400515	2.421529	0.502248

H	6.091957	2.496628	0.631995
H	7.063422	0.789864	-1.220493
H	4.968680	-0.325633	-2.493650
H	0.601250	-2.369974	4.052516
H	-0.000839	-1.167092	2.899945
H	1.692171	-1.116394	3.443125
H	-0.815259	-2.886257	1.274475

E(ωB97XD/6-31G*)= -5978.079819

C	5.393914	0.446107	-2.145963
C	5.893295	1.399285	-1.213999
C	4.782563	1.977984	-0.543789
C	3.580745	1.386017	-1.051746
C	3.975886	0.433340	-2.048061
Fe	4.789635	-0.027305	-0.265727
C	4.893994	-0.382074	1.723552
C	3.695112	-0.936311	1.166582
C	4.099227	-1.873168	0.159234
C	5.519167	-1.888392	0.103069
C	6.010817	-0.965722	1.069263
P	2.041217	-0.478157	1.794678
C	1.497370	-2.135672	2.556325
C	1.146706	-3.242277	1.564036
P	1.918614	1.888153	-0.476712
C	1.222293	2.650304	-2.086264
C	-0.031757	3.465604	-1.733741
P	1.085704	-0.201899	-0.244390
P	-1.085554	0.201503	-0.244595
P	-1.918723	-1.888599	-0.475391
C	-1.222574	-2.651855	-2.084560
C	0.031541	-3.466847	-1.731542
C	-3.580968	-1.386911	-1.050515
C	-3.976352	-0.435248	-2.047703
C	-5.394392	-0.448081	-2.145214
C	-5.893544	-1.400281	-1.212128
C	-4.782645	-1.978351	-0.541638
Fe	-4.789603	0.027266	-0.265622
C	-5.518569	1.889005	0.101342
C	-4.098638	1.873330	0.157535
C	-3.694870	0.937315	1.165797
C	-4.893952	0.384029	1.723276
C	-6.010552	0.967411	1.068383
P	-2.041078	0.479357	1.794329
C	-1.497222	2.137378	2.554908
C	-1.145820	3.242991	1.561759
C	-2.675105	2.588131	3.438102
C	-0.288848	1.863544	3.459445
C	-2.307186	-3.628777	-2.574164
C	-0.875904	-1.671030	-3.206071
C	0.288488	-1.861367	3.460012
C	2.674959	-2.585314	3.440501
C	0.875541	1.668577	-3.206958
C	2.306900	3.626828	-2.576712
H	-4.822790	-2.726043	0.239309
H	-6.935723	-1.623848	-1.028053

H	-5.991969	0.181140	-2.791255
H	-3.311775	0.219305	-2.594475
H	-3.435704	2.436405	-0.485152
H	-6.120223	2.477029	-0.578901
H	-7.050829	0.736152	1.253854
H	-4.926015	-0.368388	2.499953
H	-0.607372	-2.232600	-4.111061
H	-0.015817	-1.052994	-2.936135
H	-1.713807	-1.016828	-3.461401
H	0.839187	-2.824814	-1.367816
H	0.391302	-3.982325	-2.631598
H	-0.180419	-4.225372	-0.970785
H	-2.630431	-4.312525	-1.780145
H	-1.895060	-4.237589	-3.388804
H	-3.189334	-3.106261	-2.954393
H	-0.012406	2.786544	3.985374
H	0.586747	1.531796	2.894498
H	-0.518002	1.101702	4.212448
H	-0.240615	2.994272	1.001445
H	-0.945511	4.175736	2.105941
H	-1.956637	3.440451	0.854800
H	-3.537861	2.901971	2.843662
H	-2.352494	3.441128	4.048270
H	-3.001048	1.795354	4.121971
H	4.822925	2.726460	0.236394
H	6.935514	1.623059	-1.030407
H	5.991347	-0.183785	-2.791483
H	3.311151	-0.221799	-2.593947
H	4.925755	0.371123	2.499484
H	7.051019	-0.733968	1.254545
H	6.121016	-2.476876	-0.576602
H	3.436530	-2.437120	-0.482927
H	2.630305	4.311152	-1.783247
H	1.894686	4.235099	-3.391716
H	3.188951	3.103996	-2.956722
H	-0.391619	3.980313	-2.634194
H	0.180353	4.224758	-0.973655
H	-0.839349	2.823899	-1.369329
H	0.015465	1.050784	-2.936420
H	1.713453	1.014150	-3.461706
H	0.606995	2.229346	-4.112437
H	3.538114	-2.899408	2.846764
H	2.352366	-3.437898	4.051261
H	3.000321	-1.791829	4.123821
H	0.946234	-4.174507	2.109039
H	1.957908	-3.440344	0.857693
H	0.241785	-2.994230	1.002993
H	0.516984	-1.098785	4.212468
H	0.012071	-2.783986	3.986616
H	-0.586916	-1.530365	2.894333

TS of the inversion of the central P in **4**

E(ωB97XD/6-31G*)= -5978.052788

C	5.053361	-1.078524	-2.642083
C	5.470132	0.159589	-2.077211
C	4.310003	0.853751	-1.640976
C	3.156108	0.058308	-1.945697
C	3.637898	-1.140074	-2.564424
Fe	4.440337	-0.933056	-0.708376
C	3.506863	-2.194044	0.568672
C	3.715714	-0.920284	1.193050
C	5.130264	-0.693602	1.184953
C	5.766726	-1.800786	0.561857
C	4.761049	-2.731950	0.186206
P	2.338384	-0.000100	1.974977
C	3.030709	1.668587	2.537558
C	1.807126	2.495006	2.956857
P	1.367473	0.444208	-1.843379
C	1.274186	2.272464	-2.392694
C	2.081203	2.369506	-3.699780
P	0.988679	0.115708	0.292364
P	-1.010882	-0.095688	1.050059
P	-2.057655	1.785725	0.474148
C	-2.891473	2.321958	2.092284
C	-1.758251	2.575063	3.100502
C	-3.399405	1.340280	-0.688594
C	-3.128930	0.803282	-1.990917
C	-4.343282	0.734293	-2.722094
C	-5.383587	1.230243	-1.891470
C	-4.810534	1.600102	-0.644394
Fe	-4.459970	-0.356500	-1.015241
C	-5.487450	-2.095447	-1.300267
C	-4.087943	-2.290342	-1.445576
C	-3.439912	-1.893642	-0.226913
C	-4.473830	-1.473028	0.668416
C	-5.725674	-1.584226	0.007159
P	-1.623499	-1.944402	-0.086668
C	-1.416827	-3.264290	1.273215
C	0.084336	-3.461537	1.517733
C	-2.120810	-2.964067	2.599367
C	-1.997714	-4.554971	0.668243
C	-3.572174	3.673289	1.808552
C	-3.878451	1.330136	2.714211
C	3.879414	1.366664	3.786653
C	3.835409	2.472814	1.520763
C	-0.203548	2.533154	-2.722688
C	1.743214	3.326800	-1.395908
H	5.646292	0.174913	1.566491
H	6.829858	-1.900017	0.386708
H	4.918501	-3.665801	-0.336598
H	2.540040	-2.640908	0.384917
H	4.307397	1.815313	-1.151220
H	6.491414	0.497703	-1.964027
H	5.702598	-1.849890	-3.034349
H	3.011417	-1.956520	-2.896552
H	-5.367438	1.988380	0.194192
H	-6.432884	1.292733	-2.145907
H	-4.457321	0.337047	-3.721758

H	-2.163649	0.454318	-2.336135

TS of the inversion of the outer P in **4**

E(ωB97XD/6-31G*)= -5978.0289931

C	-6.177959	1.084403	0.004167
C	-6.018073	0.157210	1.072932
C	-4.649182	0.157207	1.458240
C	-3.946073	1.091020	0.629141
C	-4.909410	1.661776	-0.265273
Fe	-4.814847	-0.349217	-0.478655
C	-4.032742	-2.194179	-0.551785
C	-3.295278	-1.308600	-1.406617
C	-4.227478	-0.792461	-2.367162
C	-5.506990	-1.351726	-2.108590
C	-5.385172	-2.223976	-0.989913
P	-1.514091	-0.894286	-1.502835
C	-0.780028	-2.643845	-1.677105
C	-1.274097	-3.144143	-3.046133
P	-2.217487	1.670761	0.650584
C	-2.107734	2.364874	2.430511
C	-3.396198	3.174608	2.661489
P	-1.090119	-0.295928	0.639763
P	0.946577	0.703850	0.742380
P	2.223509	-0.949004	1.415595
C	1.852279	-1.971391	2.950805
C	3.114071	-2.064789	3.819581
C	3.838282	-1.277507	0.704727
C	4.125717	-1.848963	-0.581324
C	5.530043	-1.836007	-0.776206
C	6.136031	-1.288412	0.391305
C	5.104033	-0.937688	1.298719
Fe	4.826734	0.045194	-0.431114
C	5.701932	1.432536	-1.643032
C	4.426146	1.042848	-2.130597
C	3.444513	1.324662	-1.122540
C	4.142290	1.901604	-0.013750
C	5.526519	1.956013	-0.330730
P	1.678949	0.952357	-1.408341
C	1.070931	2.689290	-1.913668
C	-0.334571	2.545893	-2.514041
C	1.070015	3.726283	-0.789332
C	2.021551	3.156772	-3.031891
C	0.728859	-1.313016	3.754864
C	1.426925	-3.383207	2.516790
C	-1.177093	-3.656101	-0.595515
C	0.746817	-2.525921	-1.712019
C	-0.923138	3.342268	2.490621
C	-1.955726	1.316941	3.534568
H	3.385500	-2.177542	-1.297531
H	6.050115	-2.166162	-1.665402
H	7.195845	-1.136259	0.544796
H	5.228238	-0.462015	2.261899
H	3.695836	2.187340	0.930022
H	6.312530	2.303907	0.326256

H	6.644151	1.319504	-2.162144
H	4.222602	0.581705	-3.088271
H	2.902526	-2.696986	4.692520
H	3.425455	-1.077565	4.175579
H	3.949171	-2.517177	3.276195
H	0.992048	-0.291479	4.049408

6

G(ωB97XD/6-31+G*)= -5926.159483
E(ωB97XD/6-31+G*)= -5926.9024271

C	-5.531031	-0.113444	-2.159528
C	-5.980849	-1.230618	-1.397545
C	-4.837523	-1.880542	-0.857066
C	-3.665745	-1.167715	-1.273728
C	-4.111303	-0.070589	-2.085073
Fe	-4.897499	0.058463	-0.225568
C	-4.959254	0.089969	1.809296
C	-3.781860	0.749794	1.322592
C	-4.222026	1.831184	0.490992
C	-5.644719	1.831956	0.470027
C	-6.100954	0.754661	1.284593
P	-2.105420	0.187007	1.789059
C	-1.526570	1.671511	2.827761
C	-1.357986	2.994575	2.080838
P	-1.973407	-1.719440	-0.843706
C	-1.322334	-2.167521	-2.583755
C	-0.117656	-3.106226	-2.410232
P	-1.168553	0.316637	-0.260274
Si	1.082140	0.015455	-0.068396
P	2.067971	2.074809	-0.082223
C	1.416470	3.106676	-1.554925
C	0.180047	3.884290	-1.079609
C	3.714891	1.569552	-0.711364
C	4.072850	0.824762	-1.885798
C	5.491140	0.796846	-1.991270
C	6.028983	1.507801	-0.879783
C	4.941439	1.984353	-0.098126
Fe	4.863768	-0.054850	-0.243994
C	5.524812	-1.985972	-0.324086
C	4.105795	-1.938858	-0.228337
C	3.754192	-1.249797	0.979764
C	4.983108	-0.878345	1.618467
C	6.067230	-1.328198	0.818180
P	2.132166	-0.902656	1.749373
C	1.489127	-2.651123	2.119270

G(ωB97XD/6-31G*)= -5926.092315
E(ωB97XD/6-31G*)= -5926.839987

C	-5.604062	1.830588	0.424404
C	-6.104510	0.745398	1.198131
C	-4.993661	0.051709	1.748073
C	-3.791005	0.698483	1.314156
C	-4.184865	1.804785	0.492999
Fe	-4.867376	0.068589	-0.271345

C	-4.055962	-0.036836	-2.109117
C	-3.640007	-1.153045	-1.310034
C	-4.828444	-1.851575	-0.920492
C	-5.951836	-1.174936	-1.465754
C	-5.474034	-0.055864	-2.204391
P	-1.966185	-1.739220	-0.855451
C	-1.274144	-2.152922	-2.587941
C	-0.883742	-0.956652	-3.459056
P	-2.137389	0.107637	1.821959
C	-1.561925	1.562069	2.904187
C	-0.341043	1.093402	3.706826
P	-1.170219	0.277106	-0.210375
Si	1.082768	0.019776	-0.037889
P	2.165621	-0.900300	1.751811
C	1.535917	-2.652721	2.121250
C	2.681063	-3.374388	2.851238
C	3.767454	-1.234386	0.936918
C	4.077871	-1.897552	-0.295880
C	5.490862	-1.942364	-0.440526
C	6.071348	-1.307581	0.693834
C	5.016831	-0.876269	1.540045
Fe	4.836761	-0.025465	-0.291757
C	6.000380	1.539715	-0.871495
C	4.917459	1.993978	-0.073347
C	3.688796	1.604155	-0.696239
C	4.041574	0.897056	-1.894504
C	5.458223	0.868364	-2.003398
P	2.048611	2.085785	-0.034735
C	1.351458	3.137365	-1.467481

TS of the inversion of the central P in **6**

E(ωB97XD/6-31G*)= -5926.803696

C	6.071692	1.583749	0.865568
C	5.479620	1.180590	2.090776
C	4.071431	1.247309	1.934772
C	3.766574	1.692225	0.604943
C	5.025679	1.897696	-0.044981
Fe	4.884275	-0.032286	0.571325
C	4.459563	-1.963588	1.077220
C	3.771296	-1.631699	-0.135361
C	4.778701	-1.217414	-1.068293
C	6.052283	-1.333976	-0.448219
C	5.853775	-1.786894	0.885049
P	1.964204	-1.945218	-0.267342
C	1.474129	-2.460713	-2.047905
C	2.650709	-2.554154	-3.024969
P	2.056822	2.103938	0.067487
C	2.126595	2.403925	-1.800909
C	2.756532	3.791196	-2.016244
Si	1.178531	0.045339	0.463735
P	-1.038137	0.084060	0.435509
P	-1.931750	2.033171	0.282521
C	-2.577236	2.667981	1.941441
C	-3.063882	4.103957	1.671138

C	-3.369649	1.629047	-0.776318
C	-4.752772	1.976648	-0.665534
C	-5.425422	1.559184	-1.847181
C	-4.468148	0.954521	-2.705494
C	-3.209069	0.996446	-2.052922
Fe	-4.545885	-0.026120	-0.929160
C	-5.972935	-1.108020	0.038418
C	-4.795889	-1.006450	0.825975
C	-3.702765	-1.577043	0.092863
C	-4.239748	-2.021927	-1.159097
C	-5.627365	-1.730906	-1.193044
P	-1.956259	-1.892916	0.525795
C	-1.975812	-2.530742	2.309762
C	-0.520660	-2.921435	2.615344
C	-2.460949	-1.572292	3.394175
C	-2.851977	-3.793953	2.288237
C	-3.672771	1.856475	2.629758
C	-1.343013	2.724857	2.856200
C	0.885756	-3.874301	-1.858819
C	0.382492	-1.567767	-2.652509
C	2.886915	1.347468	-2.607918
C	0.670443	2.456841	-2.291130
H	-5.229145	2.458820	0.175329
H	-6.485364	1.660807	-2.038282
H	-4.667263	0.505172	-3.669052
H	-2.282167	0.585075	-2.430723
H	-4.739463	-0.549911	1.801235
H	-6.952892	-0.742729	0.315427
H	-6.298739	-1.925496	-2.018656
H	-3.663639	-2.487329	-1.948206
H	5.180296	2.229973	-1.060166
H	7.130459	1.622306	0.646865
H	6.003638	0.853256	2.978603
H	3.346654	0.999923	2.697336

TS of the inversion of the outer P of the P_3 unit in **6**

E(ωB97XD/6-31G*)= -5926.7923125

C	0.123237	3.540098	-1.661620
P	2.186971	-0.383608	1.894839
C	1.465857	-1.911645	2.746145
C	2.482272	-2.303654	3.830834
C	2.419932	3.806668	-2.623484
C	1.052252	1.796830	-3.213339
Si	1.140496	0.058766	-0.083303
P	-1.083060	0.162113	-0.481068
P	-2.085715	1.222506	1.089671
C	-3.847194	1.307766	0.762771
C	-4.859928	0.638802	1.533606
C	-6.122477	0.907586	0.944040
C	-5.908269	1.727503	-0.201225
C	-4.516196	1.983694	-0.306614
Fe	-4.871374	-0.016668	-0.373503
C	-4.778692	-2.043680	-0.344660
C	-5.882944	-1.611441	-1.127146

C	-5.380889	-0.791777	-2.176720
C	-3.968856	-0.713880	-2.037489
C	-3.577879	-1.498823	-0.900749
P	-1.909288	-1.942839	-0.272752
C	-1.257225	-2.895482	-1.806604
C	-2.348451	-3.911506	-2.186115
C	-0.916487	-2.037596	-3.031071
C	-0.004850	-3.681478	-1.389064
C	1.195050	-3.112497	1.843225
C	0.152443	-1.469334	3.407079
C	-1.621834	2.188999	2.641971
C	-2.264267	3.577091	2.488710
C	-0.106347	2.336335	2.745096
C	-2.180222	1.502875	3.896822
H	5.123590	2.600292	0.156153
H	7.108605	1.292500	-1.120705
H	5.979087	-0.432485	-2.855781
H	3.312804	-0.215081	-2.631995
H	3.304019	-2.475393	-0.417932
H	5.967210	-2.692915	-0.684064
H	7.126112	-1.013067	1.075485
H	5.163038	0.236337	2.442551
H	0.639877	2.339256	-4.074878
H	0.298934	1.070046	-2.893028
H	1.940190	1.260645	-3.560354
H	-0.639040	2.863241	-1.260365
H	-0.313792	4.056831	-2.525983
H	0.348146	4.291073	-0.896075
H	2.693192	4.539421	-1.855783
H	1.990637	4.352580	-3.473371
H	3.335472	3.313407	-2.966525
H	-0.292069	-2.314591	3.947653
H	-0.584426	-1.131769	2.667406
H	0.320633	-0.660809	4.127257
H	0.398654	-2.889998	1.130319
H	0.858562	-3.965233	2.448419
H	2.089080	-3.426150	1.296041
H	3.421281	-2.656403	3.390839
H	2.069259	-3.116724	4.441437
H	2.710657	-1.463903	4.496738
H	-4.823809	-2.669309	0.537094

TS of the inversion of the outer P in the PsiP unit in **6**

E(ωB97XD/6-31+G*)= -5926.811262

C	5.376775	-1.911547	1.343428
C	5.823475	-0.713224	1.968711
C	4.691839	0.121139	2.173560
C	3.529508	-0.552093	1.674570
C	3.971637	-1.813877	1.156503
Fe	4.900853	-0.300483	0.202008
C	4.393997	-0.519634	-1.736813
C	3.972075	0.758719	-1.244535
C	5.149294	1.443520	-0.798111
C	6.271448	0.599193	-1.008243

C	5.804489	-0.613514	-1.590230
P	2.314558	1.517990	-1.125995
C	1.858935	1.706098	-2.961754
C	1.614467	0.402213	-3.725412
P	1.849525	0.169964	1.786299
C	1.016202	-1.059365	2.969588
C	-0.365056	-0.493314	3.328579
P	1.246898	-0.304781	-0.330974
Si	-0.973532	0.044582	-0.618513
P	-2.041103	1.634851	0.450299
C	-1.477927	3.293786	1.122503
C	-2.289988	4.407673	0.444199
C	-3.791459	1.380843	0.778702
C	-4.885957	1.935889	0.032713
C	-6.099772	1.468839	0.599045
C	-5.776641	0.620161	1.697089
C	-4.363987	0.572649	1.816702
Fe	-4.928172	-0.080207	-0.017266
C	-6.024028	-1.622988	-0.775615
C	-4.882321	-2.099901	-0.080079
C	-3.709519	-1.542811	-0.686103
C	-4.155838	-0.720760	-1.772306
C	-5.574438	-0.766914	-1.820308
P	-2.024890	-1.915720	-0.083326
C	-1.452941	-3.199774	-1.379139
C	-2.540530	-4.285498	-1.422956
C	-1.243855	-2.643759	-2.790578
C	-0.147154	-3.832641	-0.876130
C	0.007753	3.486333	0.824557
C	-1.723980	3.326607	2.637399
C	3.040440	2.451003	-3.608518
C	0.620190	2.614910	-3.034874
C	1.893366	-1.054081	4.234786
C	0.871263	-2.493389	2.459522
H	-4.883899	-2.751107	0.784203
H	-7.055480	-1.842880	-0.534776
H	-6.204165	-0.217492	-2.507360
H	-3.520692	-0.125239	-2.415144
H	-4.783624	2.573217	-0.835361
H	-7.096282	1.689112	0.239828
H	-6.485197	0.082849	2.313329
H	-3.795615	-0.005070	2.533436
H	-1.037719	-3.468411	-3.486009
H	-0.390127	-1.960614	-2.831327
H	-2.130388	-2.114116	-3.154343
H	0.658483	-3.095180	-0.793398

7

G(ωB97XD/6-31+G*)= -5874.902681
E(ωB97XD/6-31+G*)= -5875.653508

C	4.343836	-1.947903	0.265095
C	3.917096	-1.007067	1.237723
C	5.111513	-0.411347	1.790389
C	6.233849	-1.001207	1.163654

C	5.770082	-1.943260	0.219645
P	2.258905	-0.503723	1.818086
Si	1.198688	-0.081429	-0.153416
P	2.199152	1.918052	-0.563154
C	1.560885	2.663581	-2.189640
C	2.666867	3.614745	-2.665362
Fe	5.020227	-0.103627	-0.223245
C	4.214683	0.308466	-2.050310
C	3.833671	1.313278	-1.102032
C	5.052304	1.908092	-0.611540
C	6.150048	1.264578	-1.247589
C	5.639180	0.283334	-2.127642
C	0.321018	3.498663	-1.846835
C	1.217734	1.657116	-3.285010
Si	-1.180021	0.057031	-0.135781
P	-2.260051	0.633233	1.789136
C	-1.668941	2.307298	2.444949
C	-2.642507	2.665430	3.561922
C	-1.633881	3.433196	1.426887
C	-0.263942	2.117041	3.018499
P	-2.160201	-1.982881	-0.416614
C	-1.552077	-2.755104	-2.042195
C	-2.663019	-3.701549	-2.514288
C	-1.192963	-1.792620	-3.157762
C	-0.315148	-3.599571	-1.691037
C	-3.833073	-1.453587	-0.924483
C	-4.250063	-0.603737	-1.994094
C	-5.664955	-0.612523	-2.068893
C	-6.152743	-1.454475	-1.030755
C	-5.023954	-1.979912	-0.325415
Fe	-5.010330	0.064000	-0.245959
C	-5.117230	0.642930	1.712448
C	-3.887943	1.091797	1.111389
C	-4.252219	1.930020	-0.003129
C	-5.670709	1.986513	-0.078053
C	-6.205834	1.193231	0.968389
C	1.599788	-2.108657	2.581602
C	2.450868	-2.335593	3.830237
C	0.153339	-1.853811	2.987349
C	1.661835	-3.334631	1.699324
H	-3.670884	-0.110172	-2.563697
H	-6.194775	-0.139484	-2.699036
H	-7.065776	-1.635637	-0.838943
H	-5.058011	-2.575079	0.414469
H	-3.646797	2.369606	-0.588009
H	-6.173405	2.473173	-0.719962
H	-7.129102	1.051566	1.142883
H	-5.191892	0.078510	2.472671
H	-0.905255	-2.299210	-3.946645
H	-0.464293	-1.207604	-2.861094
H	-1.975930	-1.248165	-3.385418
H	-0.001241	-4.068122	-2.492692

TS of the inversion of the P in **7**

E(ωB97XD/6-31G*)= -5875.561082

C	-3.758804	1.494124	0.866664
C	-4.939928	2.106563	0.335666
C	-6.069448	1.597613	1.029309
C	-5.603665	0.664305	1.997266
C	-4.187619	0.601889	1.903609
Fe	-5.023359	0.095453	0.132698
C	-4.599948	-0.405953	-1.782301
C	-5.998243	-0.485961	-1.559570
C	-6.225671	-1.420244	-0.507743
C	-4.967972	-1.915731	-0.079135
C	-3.941363	-1.287853	-0.860943
P	-2.168846	-1.530416	-0.695415
C	-1.594807	-3.000125	-1.708123
C	-2.356329	-4.247317	-1.233842
P	-2.081199	1.907443	0.260711
C	-1.465144	3.040846	1.672850
C	-0.192368	3.748259	1.185281
Si	-1.073819	-0.123439	0.569349
Si	1.249123	-0.017522	0.197779
P	2.055830	0.084298	-1.924805
C	1.188149	1.450234	-2.907943
C	-0.133000	0.852269	-3.413728
P	2.435305	-1.835778	0.886011
C	1.874529	-2.284869	2.642479
C	0.610265	-3.146732	2.493519
C	4.045216	-1.009487	1.166706
C	5.274428	-1.536520	0.653381
C	6.339233	-0.688786	1.057877
C	5.784883	0.372230	1.827558
C	4.378195	0.182374	1.891498
Fe	4.968529	0.350510	-0.033095
C	3.685531	0.825657	-1.543200
C	4.030153	1.982270	-0.767923
C	5.439033	2.159517	-0.823146
C	5.984437	1.115279	-1.622150
C	4.910989	0.298052	-2.064264
C	0.907042	2.742091	-2.140470
C	2.098377	1.756914	-4.108493
C	2.995144	-3.145685	3.247269
C	1.592952	-1.101831	3.574088
C	-0.093449	-3.202428	-1.504641
C	-1.900511	-2.743221	-3.190290
C	-2.554876	4.102308	1.895287
C	-1.181540	2.327832	2.998535
H	7.383949	-0.813988	0.806760
H	0.793105	-0.460021	3.191676
H	2.338601	0.850628	-4.676098
H	1.653731	1.172903	1.003814
H	2.674400	-3.535391	4.222034
H	3.038288	2.221225	-3.792124
H	-1.186478	-0.472102	2.019149
H	-7.188692	-1.680671	-0.088950
H	-4.093363	0.233535	-2.492795
H	-6.221882	0.077586	2.663597

H	-3.543735	-0.042096	2.488042
H	-7.103652	1.846792	0.833014
H	-4.955770	2.817580	-0.479916
H	-4.790598	-2.622150	0.720678
H	-6.759178	0.085029	-2.074928
H	-0.930854	3.068214	3.769972
H	-0.332645	1.640204	2.919107
H	-2.050596	1.761737	3.348514
H	0.153495	4.454010	1.951691
H	-0.375019	4.311437	0.263995
H	0.622484	3.042467	0.996279
H	-2.826040	4.606215	0.960237
H	-2.181358	4.864185	2.591686
H	-3.462911	3.668779	2.324416
H	-2.134927	-4.468003	-0.185013
H	-2.060208	-5.112623	-1.842097
H	-3.438923	-4.118976	-1.338199
H	0.232491	-4.080381	-2.076098
H	0.153466	-3.383333	-0.454066
H	0.493254	-2.348851	-1.858851
H	-1.341382	-1.880911	-3.563305
H	-2.967916	-2.556049	-3.349853
H	-1.619764	-3.623250	-3.784544
H	5.357809	-2.427059	0.044652
H	6.333410	1.198328	2.260258
H	3.675208	0.847645	2.373631
H	4.989584	-0.589858	-2.677450
H	7.033723	0.957956	-1.832779
H	6.001085	2.934906	-0.319741
H	3.340416	2.594835	-0.203355
H	-0.218277	-2.599481	2.030194
H	0.269097	-3.478751	3.482651
H	0.808099	-4.039072	1.889374
H	1.273539	-1.472436	4.557445
H	2.486883	-0.491011	3.727443
H	3.910809	-2.564933	3.399310
H	3.236853	-4.000788	2.605812
H	-0.661783	1.589990	-4.030670
H	-0.802415	0.568067	-2.593351
H	0.047561	-0.036484	-4.028954
H	0.206832	2.572095	-1.318583
H	0.443018	3.478522	-2.810349
H	1.822157	3.188443	-1.741021
H	1.586749	2.453281	-4.784999

8

G(ωB97XD/6-31+G*)= -5836.731023
E(ωB97XD/6-31+G*)= -5837.442745

C	4.990618	-1.889647	1.324535
C	5.445549	-1.654017	-0.005054
C	4.302343	-1.447609	-0.827425
C	3.125650	-1.576242	-0.017861
C	3.569018	-1.847804	1.318604
Fe	4.332318	-0.069169	0.645147
C	3.408765	1.403156	1.723111

C	3.112218	1.521295	0.324019
C	4.369264	1.685308	-0.350912
C	5.411678	1.670236	0.619066
C	4.817992	1.484590	1.897179
N	1.826927	1.359793	-0.294142
Si	1.678193	2.134070	-1.895392
C	2.709657	1.245074	-3.213726
N	1.778056	-1.380375	-0.466103
Si	1.161732	-2.543076	-1.671038
C	-0.027802	-3.777888	-0.898372
P	1.077141	-0.041679	0.439872
P	-1.052507	-0.032571	-0.325240
N	-1.829871	1.406122	0.303489
Si	-1.454525	2.779990	1.371642
C	-2.792818	3.071401	2.672290
C	-3.129355	1.483781	-0.304079
C	-3.433382	1.422673	-1.705197
C	-4.846168	1.428221	-1.863131
C	-5.435481	1.501056	-0.568846
C	-4.384586	1.522963	0.392115
Fe	-4.259836	-0.164083	-0.704374
C	-4.711063	-1.989789	-1.528326
C	-3.305743	-1.864199	-1.349488
C	-3.042424	-1.618198	0.036842
C	-4.312185	-1.598358	0.705194
C	-5.333930	-1.842734	-0.256699
N	-1.767629	-1.322079	0.616997
Si	-1.522884	-1.920434	2.280612
C	-2.146381	-0.664690	3.537663
C	-2.440583	-3.558185	2.425733
C	0.269402	-2.309539	2.678996
C	0.154363	2.584697	2.310830
C	-1.387110	4.332475	0.310530
C	-0.074084	2.182765	-2.551846
C	2.267857	3.909427	-1.684413
C	0.351656	-1.704749	-3.148559
C	2.594138	-3.564053	-2.343300
H	-4.510547	1.510283	1.464504
H	-6.494731	1.507735	-0.347984
H	-5.376327	1.370610	-2.804667
H	-2.708579	1.330591	-2.500734
H	-4.476633	-1.393339	1.753808
H	-6.396593	-1.877101	-0.056013
H	-5.214556	-2.159054	-2.470954
H	-2.560911	-1.898467	-2.132088
H	4.520682	1.757926	-1.417126
H	6.469351	1.758974	0.409131
H	5.340823	1.403154	2.840939
H	2.688338	1.216064	2.505599
H	4.315419	-1.205815	-1.880944
H	6.476567	-1.609377	-0.330370
H	5.614541	-2.055388	2.193027
H	2.929880	-1.948276	2.183548
H	2.029880	4.486424	-2.586982
H	1.765728	4.390194	-0.836924

H	3.345677	3.985438	-1.512130
H	-0.052904	2.834951	-3.435818
H	-0.422620	1.198148	-2.875593
H	-0.799911	2.595584	-1.848531
H	2.160397	1.207593	-4.162372
H	3.668406	1.737175	-3.412411
H	2.916293	0.214654	-2.907556
H	0.046089	-2.481172	-3.862592
H	-0.533537	-1.122256	-2.880539
H	1.052730	-1.039920	-3.664668
H	-0.329666	-4.518454	-1.650122
H	0.461933	-4.318761	-0.078820
H	-0.929499	-3.306468	-0.502107
H	2.162251	-4.373989	-2.945887
H	3.262364	-2.991690	-2.994586
H	3.200484	-4.020675	-1.554189
H	-2.139866	-4.048016	3.360462
H	-3.529831	-3.471830	2.427821
H	-2.161882	-4.223230	1.599335
H	-2.078418	-1.067173	4.556284
H	-1.539421	0.247023	3.500389
H	-3.186978	-0.374195	3.356412
H	0.279111	-3.005825	3.527889
H	0.776841	-2.796855	1.840616
H	0.842013	-1.421468	2.958437
H	-2.401203	3.796079	3.398366
H	-3.706296	3.499029	2.247372
H	-3.055994	2.163334	3.225449
H	-1.343944	5.228536	0.942362
H	-0.511428	4.344033	-0.347004
H	-2.281414	4.412023	-0.319588
H	0.169810	3.360075	3.087631
H	0.258647	1.615556	2.809948
H	1.025342	2.735481	1.670282

G(ωB97XD/6-31+G*)= -5482.732423
E(ωB97XD/6-31+G*)= -5483.535240

C	-4.850979	-1.497746	-1.267593
C	-3.876332	-1.445172	-2.301334
C	-2.591376	-1.417809	-1.691769
C	-2.753759	-1.433938	-0.270670
C	-4.167242	-1.487947	-0.018249
Fe	-3.694311	0.175228	-1.064084
C	-2.556575	1.814777	-1.516746
C	-2.675855	1.637473	-0.103715
C	-4.077217	1.689797	0.208464
C	-4.800788	1.891444	-1.001755
C	-3.859783	1.954558	-2.068134
N	-1.621279	1.412917	0.818844
C	-1.832741	1.978948	2.162718
C	-1.547498	3.488817	2.344490
C	-2.130480	3.883893	3.709644
N	-1.718092	-1.356437	0.702879
C	-2.110191	-1.806827	2.053630

C	-1.989600	-3.320247	2.342161
C	-2.678277	-3.571625	3.691560
P	-0.618218	-0.002512	0.804601
P	0.390563	0.122729	-1.240091
P	2.033768	-1.306924	-1.600207
C	1.290733	-2.941223	-2.184164
C	0.338175	-3.604774	-1.190901
C	2.873926	-1.711971	-0.043837
C	2.424841	-1.769744	1.315536
C	3.558785	-1.940021	2.155257
C	4.718705	-2.002261	1.330893
C	4.302449	-1.862998	-0.019894
Fe	3.727728	-0.226332	1.034117
C	5.080118	1.289598	1.228443
C	4.356801	1.411711	0.007801
C	2.959313	1.539215	0.312350
C	2.848640	1.480199	1.741564
C	4.144179	1.323187	2.301196
P	1.485731	1.965541	-0.699934
C	2.101914	2.680498	-2.339372
C	2.951861	3.909333	-1.968278
C	0.843014	3.164034	-3.081972
C	2.890488	1.755068	-3.271953
C	2.489311	-3.878202	-2.423115
C	0.602490	-2.681504	-3.532487
C	-2.195279	4.344508	1.249118
C	-0.035640	3.738607	2.349349
C	-2.656510	-4.181176	1.262896
C	-0.508350	-3.696203	2.453664
H	4.796357	1.387570	-0.979021
H	6.149621	1.150377	1.318590
H	4.373534	1.213329	3.352986
H	1.917037	1.515412	2.290509
H	4.947738	-1.827871	-0.888693
H	5.740303	-2.096065	1.675697
H	3.544756	-1.976234	3.236980
H	1.407660	-1.632933	1.652192
H	3.764923	1.309919	-2.791938
H	3.236193	2.329870	-4.142593

E(ωB97XD/6-31G*)= -5483.469665

C	4.213262	1.303170	2.253759
C	5.113987	1.230273	1.155236
C	4.358313	1.353095	-0.043785
C	2.975650	1.522607	0.299967
C	2.906190	1.484906	1.731489
Fe	3.721545	-0.238113	1.025578
C	4.277251	-1.884860	0.005747
C	2.852942	-1.708104	-0.041450
C	2.384877	-1.736825	1.311002
C	3.501739	-1.910864	2.169277
C	4.670889	-2.009650	1.363982
P	2.031306	-1.296743	-1.605866
C	1.288434	-2.924536	-2.202763

C	0.597309	-2.653050	-3.546718
P	1.488519	1.968178	-0.680602
C	2.083140	2.704111	-2.317148
C	2.832912	1.780788	-3.282717
P	0.390719	0.133810	-1.235704
P	-0.619817	0.000192	0.806286
N	-1.712136	-1.358370	0.700672
C	-2.096704	-1.819772	2.050079
C	-1.964698	-3.333839	2.325625
C	-0.480528	-3.700599	2.423599
C	-2.752354	-1.426764	-0.268759
C	-2.594692	-1.397006	-1.688962
C	-3.880933	-1.418772	-2.293675
C	-4.851111	-1.484722	-1.258173
C	-4.163493	-1.487613	-0.011899
Fe	-3.697339	0.172521	-1.044471
C	-3.885048	1.931057	-2.045605
C	-2.577922	1.804878	-1.502707
C	-2.687144	1.627007	-0.090435
C	-4.085686	1.669616	0.231243
C	-4.817849	1.862825	-0.973871
N	-1.624124	1.411310	0.824201
C	-1.826128	1.980922	2.168053
C	-1.530214	3.488731	2.343395
C	-0.016524	3.725891	2.364606
C	-2.125193	3.898781	3.698749
C	-2.158744	4.338682	1.233121
C	-2.642270	-3.600716	3.677540
C	-2.634814	-4.188004	1.243507
C	0.339599	-3.597514	-1.212861
C	2.489985	-3.854693	-2.451902
C	2.963495	3.909094	-1.941923
C	0.815300	3.221458	-3.019954
H	4.767899	1.308408	-1.042439
H	6.182819	1.074353	1.215928
H	4.472530	1.213705	3.300040
H	1.993006	1.564046	2.305987
H	4.935174	-1.882263	-0.853566
H	5.684117	-2.127836	1.724110
H	3.469155	-1.940920	3.250192
H	1.365359	-1.584018	1.631358
H	3.716577	1.322749	-2.833030
H	3.158276	2.360368	-4.157343

9 (cis isomer)

G(ωB97XD/6-31G*)= -5482.664383
E(ωB97XD/6-31G*)=-5483.472604

C	-2.775526	1.309810	1.663699
C	-4.026254	1.226388	2.334600
C	-5.051368	1.156888	1.352643
C	-4.431877	1.180246	0.072200
C	-3.010209	1.271884	0.251737
Fe	-3.745846	-0.383043	1.129373
C	-2.420931	-1.853270	1.541636

C	-2.675602	-1.765656	0.140202
C	-4.083292	-1.974068	-0.044770
C	-4.678211	-2.183419	1.230651
C	-3.651685	-2.095812	2.211742
N	-1.735844	-1.480597	-0.881684
C	-1.955670	-2.182922	-2.156492
C	-1.396124	-3.619160	-2.256806
C	0.133386	-3.582975	-2.318356
N	-2.039245	1.289825	-0.789027
C	-2.501372	1.878289	-2.060023
C	-2.440821	3.418937	-2.168612
C	-0.982550	3.864981	-2.317322
P	-0.880212	0.012463	-1.024657
P	0.343446	0.217387	0.867349
P	1.361729	-1.740097	0.827505
C	2.005754	-2.017198	2.586012
C	0.785920	-1.987162	3.522462
C	2.810963	-1.610247	-0.279361
C	2.665930	-1.289981	-1.669858
C	3.920230	-1.456367	-2.313071
C	4.860128	-1.880520	-1.336051
C	4.183071	-1.977654	-0.088906
Fe	4.047092	-0.094145	-0.810725
C	5.173592	1.491655	-1.419235
C	3.789287	1.789470	-1.497693
C	3.206595	1.627148	-0.196824
C	4.271753	1.244428	0.683647
C	5.472690	1.158327	-0.068718
P	1.441325	1.987440	0.056212
C	1.445906	3.224639	1.492956
C	0.038490	3.840234	1.545164
C	1.803368	2.656292	2.868280
C	2.448996	4.327569	1.115469
C	2.574422	-3.449253	2.612720
C	3.045307	-1.019247	3.097433
C	-1.945486	-4.216934	-3.560451
C	-1.833530	-4.485647	-1.069829
C	-3.220790	3.806033	-3.434024
C	-3.071691	4.113694	-0.956369
H	5.873178	1.491120	-2.244307
H	-2.924252	-4.510714	-0.965312
H	4.653414	-2.253829	0.843118
H	5.912762	-2.069032	-1.498856
H	4.127224	-1.256765	-3.355684
H	1.753786	-0.942244	-2.138694
H	4.177703	1.030545	1.736458
H	6.439538	0.860857	0.315097
H	3.240014	2.059432	-2.390048
H	3.951060	-1.016693	2.486955
H	3.333694	-1.276845	4.125172
H	2.635384	-0.005240	3.112130
H	2.877686	-3.698361	3.637544
H	3.448704	-3.572407	1.968811
H	1.821967	-4.179211	2.296603
H	0.038706	-2.732791	3.230756

H	0.306654	-1.002521	3.542238
H	1.110547	-2.223631	4.543402
H	1.110044	1.862727	3.167363
H	1.748073	3.451141	3.623793
H	2.822045	2.258564	2.889540
H	2.374669	5.147745	1.840729
H	2.241514	4.740075	0.121308
H	3.479795	3.960496	1.122922
H	-0.206926	4.352575	0.609871
H	-0.012450	4.576965	2.356938
H	-0.736752	3.090546	1.727473
H	-1.461720	-1.706123	2.011016
H	-3.779670	-2.198552	3.280678
H	-5.728228	-2.363019	1.416959
H	-4.613528	-1.954903	-0.986634
H	-1.808376	1.314687	2.141753
H	-4.166102	1.215704	3.406991
H	-6.114548	1.087385	1.537941
H	-4.955951	1.120100	-0.870617
H	-1.493237	-1.584538	-2.949749
H	-3.030174	-2.206446	-2.378674
H	0.546129	-3.157990	-1.399902
H	0.484272	-2.982560	-3.167021
H	0.541681	-4.594121	-2.432966
H	-1.677614	-3.599241	-4.426966
H	-3.038548	-4.303901	-3.530141
H	-1.533927	-5.218868	-3.726012
H	-1.412001	-4.108568	-0.133039
H	-1.482062	-5.514973	-1.209673
H	-3.522837	1.541777	-2.278659
H	-1.872398	1.467670	-2.857500
H	-0.542847	3.466704	-3.240401
H	-0.373566	3.502051	-1.485808
H	-0.910929	4.958873	-2.354781
H	-4.283750	3.552278	-3.340338
H	-2.824997	3.292703	-4.319155
H	-3.148148	4.884428	-3.614632
H	-3.032958	5.201478	-1.090232
H	-2.539299	3.867001	-0.033227
H	-4.120358	3.826347	-0.823428

TS of the inversion of the central P in the P_3 unit in **9**

E(ωB97XD/6-31G*)= -5483.443202

C	3.062493	0.481798	-0.960075
C	2.321785	-0.119518	-2.017639
C	3.217251	-0.867465	-2.830658
C	4.525939	-0.707126	-2.304060
C	4.438354	0.125826	-1.153302
Fe	3.300473	-1.507546	-0.893170
C	3.814824	-2.097840	0.958382
C	2.399590	-1.888134	0.862180
C	1.904737	-2.781682	-0.131250
C	2.998238	-3.509682	-0.667069
C	4.180773	-3.096586	0.013555

N	1.664381	-0.943099	1.623754
C	1.649012	-1.160886	3.078176
C	0.416324	-1.891807	3.653182
C	-0.824164	-0.991282	3.593599
N	2.506058	1.209645	0.121589
C	3.497209	1.846677	1.010228
C	3.968668	3.266854	0.615476
C	5.229373	3.564232	1.439707
P	1.172899	0.607987	1.076773
P	-0.675057	0.199210	-0.082818
P	-1.423728	-1.736443	-0.771267
C	-3.204757	-1.455654	-0.382968
C	-4.336170	-1.164361	-1.215832
C	-5.519908	-1.288579	-0.436893
C	-5.137174	-1.624392	0.891523
C	-3.721051	-1.724027	0.924558
Fe	-4.299025	0.135537	0.324954
C	-4.571957	1.978995	-0.458520
C	-5.462391	1.780245	0.629935
C	-4.682972	1.505683	1.789107
C	-3.313337	1.533127	1.418910
C	-3.230557	1.823832	0.018606
P	-1.801070	1.800244	-1.114681
C	-0.968283	3.457261	-0.784403
C	-0.728454	3.777462	0.692865
C	-1.374139	-2.055215	-2.652706
C	-1.567799	-0.829566	-3.547336
C	2.876870	4.278158	0.982348
C	4.290802	3.396329	-0.877916
C	0.147051	-3.188090	2.882390
C	0.735575	-2.215126	5.120181
C	-0.005192	-2.690214	-2.953228
C	-2.433617	-3.130546	-2.962124
C	-1.940310	4.501959	-1.365004
C	0.333273	3.477101	-1.597774
H	-2.512970	-0.315215	-3.357906
H	-5.804311	-1.760037	1.732168
H	-4.305748	-0.874074	-2.255894
H	0.791794	4.472896	-1.545367
H	-1.670163	3.772729	1.250736
H	2.940019	-4.254380	-1.449359
H	2.546375	-1.740758	3.327090
H	5.275523	0.404614	-0.531028
H	3.049723	1.923623	2.008635
H	5.433906	-1.142331	-2.698471

TS of the inversion of the P in the NPN unit in **9**

E(ωB97XD/6-31G*)= -5483.414089

C	-3.493720	0.804075	-2.796706
C	-4.640815	0.349471	-2.092014
C	-4.347133	0.396382	-0.699042
C	-3.015815	0.896728	-0.534753
C	-2.498592	1.146247	-1.839754

Fe	-3.028078	-0.801752	-1.639016
C	-3.126506	-2.508446	-0.593529
C	-1.797637	-1.974673	-0.532069
C	-1.316756	-1.889392	-1.870894
C	-2.333012	-2.359166	-2.747776
C	-3.446693	-2.758270	-1.958242
N	-1.098787	-1.561117	0.643328
C	-1.510198	-2.257907	1.884819
C	-1.047918	-3.724080	2.089504
C	0.455364	-3.789919	2.385047
N	-2.300126	1.011621	0.690580
C	-3.096489	1.257855	1.902768
C	-3.378950	2.745880	2.192147
C	-4.112447	3.414296	1.022618
P	-0.842539	0.138107	0.801032
P	0.960439	1.215778	1.275581
P	2.081336	-0.625962	1.761680
C	3.518125	-0.060444	2.853509
C	4.374171	1.118564	2.382040
C	2.793285	-1.266709	0.193727
C	2.049991	-2.186820	-0.615579
C	2.893530	-2.682950	-1.643371
C	4.172948	-2.081808	-1.485350
C	4.111402	-1.202887	-0.368119
Fe	2.755675	-0.662169	-1.764779
C	1.736643	-0.207176	-3.473381
C	1.198540	0.498811	-2.365408
C	2.236774	1.287727	-1.784246
C	3.420594	1.069335	-2.562481
C	3.109592	0.148522	-3.598090
P	2.230188	2.237547	-0.245566
C	1.305322	3.828488	-0.666086
C	2.155626	4.546806	-1.727492
C	-0.106286	3.616965	-1.214138
C	1.265542	4.683852	0.609259
C	4.397491	-1.306351	3.062576
C	2.866706	0.310442	4.198249
C	-2.059259	3.478465	2.453288
C	-4.257972	2.803805	3.449257
C	-1.812321	-4.226335	3.326299
C	-1.354688	-4.655663	0.909424
H	-5.018810	0.085161	0.088223
H	-3.763164	2.326388	4.303767
H	-2.239914	4.534984	2.683452
H	3.794824	2.044751	2.337501
H	2.213860	3.969142	-2.656517
H	0.721488	-3.163439	3.243006
H	0.797080	5.651583	0.390149
H	-4.345229	4.457182	1.268544
H	4.924984	-0.583153	-0.020021
H	5.033680	-2.233319	-2.123063
H	2.604536	-3.379777	-2.418522
H	1.009549	-2.436736	-0.459633
H	4.388866	1.510454	-2.365835
H	3.801809	-0.235414	-4.335416

H	1.202036	-0.908182	-4.101162
H	0.197108	0.416541	-1.972752
H	4.807185	0.962032	1.391251
H	5.197535	1.270511	3.092866
H	5.147947	-1.093379	3.834174
H	4.926660	-1.597100	2.150896
H	3.805493	-2.165730	3.397821
H	0.688187	4.205228	1.406443
H	2.274419	4.874541	0.992116
H	3.175912	4.729150	-1.371688
H	1.701572	5.517888	-1.963151
H	-1.509344	1.509183	-2.062565
H	-3.389778	0.884278	-3.870051
H	-5.570438	0.011912	-2.529451
H	-0.342868	-1.525927	-2.158920
H	-2.261886	-2.411364	-3.825705
H	-4.379606	-3.163851	-2.325305
H	-3.784882	-2.688019	0.244220
H	-2.538283	0.839712	2.744865
H	-4.048726	0.712221	1.852170
H	-1.411550	3.430205	1.573626
H	-1.514022	3.032100	3.293352
H	-5.218494	2.299588	3.287609
H	-4.468626	3.843666	3.723152
H	-5.053892	2.901373	0.793861
H	-3.501363	3.411718	0.114251
H	-2.605465	-2.211082	1.986655
H	-1.097529	-1.670639	2.707963
H	1.049268	-3.447718	1.534798
H	0.747553	-4.823294	2.608095
H	-2.894307	-4.244603	3.147643
H	-1.622744	-3.588278	4.198023
H	-1.498692	-5.244368	3.583025
H	-1.020224	-5.671366	1.152614
H	-0.835687	-4.342612	-0.001720
H	-2.424954	-4.700975	0.686291
H	-0.585788	4.585874	-1.406831
H	-0.074286	3.071032	-2.162649
H	-0.742929	3.062880	-0.516745
H	3.646569	0.589514	4.917808
H	2.186337	1.163075	4.094959
H	2.301912	-0.529460	4.616826

TS of the inversion of the outer P in the P_3 unit in **9**

E(ωB97XD/6-31G*)= -5483.444159

H	2.946747	4.244939	3.171573
H	3.875237	3.162025	2.123539
H	1.941371	5.318620	1.208420
H	0.964647	4.410694	0.035650
H	2.728711	4.240356	0.041022
H	-0.442192	3.385236	1.821171
H	0.447832	4.286019	3.058145
H	0.291063	2.524222	3.192444
H	-1.775457	-1.435858	2.112805

H	-4.211981	-1.544135	3.230543
H	-6.039534	-1.661658	1.244298
H	-4.728660	-1.587821	-1.093116
H	-1.823646	1.691023	1.963766
H	-4.273089	1.920196	3.025422
H	-6.073662	1.777071	1.016154
H	-4.727250	1.448035	-1.279712
H	-1.403490	-1.749134	-2.819059
H	-3.090264	-1.997164	-2.409409
H	0.060432	-3.537123	-0.897545
H	0.270307	-3.554201	-2.662360
H	-0.118794	-5.050697	-1.800913
H	-1.755109	-3.774070	-4.193776
H	-3.351933	-4.114272	-3.498485
H	-2.070188	-5.334056	-3.417893
H	-2.242786	-4.047269	0.104901
H	-2.419029	-5.472709	-0.932106
H	-3.094841	2.142309	-2.448367
H	-1.613145	1.409905	-3.039966
H	0.438545	2.722191	-3.325439
H	0.534821	2.780328	-1.552558
H	0.634331	4.277425	-2.496788
H	-2.980812	4.240654	-3.607515
H	-1.676012	3.459330	-4.523415
H	-1.423725	5.061235	-3.812244
H	-1.329560	5.314893	-1.258310
H	-1.380851	3.872439	-0.225948
H	-2.844652	4.409555	-1.060113

TS of the inversion of the outer P in the P_3 unit in **9**

E(ωB97XD/6-31G*)= -5483.437087

C	-4.045533	1.591428	0.356466
C	-4.880061	1.762431	-0.783096
C	-4.042280	1.842986	-1.930081
C	-2.691319	1.741906	-1.498670
C	-2.678057	1.575871	-0.079682
Fe	-3.732387	0.095197	-0.944993
C	-4.117553	-1.576936	0.098880
C	-2.718255	-1.474966	-0.205174
C	-2.609622	-1.438459	-1.629062
C	-3.914361	-1.497653	-2.191282
C	-4.846468	-1.593404	-1.123817
N	-1.643997	-1.367847	0.718365
C	-1.989715	-1.730305	2.107091
C	-1.888939	-3.226108	2.483579
C	-2.533960	-3.378594	3.868650
N	-1.539079	1.399583	0.749333
C	-1.641081	1.999695	2.090972
C	-1.352613	3.512275	2.189845
C	-1.650066	3.929209	3.637729
P	-0.530411	-0.015338	0.705371
P	0.393463	0.125023	-1.384952
P	1.949407	-1.404106	-1.850347
C	1.165956	-3.083888	-2.221994

C	0.298570	-3.676397	-1.113368
P	1.809087	1.646942	-1.023976
C	3.068596	1.454560	0.239736
C	2.884780	1.300953	1.651820
C	4.153958	1.090573	2.250075
C	5.140390	1.127516	1.222415
C	4.477960	1.338535	-0.015473
Fe	3.778568	-0.340983	0.852132
C	3.629920	-2.112738	1.840714
C	4.764946	-2.116056	0.981594
C	4.309848	-1.881702	-0.343569
C	2.883485	-1.733275	-0.318383
C	2.475431	-1.881107	1.046310
C	1.750895	3.293904	-1.920823
C	0.296486	3.706959	-2.168322
C	2.480234	3.174056	-3.269193
C	2.462596	4.335553	-1.047067
C	-2.234810	4.334750	1.242896
C	0.123917	3.763923	1.875011
C	2.352328	-4.031201	-2.483724
C	0.371469	-2.932850	-3.527761
C	-2.604470	-4.143230	1.484816
C	-0.412498	-3.627762	2.567094
H	4.936996	1.381522	-0.993835
H	6.203844	0.983792	1.358847
H	4.339278	0.914069	3.301255
H	1.926462	1.298754	2.151463
H	4.927250	-1.801597	-1.228658
H	5.794818	-2.246147	1.286084
H	3.644302	-2.238170	2.915165
H	1.465816	-1.782025	1.416677
H	3.532050	2.907906	-3.125213
H	2.437053	4.132296	-3.805228

TS of the P-inversion of **11**

G(ωB97XD/6-31+G*)= -2988.659088
E(ωB97XD/6-31+G*)= -2989.011419

P	-0.041570	0.022824	0.039696
C	0.004108	-0.086612	3.484606
P	0.866275	0.018127	1.913297
C	2.720559	0.090984	2.132459
C	3.189127	-1.135006	2.931365
C	-0.560559	1.002547	4.233257
C	-1.236414	0.462492	5.358987
C	-1.094702	-0.956072	5.326777
C	-0.331941	-1.298311	4.179580
Fe	-2.003660	-0.289587	3.605275
C	-4.057307	-0.261122	3.645477
C	-3.553624	0.774130	2.810999
C	-2.761406	0.182090	1.774093
C	-2.792941	-1.235203	1.983276
C	-3.586406	-1.504035	3.132199
P	-1.861991	1.192967	0.537363
C	-2.910041	0.906337	-1.020248

C	-4.255689	1.604730	-0.762347
C	3.078053	1.374942	2.898932
C	3.378093	0.101189	0.747946
C	-3.163073	-0.564913	-1.367994
C	-2.195870	1.603971	-2.188562
H	-0.077394	-2.296769	3.849045
H	-1.527392	-1.652563	6.033080
H	-1.795973	1.027572	6.092998
H	-0.509542	2.045488	3.949033
H	-2.252260	-1.964101	1.393196
H	-3.764470	-2.478996	3.567647
H	-4.659370	-0.126206	4.534736
H	-3.696550	1.838015	2.953615
H	4.274095	-1.075345	3.090264
H	2.709390	-1.181470	3.915164
H	2.970367	-2.065196	2.396828
H	4.164594	1.421672	3.054064
H	2.771461	2.267043	2.343533
H	2.597751	1.398899	3.883457
H	4.467303	0.152369	0.870955
H	3.143107	-0.805931	0.180871
H	3.061016	0.964339	0.154277
H	-2.833344	1.575507	-3.082035
H	-1.981774	2.654894	-1.961332
H	-1.249352	1.112801	-2.441375
H	-3.759662	-0.628055	-2.288381
H	-2.228495	-1.111295	-1.537339
H	-3.719168	-1.074826	-0.574824
H	-4.886496	1.530420	-1.658109
H	-4.800112	1.141524	0.068437
H	-4.120201	2.668377	-0.533947

Cis isomer of **11**

G(ωB97XD/6-31+G*)= -2988.684119
E(ωB97XD/6-31+G*)= -2989.037431

C	-0.004169	0.036206	0.038166
C	-0.015695	0.036601	1.471955
C	1.353003	0.035368	1.901735
C	2.185600	0.030336	0.747700
C	1.346069	0.035708	-0.403947
Fe	0.973210	1.675400	0.773090
C	0.034129	3.370836	0.120438
C	0.021787	3.300018	1.552470
C	1.389913	3.248630	1.981005
C	2.223056	3.291451	0.828148
C	1.384271	3.362143	-0.321883
P	-1.531505	3.273809	2.516185
P	-1.323360	1.623805	3.933498
P	-1.568514	0.050894	2.436677
C	-1.492377	-1.553609	3.437405
C	-2.769774	-1.603101	4.290872
C	-0.264003	-1.713266	4.340603
C	-1.520604	-2.696938	2.409400

C	-1.419287	4.824885	3.594748
C	-0.188057	4.911810	4.503966
C	-2.695641	4.861337	4.450425
C	-1.420954	6.017861	2.624387
H	1.692278	0.083953	2.927710
H	3.267487	0.061182	0.744788
H	1.678936	0.070451	-1.433004
H	-0.885871	0.066630	-0.589101
H	1.727409	3.141731	3.003141
H	3.303951	3.235960	0.823102
H	1.716831	3.370551	-1.351590
H	-0.847686	3.391621	-0.507064
H	-1.542136	-3.661393	2.933617
H	-2.408550	-2.641828	1.769078
H	-0.632992	-2.684613	1.766575
H	-2.831721	-2.570632	4.805709
H	-2.777372	-0.818014	5.055708
H	-3.670426	-1.487365	3.677240
H	-0.350484	-2.644312	4.917085
H	0.658256	-1.774825	3.755660
H	-0.171435	-0.887032	5.054595
H	-2.735744	5.803440	5.012427
H	-3.598397	4.796612	3.832398
H	-2.721534	4.039834	5.175585
H	-0.253656	5.814984	5.125762
H	-0.114642	4.049447	5.176244
H	0.735657	4.981216	3.922212
H	-1.420813	6.955532	3.195536
H	-0.533518	6.017061	1.981205
H	-2.309590	6.014610	1.982660

trans isomer of **11**

G(ωB97XD/6-31+G*)= -2937.408491
E(ωB97XD/6-31+G*)= -2989.035018

C	-0.101408	-0.111773	-0.127432
C	-0.187098	-0.092433	1.290665
C	1.134205	0.013101	1.814517
C	2.037995	0.052416	0.718538
C	1.281047	-0.035605	-0.499440
Fe	0.960811	-1.668067	0.637435
C	-0.121503	-3.385384	0.620448
C	0.795429	-3.421681	1.705750
C	2.111011	-3.286834	1.174071
C	2.008823	-3.176154	-0.238705
C	0.621703	-3.221989	-0.597880
P	-0.190926	-2.948289	-2.204758
C	0.048465	-4.604826	-3.095591
C	1.512903	-5.041211	-3.211454
P	2.083172	-0.172362	-2.129098
C	1.727636	1.513434	-2.923208
C	2.434139	2.556216	-2.041457
P	0.993014	-1.566867	-3.416077
C	-0.579135	-4.485263	-4.491900
C	-0.730882	-5.644236	-2.272947

C	0.236079	1.852071	-3.017498
C	2.357793	1.518528	-4.323519
H	-1.201201	-3.420397	0.693241
H	0.538373	-3.501729	2.753860
H	3.027344	-3.240289	1.748140
H	2.829022	-3.014407	-0.926738
H	-0.929245	-0.235086	-0.814048
H	-1.096374	-0.180722	1.871029
H	1.403431	0.026514	2.862536
H	3.118254	0.089773	0.780320
H	-0.548947	-5.461350	-4.993064
H	-1.626524	-4.166501	-4.436652
H	-0.037632	-3.771659	-5.122942
H	-0.693118	-6.616174	-2.781798
H	-0.300323	-5.773541	-1.273823
H	-1.785105	-5.365228	-2.159903
H	1.568786	-6.017555	-3.711727
H	2.099909	-4.327477	-3.799747
H	1.980836	-5.143776	-2.226900
H	2.316749	3.552115	-2.488119
H	2.005288	2.589747	-1.033826
H	3.507619	2.352655	-1.951799
H	0.110286	2.842480	-3.475639
H	-0.303379	1.124353	-3.633849
H	-0.232470	1.882064	-2.028578
H	2.251750	2.516126	-4.769005
H	3.426491	1.277783	-4.285311
H	1.868542	0.802790	-4.993635

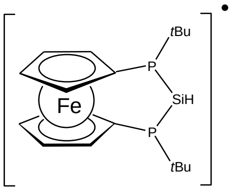

G(ωB97XD/6-31+G*)= -2937.408491
E(ωB97XD/6-31+G*)= -2937.768950

C	0.078604	-0.012562	-0.289150
C	0.587513	0.500405	0.950117
C	1.997440	0.310900	0.971357
C	2.375171	-0.329504	-0.244336
C	1.200302	-0.527037	-1.017991
Fe	1.029718	-1.478103	0.790987
C	-0.312737	-2.970961	1.224395
C	0.206895	-2.373691	2.420723
C	1.619636	-2.541440	2.431429
C	1.991327	-3.231331	1.241232
C	0.807848	-3.496164	0.501533
P	-2.029835	-3.124545	0.617807
Si	-2.757882	-0.994590	0.756998
P	-1.624088	-0.056005	-0.951604
C	-2.140496	1.762910	-1.062221

C	-3.594144	1.773255	-1.561163
C	-2.039941	2.557214	0.245102
C	-1.231133	2.411174	-2.119103
C	-2.919971	-4.130982	1.952815
C	-2.282016	-5.529921	1.942449
C	-4.389002	-4.234352	1.513025
C	-2.847221	-3.554017	3.371250
H	-3.910115	2.805586	-1.759825
H	-2.413492	3.578229	0.086700
H	-0.374120	-1.845409	3.164888
H	2.298276	-2.170108	3.188438
H	3.000629	-3.476121	0.937202
H	0.748775	-3.985939	-0.462023
H	-0.007794	0.918507	1.750880
H	2.661142	0.566823	1.787155
H	3.375175	-0.643756	-0.513162
H	1.141802	-1.009669	-1.985172
H	-4.930954	-4.909427	2.187982
H	-4.892432	-3.261072	1.544133
H	-4.479224	-4.629849	0.494989
H	-3.438725	-4.178469	4.054840
H	-1.819416	-3.540134	3.745562
H	-3.246341	-2.534063	3.418003
H	-2.795654	-6.176040	2.666851
H	-2.358797	-5.999875	0.955510
H	-1.222542	-5.489507	2.220492
H	-1.520311	3.460933	-2.262089
H	-0.179804	2.391529	-1.809777
H	-1.312475	1.902753	-3.086414
H	-2.634827	2.102546	1.045828
H	-1.003805	2.636015	0.587027
H	-3.707199	1.200568	-2.488625
H	-4.282308	1.353821	-0.818056
H	-4.203212	-1.010802	0.351425

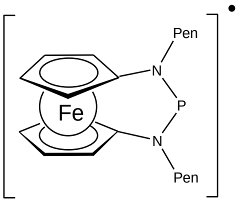

G(ωB97XD/6-31+G*)= -2494.009955
E(ωB97XD/6-31+G*)= -2494.427444

C	-1.675250	1.600557	-1.134761
C	-1.858273	2.895621	-0.570929
C	-1.848591	2.765950	0.848003
C	-1.641275	1.392422	1.164362
C	-1.526036	0.665192	-0.063876
Fe	-0.091490	2.073547	0.054023
C	1.526604	1.946399	-1.166014
C	1.535649	3.201163	-0.498472
C	1.551162	2.953593	0.906505
C	1.558798	1.544126	1.109013
C	1.528873	0.909735	-0.173359

N	1.484928	-0.484430	-0.439023
P	0.087738	-1.267400	-1.123540
N	-1.234362	-0.710677	-0.190266
C	-1.963835	-1.650860	0.663815
C	-3.368741	-2.056743	0.167024
C	-3.266135	-2.696225	-1.222017
C	2.756627	-1.087083	-0.861758
C	3.491975	-1.920815	0.210480
C	2.630991	-3.110804	0.649525
C	4.787568	-2.435230	-0.433820
C	3.837049	-1.062238	1.432189
C	-3.921913	-3.080252	1.169126
C	-4.308859	-0.844959	0.111214
H	-2.049094	-1.229517	1.675594
H	3.420542	-0.286762	-1.213794
H	2.568727	-1.740380	-1.727684
H	-1.352121	-2.556139	0.754076
H	-1.603899	1.357302	-2.186981
H	-1.971801	3.818444	-1.124338
H	-1.951831	3.570959	1.563529
H	-1.540636	0.976121	2.157868
H	1.473974	1.781346	-2.234762
H	1.505951	4.173681	-0.972079
H	1.537259	3.706006	1.683889
H	1.529569	1.033127	2.061496
H	-4.921303	-3.415195	0.867828
H	-3.277497	-3.966028	1.232784
H	-4.004693	-2.647748	2.174363
H	-4.251524	-3.032444	-1.566499
H	-2.878760	-1.980591	-1.955674
H	-2.596396	-3.565284	-1.211815
H	-5.322653	-1.163566	-0.161174
H	-4.364515	-0.338616	1.082748
H	-3.978359	-0.110365	-0.629255
H	3.171038	-3.723186	1.381998
H	1.698366	-2.771647	1.113001
H	2.369866	-3.751310	-0.201826
H	4.459847	-1.630577	2.133630
H	4.384759	-0.155862	1.146575
H	2.930511	-0.756752	1.962346
H	5.349876	-3.058468	0.271179
H	4.577042	-3.043516	-1.322438
H	5.438119	-1.605798	-0.738574

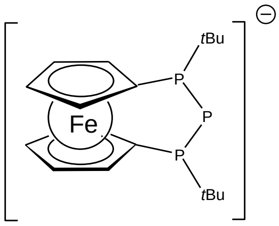

G(ωB97XD/6-31+G*)= -2988.456033
E(ωB97XD/6-31+G*)= -2988.809442

C	1.002966	-0.015145	0.007442
C	-0.004231	-0.010034	1.443225

C	1.341316	-0.011978	1.893404
C	2.191058	-0.024606	0.751555
C	1.376725	-0.033229	-0.410669
Fe	0.955509	1.622860	0.689862
C	0.456994	3.176300	-0.472711
C	-0.281204	3.234456	0.764046
C	0.663128	3.334167	1.815043
C	1.972003	3.334658	1.254947
C	1.860382	3.236837	-0.154560
P	-0.240092	2.923119	-2.083121
P	-1.290347	1.326180	-2.683182
P	-1.539187	0.003670	-0.952773
C	-1.616970	-1.687233	-1.796648
C	-1.742473	-2.691786	-0.635957
C	-0.389872	-2.034329	-2.646305
C	-2.892024	-1.725584	-2.652992
C	-0.130542	4.407410	-3.199300
C	-0.767631	4.087651	-4.555444
C	1.357023	4.764261	-3.365794
C	-0.878926	5.551646	-2.490789
H	-0.887483	0.030122	2.067886
H	1.664563	0.026965	2.925004
H	3.272675	0.005331	0.765628
H	1.729020	-0.012044	-1.433872
H	-1.357873	3.191563	0.864332
H	0.424849	3.368422	2.869569
H	2.899098	3.368107	1.811029
H	2.678669	3.180160	-0.860011
H	-3.025279	-2.738218	-3.050454
H	-3.784880	-1.476424	-2.069575
H	-2.839698	-1.048726	-3.514004
H	-1.862918	-3.699307	-1.050358
H	-0.847694	-2.699455	-0.004322

11-H

E(ωB97XD/6-31+G*)= -2989.662361

C	0.027704	0.039553	0.029310
C	0.040066	0.039361	1.454276
C	1.396245	0.036669	1.884902
C	2.240117	0.028898	0.724636
C	1.375555	0.031575	-0.419567
Fe	1.034170	1.678393	0.745207
C	0.043572	3.355147	0.098299
C	0.055651	3.295947	1.522036
C	1.411707	3.267747	1.952134
C	2.255848	3.315703	0.793028
C	1.391528	3.368894	-0.350125
P	4.076159	3.280857	0.620974
P	4.522239	1.634120	2.070648
P	4.060713	0.053453	0.553818
C	4.593425	-1.551512	1.412297
C	4.084835	-2.689239	0.510878
C	4.069079	-1.763947	2.836064

C	6.130524	-1.557165	1.421158
C	4.624047	4.843557	1.545350
C	6.161129	4.834089	1.554169
C	4.101449	5.001638	2.976811
C	4.126600	6.022607	0.692103
H	5.917295	1.633172	1.795370
H	1.709907	0.059475	-1.448823
H	-0.852382	0.077123	-0.599181
H	-0.830068	0.078459	2.096800
H	1.733709	0.085566	2.911582
H	1.725805	3.380634	-1.379716
H	-0.836706	3.352175	-0.531037
H	-0.814947	3.238482	2.162550
H	1.748481	3.172958	2.975824
H	6.536696	5.792346	1.935683
H	6.559741	4.044964	2.202674
H	6.571024	4.688185	0.547930
H	4.545658	5.896950	3.432772
H	3.015187	5.129648	2.992874
H	4.358153	4.142778	3.607018
H	4.468857	6.967784	1.134030
H	4.510776	5.970925	-0.333240
H	3.031816	6.051379	0.646776
H	6.496814	-2.534018	1.762405
H	6.541977	-1.373480	0.421765
H	6.536577	-0.799553	2.101830
H	4.504585	-2.681668	3.254307
H	4.333912	-0.934537	3.501377
H	2.981635	-1.882070	2.847033
H	4.417925	-3.655214	0.913051
H	2.989833	-2.705592	0.464638
H	4.469671	-2.598662	-0.511515

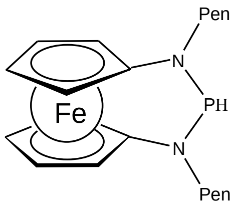

E(ωB97XD/6-31+G*)= -2495.061396

C	-1.699826	2.765566	-0.886132
C	-1.604897	1.365128	-1.130749
C	-1.553615	0.690488	0.132384
C	-1.614538	1.690885	1.151206
C	-1.695295	2.966774	0.523870
Fe	0.000050	1.981596	-0.059945
C	1.700089	2.765643	-0.885663
C	1.605242	1.365228	-1.130406
C	1.553680	0.690483	0.132672
C	1.614330	1.690797	1.151588
C	1.695192	2.966734	0.524363
N	1.394695	-0.701432	0.338322
C	2.111598	-1.599418	-0.567880
C	3.592770	-1.850814	-0.210141

C	3.692157	-2.450990	1.196578
N	-1.394662	-0.701420	0.338140
C	-2.111690	-1.599447	-0.567917
C	-3.592847	-1.850728	-0.210035
C	-4.414367	-0.555879	-0.277181
P	-0.000054	-1.327750	1.137782
C	4.141785	-2.849072	-1.239667
C	4.414364	-0.556000	-0.277021
C	-4.141949	-2.849189	-1.239314
C	-3.692194	-2.450587	1.196816
H	2.049157	-1.230801	-1.601777
H	-1.591856	-2.565104	-0.551246
H	-2.049359	-1.230883	-1.601836
H	1.591732	-2.565057	-0.551205
H	1.562772	1.504258	2.216400
H	1.733298	3.921072	1.032807
H	1.741578	3.539070	-1.641017
H	1.549935	0.897234	-2.104073
H	-1.563269	1.504378	2.216038
H	-1.733540	3.921132	1.032267
H	-1.741129	3.538888	-1.641603
H	-1.549313	0.897126	-2.104398
H	5.197046	-3.068998	-1.040418
H	3.590913	-3.797551	-1.211206
H	4.071854	-2.446680	-2.258376
H	4.737541	-2.662097	1.452054
H	3.291215	-1.758136	1.944142
H	3.128964	-3.389856	1.269045
H	5.475367	-0.771978	-0.100909
H	4.325493	-0.076975	-1.259783
H	4.090243	0.168572	0.476168
H	-5.475376	-0.771742	-0.100971
H	-4.090156	0.168856	0.475813
H	-4.325500	-0.077091	-1.260059
H	-4.737580	-2.661522	1.452425
H	-3.129100	-3.389499	1.269441
H	-3.291129	-1.757613	1.944205
H	-5.197179	-3.069123	-1.039896
H	-4.072166	-2.446981	-2.258105
H	-3.591044	-3.797645	-1.210753
H	-0.000091	-0.384142	2.189188

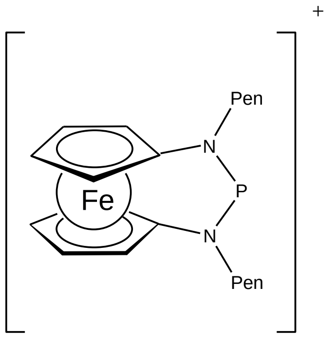

G(ωB97XD/6-31+G*)= -2493.814619
E(ωB97XD/6-31+G*)= -2494.236101

C	1.694954	2.681868	0.943526

C	1.723526	3.001392	-0.444180
C	1.621252	1.793393	-1.185886
C	1.505504	0.725066	-0.240924
C	1.565948	1.272492	1.079911
Fe	-0.000023	1.968204	0.023856
C	-1.695021	2.681828	0.943517
C	-1.723596	3.001348	-0.444190
C	-1.621290	1.793351	-1.185891
C	-1.505518	0.725029	-0.240926
C	-1.565981	1.272456	1.079907
N	-1.359628	-0.652805	-0.589497
P	0.000022	-1.535477	-0.813560
N	1.359649	-0.652772	-0.589495
C	2.612034	-1.389048	-0.949685
C	3.543162	-1.810097	0.208441
C	4.369182	-0.632730	0.747626
C	2.740594	-2.469202	1.335016
C	4.510861	-2.839523	-0.403560
C	-2.611993	-1.389112	-0.949688
C	-3.543125	-1.810137	0.208441
C	-2.740543	-2.469150	1.335059
C	-4.369198	-0.632774	0.747554
C	-4.510772	-2.839634	-0.403523
H	3.146911	-0.752487	-1.662454
H	-2.302797	-2.290783	-1.491895
H	-3.146873	-0.752584	-1.662485
H	2.302860	-2.290705	-1.491927
H	1.493403	0.714928	2.004009
H	1.735459	3.391805	1.758425
H	1.792468	3.995089	-0.865299
H	1.594008	1.691904	-2.262985
H	-1.493437	0.714894	2.004007
H	-1.735548	3.391767	1.758414
H	-1.792560	3.995043	-0.865311
H	-1.594039	1.691859	-2.262990
H	5.249133	-3.152249	0.341170
H	3.983918	-3.737113	-0.748025
H	5.058514	-2.416827	-1.254227
H	3.418384	-2.863500	2.098786
H	2.068330	-1.758267	1.829470
H	2.139979	-3.310520	0.964729
H	5.107021	-0.998514	1.469524
H	4.915659	-0.131271	-0.059678
H	3.758320	0.116546	1.254888
H	-5.107031	-0.998550	1.469462
H	-3.758373	0.116553	1.254784
H	-4.915685	-0.131381	-0.059784
H	-3.418324	-2.863437	2.098842
H	-2.139889	-3.310462	0.964820
H	-2.068313	-1.758162	1.829482
H	-5.249039	-3.152355	0.341214
H	-5.058434	-2.417003	-1.254217
H	-3.983787	-3.737218	-0.747938

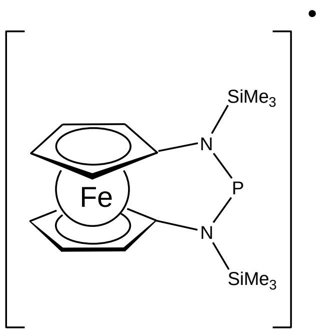

G(ωB97XD/6-31+G*)= -2918.364803
E(ωB97XD/6-31+G*)= -2918.699666

C	-0.002567	-0.013443	-0.004390
C	-0.016992	-0.010401	1.420428
C	1.334283	-0.003052	1.875106
C	2.184918	0.007398	0.733948
C	1.363011	-0.010248	-0.437249
Fe	0.988029	-1.636291	0.687731
C	0.090466	-3.203277	-0.224904
C	0.081796	-3.402921	1.185909
C	1.432992	-3.394367	1.640806
C	2.278340	-3.198590	0.512531
C	1.452221	-3.067845	-0.648490
N	1.896925	-2.810696	-1.969195
Si	1.516691	-4.004794	-3.229863
C	1.431634	-5.688310	-2.403163
N	1.816481	-0.057212	-1.779025
Si	1.363008	1.275545	-2.864322
C	1.189642	2.822208	-1.814227
P	2.759246	-1.372659	-2.379777
C	2.866161	-3.985086	-4.537344
C	-0.131173	-3.587076	-4.037666
C	-0.264211	0.881816	-3.724383
C	2.703448	1.510666	-4.160014
H	-0.775602	-3.113131	-0.867645
H	-0.797765	-3.515112	1.805929
H	1.759144	-3.499747	2.667141
H	3.357089	-3.110560	0.518168
H	-0.864601	-0.064547	-0.656758
H	-0.899022	-0.035648	2.046546
H	1.657986	-0.020971	2.907452
H	3.266969	-0.017883	0.731648
H	1.280759	-6.473796	-3.153828
H	2.359919	-5.907453	-1.863444
H	0.607868	-5.746993	-1.683721
H	2.680891	-4.787216	-5.263229
H	2.897784	-3.038492	-5.087148
H	3.855911	-4.151217	-4.097427
H	-0.358683	-4.282276	-4.855544
H	-0.958375	-3.634904	-3.320152
H	-0.109733	-2.573885	-4.455985
H	0.989449	3.693516	-2.449834
H	0.369048	2.734732	-1.094066
H	2.108002	3.017399	-1.248938

H	2.469217	2.393006	-4.769325
H	3.685118	1.670244	-3.700034
H	2.784385	0.651587	-4.834359
H	-0.533796	1.668253	-4.440505
H	-0.190245	-0.062462	-4.276532
H	-1.088722	0.786592	-3.008510

9⁺

G(ωB97XD/6-31+G*)= -5482.522360

E(ωB97XD/6-31+G*)= -5483.3245289

C	-5.042271	-1.378157	1.248640
C	-4.098583	-1.391372	2.313774
C	-2.804795	-1.519090	1.745733
C	-2.926576	-1.577870	0.316265
C	-4.329870	-1.482730	0.022062
Fe	-3.719670	0.166954	1.039184
C	-2.472312	1.753001	1.318042
C	-2.930198	1.680810	-0.038885
C	-4.363498	1.782822	-0.004783
C	-4.771512	1.908890	1.348665
C	-3.605579	1.884460	2.164704
P	-2.089754	1.345495	-1.604969
C	-1.351010	2.999535	-2.129322
C	-2.561235	3.931690	-2.332251
P	-1.462948	-1.972461	-0.711497
C	-2.068913	-2.705774	-2.344789
C	-0.808665	-3.155268	-3.105498
P	-0.441439	-0.089892	-1.289272
P	0.576016	0.011110	0.740131
N	1.753097	1.341769	0.690767
C	2.165701	1.724310	2.064154
C	2.028549	3.219541	2.424438
C	2.690686	4.140726	1.392631
C	2.726119	1.499272	-0.288764
C	4.149015	1.548162	-0.085501
C	4.800947	1.584794	-1.346181
C	3.797096	1.523568	-2.355357
C	2.533753	1.457986	-1.706104
Fe	3.714348	-0.161634	-1.188406
C	4.714452	-1.960943	-1.155570
C	4.039048	-1.770587	0.079024
C	2.624187	-1.681870	-0.165303
C	2.459262	-1.775379	-1.582148
C	3.732423	-1.953204	-2.189638
N	1.647999	-1.393121	0.778140
C	1.933712	-1.856112	2.157264
C	1.671546	-3.351837	2.445359
C	2.345229	-4.278734	1.425952
C	0.163048	-3.621878	2.450370
C	2.247392	-3.627164	3.842899
C	0.542326	3.573590	2.544737
C	2.710655	3.404518	3.787889
C	-0.660232	2.795405	-3.485640
C	-0.406903	3.635745	-1.109940

C	-2.893098	-1.794032	-3.260158
C	-2.881525	-3.956113	-1.959669
H	-4.781069	-1.472179	-0.959396
H	-6.113523	-1.263231	1.347979
H	-4.323797	-1.288816	3.366910
H	-1.870753	-1.538826	2.290768
H	-5.015884	1.731127	-0.867231
H	-5.792620	1.968532	1.701256
H	-3.586834	1.922860	3.246045
H	-1.448306	1.666526	1.650610
H	-3.769865	-1.372085	-2.764732
H	-3.240759	-2.372909	-4.125749
H	-2.298765	-0.955929	-3.635736
H	-3.148755	-4.505089	-2.870819
H	-3.809366	-3.704437	-1.438509
H	-2.304244	-4.632317	-1.318703
H	-0.173508	-3.809396	-2.497040
H	-0.215166	-2.296873	-3.442237
H	-1.105393	-3.713249	-4.001864
H	0.177086	2.092098	-3.432645
H	-0.267457	3.752737	-3.849692
H	-1.361596	2.412782	-4.235117
H	-0.016154	4.586312	-1.496863
H	0.444944	2.993629	-0.869574
H	-0.939498	3.851353	-0.178800
H	-3.281008	3.519701	-3.048804
H	-2.211400	4.893642	-2.727199
H	-3.084654	4.127222	-1.390786
H	1.519270	-1.683733	-2.105361
H	3.908938	-2.077831	-3.249646
H	5.781282	-2.090607	-1.279253
H	4.524139	-1.694432	1.041710
H	1.583066	1.334469	-2.200101
H	3.952575	1.535560	-3.425820
H	5.869564	1.653240	-1.500246
H	4.660046	1.547784	0.866059
H	1.296693	-1.271660	2.828212
H	2.967775	-1.601061	2.425436
H	-0.287532	-3.369877	1.485365
H	-0.337599	-3.038860	3.233387
H	-0.035610	-4.680378	2.651872
H	1.809599	-2.959860	4.595540
H	3.336366	-3.494709	3.864410
H	2.031524	-4.656467	4.148163
H	3.429835	-4.124546	1.380441
H	1.930597	-4.142112	0.420988
H	2.174820	-5.323077	1.709039
H	3.189682	1.378464	2.252948
H	1.533314	1.161480	2.757451
H	0.053661	2.979268	3.326677
H	0.021101	3.393386	1.602145
H	0.413680	4.631194	2.799139
H	3.788634	3.210721	3.726493
H	2.286757	2.731710	4.543428
H	2.577118	4.430493	4.146633

H	2.582506	5.184717	1.706127
H	2.225559	4.047048	0.405303
H	3.763379	3.939562	1.290319
H	-1.735548	3.391767	1.758414
H	-1.792560	3.995043	-0.865311
H	-1.594039	1.691859	-2.262990
H	5.249133	-3.152249	0.341170
H	3.983918	-3.737113	-0.748025
H	5.058514	-2.416827	-1.254227
H	3.418384	-2.863500	2.098786
H	2.068330	-1.758267	1.829470
H	2.139979	-3.310520	0.964729
H	5.107021	-0.998514	1.469524
H	4.915659	-0.131271	-0.059678
H	3.758320	0.116546	1.254888
H	-5.107031	-0.998550	1.469462
H	-3.758373	0.116553	1.254784
H	-4.915685	-0.131381	-0.059784
H	-3.418324	-2.863437	2.098842
H	-2.139889	-3.310462	0.964820
H	-2.068313	-1.758162	1.829482
H	-5.249039	-3.152355	0.341214
H	-5.058434	-2.417003	-1.254217
H	-3.983787	-3.737218	-0.747938

4^{+}

G(ωB97XD/6-31+G*)= -5977.153800
E(ωB97XD/6-31+G*)= -5977.8900333

C	-5.424866	-2.107020	0.721457
C	-5.696262	-2.070253	-0.677702
C	-4.464339	-1.848679	-1.353583
C	-3.409664	-1.743909	-0.386495
C	-4.025991	-1.899080	0.902447
Fe	-4.829936	-0.304484	-0.054850
C	-4.391542	1.213228	1.214885
C	-3.807538	1.463002	-0.073219
C	-4.892363	1.508699	-1.010128
C	-6.114573	1.291585	-0.315311
C	-5.805396	1.113464	1.065249
P	-2.057295	1.771709	-0.579515
C	-1.734056	3.350311	0.458779
C	-2.931674	4.287828	0.217381
P	-1.633045	-1.589038	-0.896443
C	-1.015285	-3.252079	-0.171462
C	-1.960834	-4.322018	-0.751927
P	-1.069747	0.078349	0.537553
P	1.049255	0.696140	0.172488
P	1.933769	-0.730707	1.694841
C	1.606764	0.048805	3.409054
C	1.881192	1.552539	3.533665
C	3.713490	-0.386595	1.515346
C	4.452938	0.842185	1.571793

C	5.836873	0.536089	1.464622
C	5.971368	-0.874984	1.333283
C	4.671927	-1.444911	1.367575
Fe	4.798648	-0.115720	-0.171466
C	5.596768	0.903583	-1.752434
C	4.216714	1.200906	-1.586485
C	3.475421	-0.022740	-1.698271
C	4.428708	-1.069109	-1.934955
C	5.727386	-0.497951	-1.963534
P	1.687021	-0.362556	-1.735508
C	1.160457	0.719300	-3.216570
C	-0.251659	0.293344	-3.643540
C	2.133053	0.348427	-4.352577
C	1.187092	2.230783	-2.986790
C	2.552033	-0.717844	4.354626
C	0.162553	-0.239624	3.848968
C	-1.543865	3.127148	1.961538
C	-0.481956	4.032051	-0.118691
C	0.395135	-3.511535	-0.721666
C	-0.979323	-3.363204	1.354284
H	-4.778625	1.641065	-2.078794
H	-7.098460	1.223050	-0.760944
H	-6.513770	0.887911	1.851817
H	-3.841794	1.064093	2.134125
H	-3.519783	-1.809032	1.853530
H	-6.158952	-2.217322	1.509129
H	-6.672252	-2.148301	-1.138813
H	-4.331458	-1.737187	-2.422408
H	-1.384664	4.095803	2.451420
H	-0.671391	2.504382	2.175801
H	-2.417313	2.666753	2.432715
H	0.412255	3.412019	-0.014500
H	-0.305885	4.966966	0.427199
H	-0.611918	4.285114	-1.176186
H	-3.156784	4.400831	-0.850053
H	-2.683972	5.282504	0.605862
H	-3.839693	3.954558	0.729596
H	0.686807	-4.536213	-0.459481
H	1.131889	-2.833931	-0.286241
H	0.439374	-3.410856	-1.810472
H	-0.321580	-2.610587	1.796527
H	-0.578990	-4.347442	1.627681
H	-1.970198	-3.285781	1.809522
H	-2.967441	-4.271849	-0.323824
H	-1.555320	-5.312032	-0.513450
H	-2.041569	-4.255208	-1.843468
H	4.429605	-2.495699	1.274543
H	6.898663	-1.414641	1.195028
H	6.643699	1.256562	1.437372
H	4.029965	1.835553	1.624232
H	4.185748	-2.117927	-2.046706
H	6.656414	-1.038077	-2.087625
H	6.410343	1.613622	-1.682748
H	3.806535	2.175755	-1.361184
H	2.367094	-1.798223	4.328153

H	2.382299	-0.375924	5.383187
H	3.605143	-0.544594	4.114611
H	0.057802	0.011314	4.912092
H	-0.099360	-1.297137	3.729838
H	-0.566610	0.355200	3.293327
H	1.392999	2.134514	2.744726
H	2.951099	1.769819	3.505972
H	1.502641	1.912200	4.499200
H	3.146123	0.715262	-4.168744
H	1.773801	0.798872	-5.286121
H	2.185749	-0.735822	-4.508728
H	0.918655	2.747463	-3.917715
H	2.174691	2.588153	-2.684760
H	0.463645	2.529522	-2.224661
H	-0.311759	-0.785341	-3.823486
H	-0.517646	0.810136	-4.574905
H	-1.003821	0.556498	-2.895910
